# PFAS in the Urban Atmosphere Detected with I‑CIMS and LC-ESI Orbitrap Mass Spectrometer

**DOI:** 10.1021/acsestair.6c00191

**Published:** 2026-09-09

**Authors:** Lee Tiszenkel, Vignesh Vasudevan-Geetha, Zachary Watson, James Flynn, Shan-Hu Lee

**Affiliations:** † Dept of Atmospheric Science, 14843 University of Alabama at Huntsville, Huntsville, Alabama 35899, United States; ‡ Department of Earth and Atmospheric Sciences, 14743 University of Houston, Houston, Texas 77204, United States

**Keywords:** PFAS, PFCA, iodide chemical ionization mass spectrometry, LC-ESI Orbitrap MS, non-targeted analysis, urban air quality, atmospheric monitoring

## Abstract

Per- and polyfluoroalkyl substances (PFAS) are persistent pollutants whose atmospheric fate in urban areas remains poorly characterized. We combined in-situ iodide CIMS with targeted offline LC-ESI Orbitrap MS to quantify 22 gas- and particle-phase PFAS in Houston, Texas. We calibrated I-CIMS for 18 PFAS and validated it against LC-ESI Orbitrap MS across 9 co-detected particle-phase compounds. Gas-phase ΣPFAS reached 2.2 pptv (17.7 ng m^–3^) and particle-phase ΣPFAS reached 0.25 pptv (3.1 ng m^–3^), with 4:2 FTOH, PFPrA, and TFA peaking at 2 to 3 pptv in the gas-phase, among the highest reported in urban atmospheres. Gas-phase PFAS correlated with temperature and ozone, while particle-phase PFAS correlated with humidity. The relationship between particle-associated fraction and subcooled liquid vapor pressure was shifted roughly two orders of magnitude relative to wintertime Toronto, and a further two orders relative to indoor environments, demonstrating that aqueous aerosol modulates PFAS gas-particle equilibrium under humid subtropical conditions. Comparison with Huntsville, Alabama, revealed an inverted PFCA chain-length distribution, demonstrating that local emissions can override regional and seasonal trends in PFAS composition. These results extend the atmospheric PFAS gas-particle partitioning framework to subtropical urban environments and demonstrate the value of combined online-offline measurements for characterizing urban PFAS loadings.

## Graphical abstract

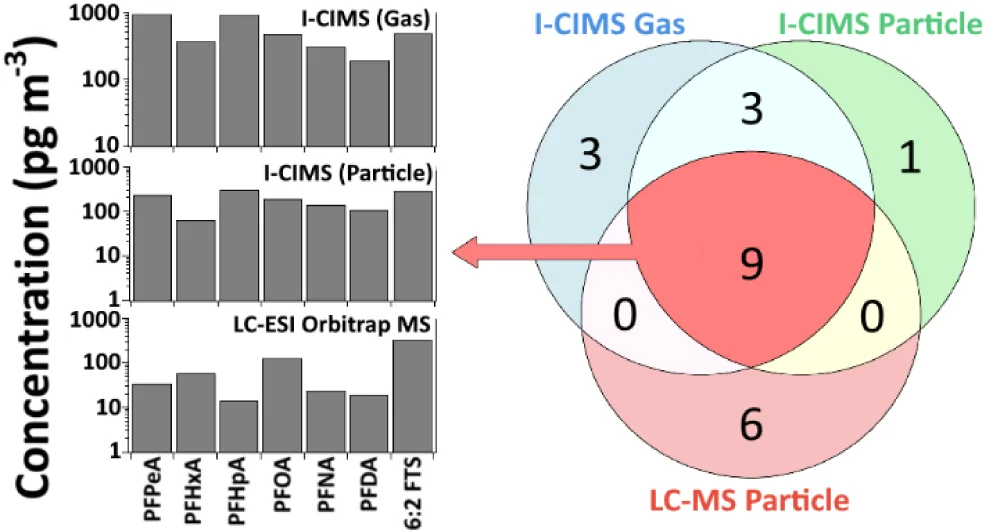

## 1 Introduction

Per- and polyfluoroalkyl substances (PFAS) are human-synthesized compounds containing at least one fully fluorinated methyl or methylene carbon atom.^1–3^ PFAS are used in consumer and industrial applications including stainproof or waterproof textiles, grease resistant food contact surfaces, fluoropolymer manufacturing aids, and aqueous film-forming foams widely used to extinguish fires involving flammable liquids.^1,4–7^ PFAS exposure is associated with deleterious health effects including endocrine disruption,^8^ immunotoxicity,^9,10^ preeclampsia,^11^ neurodevelopmental issues,^12^ and increased cancer risk.^13,14^ Humans may be exposed to PFAS via contaminated water or food,^15,16^ but inhalation of gas-phase PFAS or PFAS-containing particles in ambient and indoor air is a potentially important exposure pathway,^17–20^ though the magnitude remains poorly constrained due to limited measurements of atmospheric PFAS concentrations and composition in population centers.

PFAS are emitted into the atmosphere in both the gas- and particle-phase during manufacturing, industrial/personal use, and disposal of PFAS-containing substances.^1,21–23^ Residences and other indoor environments are also sources of atmospheric PFAS through air exchange with buildings containing PFAS-treated materials and consumer products.^18,19^ In the atmosphere, volatile and semi-volatile PFAS volatilize from surfaces, persisting as stable gas-phase pollutants.^24–28^ PFAS are also highly water-soluble and exhibit higher concentrations near the air/water surface,^29,30^ thus they can efficiently partition into preexisting atmospheric particles (including sea spray aerosol) and rain droplets.^31–34^ Gas- and particle-phase PFAS loadings are elevated near direct emission sources such as fluoropolymer production facilities,^21^ military installations,^35^ and urban areas,^33^ but they are also frequently detected in remote Arctic^36–39^ and marine^38,40^ atmospheres due to their stability during long-range transport.^28^ A comprehensive understanding of emission sources, gas-particle partitioning, and eventual fate of atmospheric PFAS is critical for developing and implementing measures to mitigate atmospheric PFAS pollution.

In recent years, atmospheric concentrations of short-chain (≤7 carbon), ultra-short-chain (≤3 carbon), and emerging PFAS like perfluoroalkyl ether carboxylic acids (PFECAs) have increased due to regulations on long chain (≥8 carbon) and legacy PFAS such as perfluorooctanoic acid (PFOA) and perfluorooctanesulfonic acid (PFOS).^5,41,42^ Measurements across Asia found that perfluorohexanoic acid (PFHxA, a short-chain PFAS), hexafluoropropylene oxide dimer acid (HFPO–DA, also known as GenX, a PFOA replacement compound), and *N*-ethylperfluorooctylsulfonamide (N-EtFOSA, also known as sulfluramid, a PFAS precursor compound) were detected at comparable concentrations to legacy PFAS in India, South Korea and Japan.^43^ The ultra-short-chain PFAS trifluoroacetic acid (TFA) is increasingly abundant in the atmosphere in population centers due to its formation as a degradation product of commonly used freon alternatives in air conditioners, in addition to photochemical degradation mechanisms.^1,44–47^ TFA has been measured in air at concentrations of 1–2 parts per trillion by volume (pptv) in Toronto,^48,49^ Beijing,^50,51^ New Jersey,^52^ and Reno, Nevada.^53^ The increasing atmospheric relevance of emerging and ultra-short-chain PFAS makes quantification of total PFAS loadings in populated areas challenging because these compounds are usually not included in targeted analyses.

Researchers have primarily used targeted offline high-resolution LC-MS (liquid chromatography mass spectrometer) tandem MS/MS analysis for ambient PFAS measurements, which is effective due to its extremely high mass resolution (m/Δm up to 120,000) and detection limits in the pg m^–3^ range.^28,32,54,55^ The U.S. EPA has developed standard PFAS detection methods with high-resolution LC-MS (such as Method 1633) for water, soil, and solid sample analysis.^56^ Recently, EPA methods have been adopted for targeted and semi-non-targeted detection of airborne PFAS in the gas phase, aerosols and rain droplets collected with sorbent tubes, annular denuders, or high-volume filter collection.^32,34,54,57,58^ However, sample collection, storage, and extraction for LC analysis can produce artifact PFAS signals or be susceptible to reactions in filter substrates that can alter PFAS profiles.^59^ Targeted analyses are inadequate for the rapidly expanding PFAS chemical landscape (currently over 7 million known compounds^60^). Also, offline analyses depend on composite samples with weekly, monthly, or seasonal time resolutions.^34,58^ These time scales are too long to present a complete picture of the sources and fates of atmospheric PFAS, which depend on processes on the scale of seconds to minutes.

Chemical ionization mass spectrometry (CIMS) addresses these instrumental shortcomings. CIMS is a non-targeted and fast response (<60 second) measurement technique with certain reagent chemistries (especially, e.g., iodide or acetate) that are highly sensitive to PFAS.^61–65^ Previous studies have calibrated CIMS for select PFAS compounds.^49,52,62,63,66^ However, CIMS provides less definitive compound identification than LC-MS/MS due to its relatively limited mass resolution in the high-resolution time-of-flight (HR-ToF) mass spectrometer (m/Δm ∼ 7,000). Additionally, the use of Teflon in the CIMS inlet or ion-molecule reactor (IMR) can cause internal fluorinated artifacts within the instrument. Currently, studies that have utilized CIMS for online measurements of ambient atmospheric PFAS are still extremely limited. Measurements of perfluorocarboxylic acids (PFCAs) with acetate CIMS in the Toronto metropolitan area during the summer and winter found gas-phase concentrations of PFCAs in the pptv or sub-pptv range, with TFA being the most abundant.^49^ In semi-rural New Jersey, iodide CIMS (I-CIMS) was used to detect gas-phase PFAS and found TFA at the pptv level, although TFA was rarely above the limit of detection.^52^ These limited studies demonstrate CIMS sensitivity to atmospheric PFAS but highlight the need for expanded measurements in diverse environments.

Here, we investigated ambient atmospheric PFAS in an urban U.S. population center with non-targeted in-situ gas- and particle-phase I-CIMS attached to a filter inlet for gases and aerosols (FIGAERO^67^). We have validated the I-CIMS findings with targeted offline particle-phase LC-ESI Orbitrap MS (based on EPA Method 1633^56^). Houston is the 4th most populated city in the United States, with both coastal and continental atmospheric influence and abundant emissions from residential, commercial, and industrial activity.^68,69^ Our objective was to quantify atmospheric PFAS in the gas- and particle-phase and understand diurnal variations and gas-to-particle partitioning behavior of PFAS in urban Houston. To examine how PFAS profiles vary between different urban environments in the United States, we compared our filter-collected samples from Houston and those collected in Huntsville, Alabama, the most populated metropolitan area in Alabama with influence from military and fluoropolymer industry emissions. These observations advance our understanding of atmospheric PFAS dynamics in urban environments.

## 2 Materials and Methods

### 2.1 Measurement Sites

Field measurements were conducted continuously from 8 to 27 October 2022 near the urban center of Houston.^70,71^ Houston is the 4th largest city in the U.S. with a population of 2.4 million. Measurements were made on a stationary platform located on the campus of the University of Houston (29.72° N, 95.34° W, 11 m above sea level). To the southeast (∼300 m) was an industrial park with chemical supply, automotive, and machining facilities. The city center was approximately 2.5 km away to the northeast. Maps of the measurement site are shown in Figure S1. There were collocated measurements of meteorological parameters (temperature, RH, wind speed/direction), key atmospheric pollutants (CO, SO_2_, O_3_, NO_*x*_, and ammonia), and aerosol number size distributions as described in Tiszenkel et al. (2025).^70^

To validate the online I-CIMS measurements and to examine how PFAS profiles vary between urban environments, we collected particle-phase samples for offline LC-ESI Orbitrap MS analysis at the Houston measurement platform as well as in Huntsville, Alabama. Huntsville is the largest city in Alabama with a population of 250,000. The Huntsville sampling location was on the roof of the Severe Weather Institute and Radar & Lightning Laboratories (SWIRLL) building on the campus of the University of Alabama at Huntsville (34.72 ° N, 86.65 ° W, 210 m above sea level).^72^ This site was located approximately 5 km north of a major United States Army installation (Redstone Arsenal), 6 km east of Huntsville city center, and 25 km northeast of Decatur, Alabama, where two major fluoropolymer manufacturing facilities (3M and Daikin) are located.

### 2.2 High Resolution Time-of-Flight Iodide Chemical Ionization Mass Spectrometer

#### 2.2.1 Instrument Overview

HR-ToF CIMS (Tofwerk, Switzerland)^73^ attached to a FIGAERO inlet (Aerodyne Inc.)^67^ was used to detect PFAS in the gas- and particle-phase. Complete inlet configuration and sampling protocol details are provided in Supporting Information Section S1.1. Briefly, 2.5 standard liters per minute (slpm) of pure nitrogen from the headspace of a liquid nitrogen dewar was flowed past a permeation tube of methyl iodide (Kin-Tek) in a permeation oven held at 35 °C. This methyl iodide and nitrogen gas mixture then flowed through a ^210^Po source before entering the IMR where iodide ions deprotonated or formed iodide adducts with analytes. Ions were then directed through a series of differentially pumped chambers to the time-of-flight (ToF) mass analyzer. The HR-ToF CIMS was tuned to maximize its response to oxidized organic molecules with the Thuner automatic mass spectrometer tuning software (Tofwerk, Switzerland). Voltages for the inlet regions and ion lenses are shown in Table S1. All ion signals were normalized to the sum of the primary reagent ions (I^–^, I·H_2_O^–^, and IO^–^) scaled to 10^6^ counts per second of total reagent ion signal.^74^ Reported calibration factors and concentrations use this normalization throughout.

Limits of detection (LOD) for PFAS measured with the I-CIMS were calculated for each background period based on three times the standard deviation of the background measurement and calibration factors, which resulted in a dynamic LOD for each compound.^49,75^ The instrument background during measurement periods was used to calculate a dynamic LOD because CIMS frequently detects the presence of fluorinated contaminants that may interfere with measurements of PFAS compounds.^76,77^

#### 2.2.2 Calibration

Accurate quantification of PFAS with CIMS requires compound-specific calibration, but previous studies have calibrated only a small subset of PFAS compounds.^49,52,62,63^ We calibrated I-CIMS for 18 PFAS spanning five compound classes: 9 perfluorocarboxylic acids (C2–C10 PFCAs), 1 perfluoroalkyl ether carboxylic acid (DONA), 3 fluorotelomer alcohols (4:2, 6:2, and 8:2 FTOH), 2 perfluorosulfonic acids (PFBS and PFOS), and 3 fluorotelomer sulfonates (4:2, 6:2, and 8:2 FTS). Individual compound structures, purities, concentrations, and sourcing details are provided in Tables S2 and S3.

A combined calibration standard containing all 18 compounds was prepared by diluting 2.5 μL of each PFAS standard compound (Table S2) in 250 mL of methanol, yielding concentrations between 1.9 × 10^–5^ and 1.3 × 10^–4^ M (Table S3). The combined calibration standard was prepared from a mixture of pure PFCA compounds and pre-prepared methanolic solutions of fluorotelomers, PFSAs, and PFECAs containing NaOH (Cambridge Isotope Laboratories). The actual concentrations of PFCAs and PFSAs in the combined I-CIMS calibration standard were independently quantified by LC-ESI Orbitrap MS analysis following the procedure described in Section 2.3. These LC-MS-derived concentrations were used to construct the I-CIMS calibration curves rather than the nominal preparation concentrations, which accounts for any analyte losses during preparation including potential methyl ester formation from PFCAs in methanol.^56^ The calibration standard was stored at 4 °C when not in use. Calibration was performed by first wetting the filter with 5 μL methanol followed by direct deposition of 0.2–5 μL of the calibration standard on the FIGAERO filter for a total mass loading on the filter between 3–100 ng for each compound. The filter was then subjected to a 2 slpm flow of heated N_2_ to generate gas-phase compounds for detection.^61^ Table S4 shows the program for the FIGAERO desorption cycle used for calibration. Section S1.3 provides additional details on calibration backgrounds and error analysis.

#### 2.2.3 QA/QC

Reliable detection of atmospheric PFAS with I-CIMS requires careful QA/QC because fluorinated contaminants within the instrument and sensitivity to environmental variables can introduce systematic biases. Mass calibration of the HR-ToF was performed using ions spanning the mass range achieving a mass accuracy of less than 5 ppm for all PFAS compounds reported in this study. PFAS identification required both accurate mass matching and peak fitting (within 2 ppm of the expected exact mass; >98% peak area fit) and confirmation of the iodide adduct and/or conjugate base for each compound (Figure S2).

To assess potential contamination from the PTFE components of the FIGAERO inlet and sampling lines, we monitored PFAS signals during background periods and tracked their variability throughout the measurement campaign. Background signals for all reported PFAS compounds were stable throughout the measurement period (Figure S3) and the background-subtracted ambient signals were consistently elevated above the dynamic LOD for all detected compounds in both gas- and particle-phase measurements (Figure 1). Particle-phase backgrounds were generally lower than gas-phase backgrounds, and the resulting particle-phase LODs were comparable to or lower than gas-phase LODs for most compounds (Figure 1b–c). The diurnal cycles of background measurements were checked to ensure they were not influenced by environmental factors (Figure S4).

**Figure 1:**
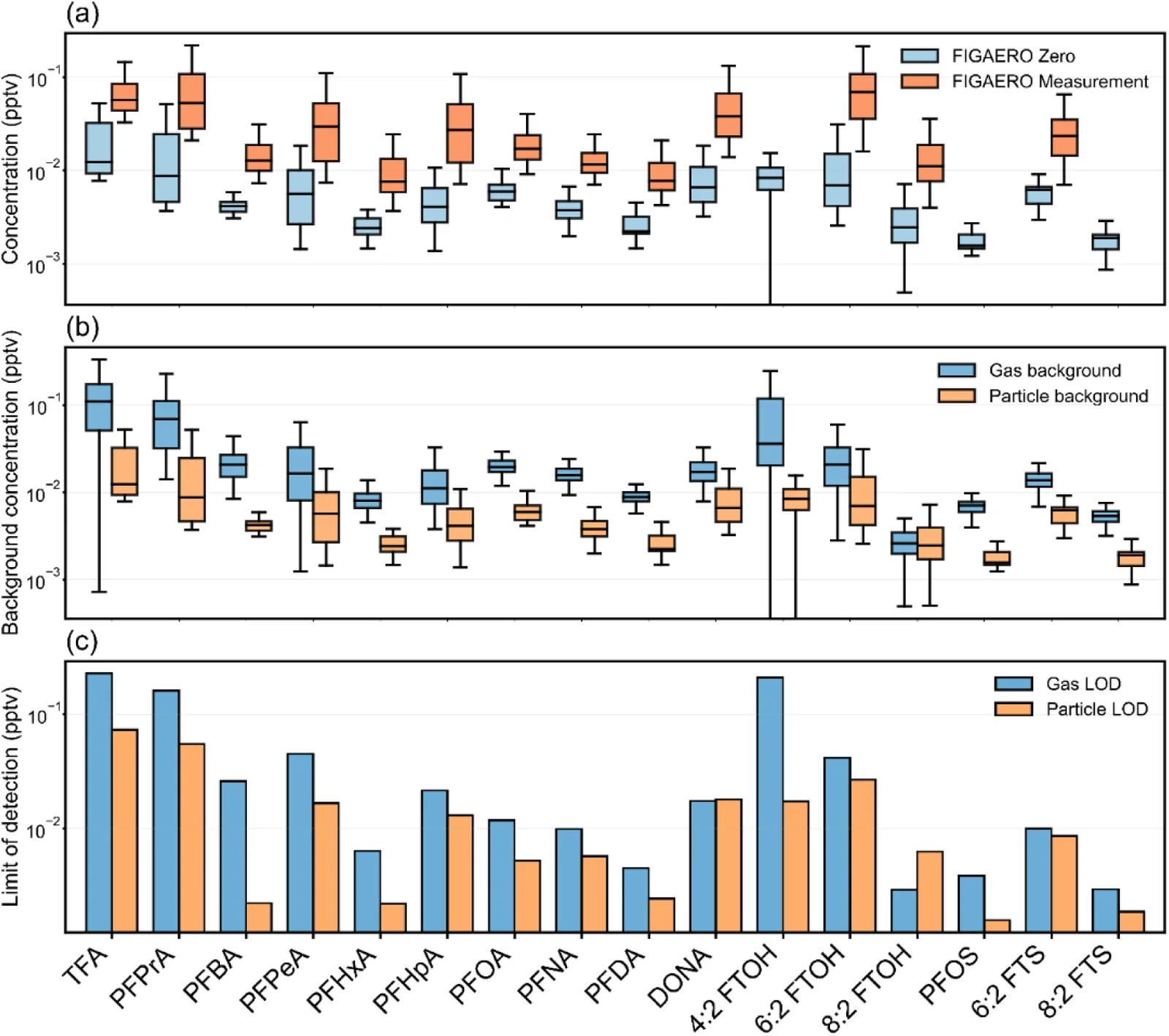
I-CIMS background characterization during the Houston campaign. (a) Comparison of background-subtracted ambient signals (orange) and zero-cycle backgrounds (blue) for each detected PFAS compound, demonstrating that ambient signals exceed backgrounds across the measured compound range. (b) Distribution of gas-phase and particle-phase background signals throughout the measurement campaign. (c) Mean gas-phase and particle-phase limits of detection (LOD) for each compound, calculated as 3σ of the dynamic background.

### 2.3 Liquid Chromatography Electrospray Ionization Orbitrap Mass Spectrometer (LC-ESI Orbitrap MS)

#### 2.3.1 PFAS Sample Preparation and Analysis

Particle-phase samples were collected onto pre-baked (500 °C) quartz fiber filters. In Houston, the filter samples were collected daily from 25 September to 26 October 2022 (except for 5 days in September). The filter sampling inlet was fitted with a 2.5 μm cyclone impactor. Each sample was collected for 24 h with a flow rate of 1 L min^–1^. Filters were stored in borosilicate glass vials at −25 °C after collection. 14 days (13 October to 26 October 2022) of filters were combined to make one sample to ensure there was sufficient mass on the filter for LC-ESI Orbitrap MS analysis. In Huntsville, samples were collected for 2 weeks at a flow rate of 10 L min^–1^ from 30 June to 28 July 2025.

The LC-ESI Orbitrap MS workflow strictly followed EPA Method 1633 for analysis of PFAS compounds in environmental samples.^56^ Figure S5 shows the workflow chart. A complete description of the LC-ESI workflow is given in Section S2. Briefly, filter samples were spiked with extracted internal standards (EIS), extracted with methanolic ammonium hydroxide, and purified via solid-phase extraction with WAX/GCB cartridges. EIS represents the labeled standards added prior to extraction to account for losses during sample preparation. The samples were analyzed with ultra performance liquid chromatography – electrospray ionization mass spectrometry (LC-ESI-MS/MS) using a Thermo Fisher Vanquish LC system coupled with Orbitrap Exploris 120 mass spectrometer. The Vanquish LC system was modified to reduce PFAS interferences by replacing the standard Teflon plumbing with polyether ether ketone (PEEK) tubing and installing a delay column.

#### 2.3.2 Calibration and QA/QC

Native analytical standards, EIS, and non-extracted internal standards (NIS) were obtained from Cambridge Isotope Laboratories (Tewksbury, MA, USA). NIS represents the labeled standards added after extraction to monitor instrument sensitivity, as opposed to EIS. The LC-ESI Orbitrap MS was calibrated for 9 PFCAs (PFBA, PFPeA, PFHxA, PFHpA, PFOA, PFNA, PFDA, PFUdA, and PFDoA), 5 PFSAs (PFBS, PFPeS, PFHxS, PFHpS, PFOS), 3 FTSs (4:2, 6:2 and 8:2 FTS), 5 PFECAs (GenX, DONA, PFMPA, PFMBA, and NFDHA), and 3 PFESAs (9Cl-PF3ONS, 11Cl-PF3OUdS, and PFEESA). A full description of the calibration standards including molecular formula, and CAS numbers is shown in Table S5. Extraction efficiencies of each analyte were corrected using isotope labeled standards spiked on to the filter prior to extraction. The recoveries for the analytes ranged from 58–163% (Tables S6 and S7, Figure S6). The analytical detection limits (ADL) were determined following EPA method 1633 by running a low concentration calibration standard (0.5 ng/mL) seven times in succession (Table S8). The field blanks were determined by subjecting the pre-baked quartz filter to the exact sample-extraction procedure, and the field blank values were subtracted from sample values for analytes where the mean blank was significantly higher than 0. As shown in Table S9, the method detection limit (MDL) for an analyte is the larger of the analytical detection limit (ADL) or the sum of the average and 3σ of the blank values (MDL_b_).

## 3 Calibration Results

### 3.1 LC-ESI Orbitrap MS

LC-ESI Orbitrap MS provides high mass accuracy and sensitivity for targeted quantification of PFAS in ambient filter samples. Quantification of each target analyte was achieved using isotope dilution with authentic stable-isotope-labeled (SIL) internal standards and a 7-point external calibration curve spanning 1 to 50000 pg mL^–1^ as shown in Figure S7. Calibration curves were fit using inverse concentration weighting (1/x) and were not forced through zero. The 1/x weighting scheme reduced the disproportionate influence of high-concentration calibration points on the regression curve, which improved accuracy at the low-concentration end of the calibration most relevant to ambient filter measurements. R^2^ > 0.99 and RSD values < 15% were achieved for all analytes (Figure S7), meeting EPA Method 1633 acceptance criteria and providing a robust regression fit across the calibration range. The best-fit regression curve was quadratic for most anayltes except for PFHpS, NaDONA and 9Cl-PF3ONS, which were best described by a linear fit. Quadratic calibration responses are commonly observed for PFAS in LC-MS analyses,^78–80^ which has been attributed in part to detector response compression at elevated ion loads.

### 3.2 I-CIMS

Compound-specific calibration is essential for quantitative measurements of PFAS with CIMS, yet calibration data still remain scarce across the diversity of atmospherically relevant PFAS. We calibrated I-CIMS for 18 compounds spanning PFCAs, FTOHs, PFSAs, FTSs, and one PFECA (Section 2.2.2). To our knowledge, we report the first I-CIMS calibration factors for perfluorononanoic acid (PFNA), perfluorodecanoic acid (PFDA), 4,8-dioxa-3H-perfluorononanoic Acid (DONA), 4:2 fluorotelomer sulfonate (FTS), 6:2 FTS, and 8:2 FTS. Calibration factors ranged from 23.2 to 141.7 counts per second (cps) per pptv (Figure S8). Results from each replicate calibration trial and calibration fit curves are shown in Figure S9, and the normalized mean calibration factors are summarized in Table S10. The calibration results showed that I-CIMS is highly sensitive to PFAS compared with other standards that the instrument has previously been calibrated for (Figure S9, Table S10). All 18 PFAS calibration factors exceeded that of formic acid previously calculated for this same instrument (6.7 cps pptv^–1^),^81^ and were within a factor of 1–6 of the I-CIMS N_2_O_5_ calibration factor (24 cps pptv^–1^), which represents the diffusion-limited reaction rate for the iodide IMR.^52,74^ The high sensitivity of I-CIMS to PFAS likely reflects the strong gas-phase acidity of fluorinated compounds, which favor efficient proton transfer to I^–^ for acidic PFCAs and PFSAs, and iodide adduct formation for neutral PFAS such as FTOHs.^61^ However, calibration factors varied substantially among compounds. PFCA calibrations also showed high variability among replicate calibration trials compared to FTOH (Figure S8), consistent with the greater influence of IMR conditions on detection of acidic compounds.^61,74,82,83^ Comparison with published CIMS calibration factors reveals that PFAS sensitivities can vary by as much as a factor of 10 between instruments, even when using the same IMR and inlet flow rates (Table S10). These discrepancies stress that CIMS measurements of atmospheric PFAS cannot rely on literature sensitivity values and underscore the importance of performing instrument-specific calibrations for measurements of PFAS instead of relying on literature values.

## 4 Validation of I-CIMS Measurements

To assess the reliability of online I-CIMS measurements, we compared particle-phase PFAS detected by I-CIMS with LC-ESI Orbitrap MS analysis of filter samples (Figure 2). Nine PFAS were detected by both methods: TFA, C4–C10 PFCAs and 6:2 FTS. The LC-ESI Orbitrap MS analysis followed the list of targeted compounds in EPA Method 1633 and did not include FTOHs, TFA, or PFPrA. For FTOHs specifically, spike-and-recovery testing using a mixture of 4:2, 6:2, 8:2, and 10:2 FTOH confirmed that the LC-ESI Orbitrap MS workflow is unable to detect FTOHs due to their low ESI ionization efficiency in negative mode.^84–87^ Thus, the absence of these compounds in the offline measurements reflects both target list scope and analytical limitations of LC-ESI MS and does not indicate their absence from the sample. For the 9 co-detected compounds, LODs were comparable between methods (Figure S10). TFA was considered a common compound between techniques because it produced a strong signal and identifiable MS/MS fragmentation spectrum (Figure S11), but it could not be quantified with LC-ESI Orbitrap MS without an authentic standard. PFBA was quantified by both methods and met all EPA Method 1633 quantification criteria, but the field blank and sample concentrations of PFBA were exceedingly high, introducing substantial bias into the measurements that precluded the use of PFBA for cross-validation. The overlap of 9 compounds between methods provides a robust basis for cross-validation.

**Figure 2:**
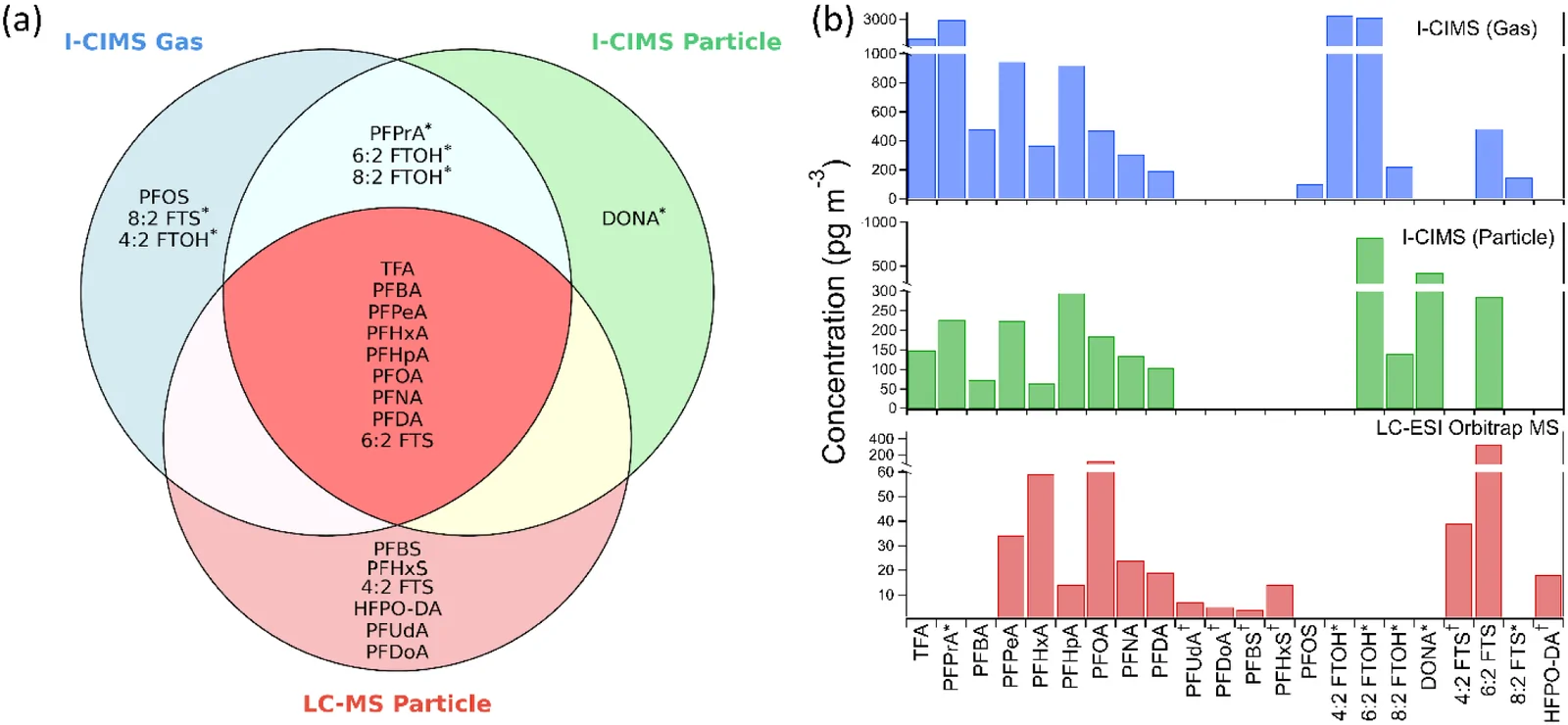
(a) Venn diagram of PFAS compounds detected by the I-CIMS in gas-phase (blue), by the I-CIMS in the particle-phase (green), and by the LC-ESI Orbitrap MS in the particle phase (red). Except for 6:2 FTS, all common PFAS between the I-CIMS and LC ESI Orbitrap MS were PFCAs. (b) bar graphs of median concentrations in pg m^3^ of PFAS detected by each instrument. * Denotes compounds that were not included in the LC-ESI Orbitrap MS targeted analysis. TFA was detected by LC-ESI Orbitrap MS and confirmed by MS/MS analysis, but quantification was not possible due to the lack of an authentic standard (Figure S11). † Denotes compounds that were not detected by I-CIMS.

LC-ESI Orbitrap MS detected 14 of the targeted 25 compounds above the LOD in Houston (Figure 2, Table S11) with a total particle-phase PFAS concentration of 692 ± 122 pg m^–3^. FTS were the most abundant class of PFAS, with 4:2 and 6:2 FTS combined contributing 52% of the total PFAS loading. PFCAs accounted for 45% of the total PFAS, and PFSAs including PFBS and PFOS constituted 3% of the total. PFOA and 6:2 FTS were the most abundant individual compounds in the filter samples, and these compounds were similarly abundant in particle-phase I-CIMS measurements. These results confirm that both methods identify the same dominant compounds.

Quantitative comparison revealed systematic differences between the two methods. Particle-phase concentrations measured by I-CIMS were higher by a factor of 2 to 8 compared to the LC-ESI Orbitrap MS (Figure 2). Several factors may contribute to this discrepancy. For LC-ESI Orbitrap MS, signals may be lower due to volatilization of semi-volatile PFAS from filters during sample collection, analyte loss during transport and storage, incomplete sample extraction from filters, or losses during SPE workup. Filter-based sampling losses of semi-volatile PFAS are well documented in atmospheric PFAS measurement literature.^88,89^ Despite the absolute concentration difference, the consistency of relative abundance patterns and compound identification between methods validates I-CIMS for relative composition, while absolute concentrations from both methods should be interpreted with these methodological caveats.

Despite these quantitative differences, concentration trends among PFAS classes were consistent between instruments. Both methods found elevated particle-phase PFOA relative to other long-chain compounds and measured abundant 6:2 FTS. The lack of short-chain PFCA dominance in the LC-ESI Orbitrap MS analysis is likely due to a lack of quantification for < C5 PFCAs, which were the most abundant compounds detected by the I-CIMS. The agreement in relative abundance patterns validates I-CIMS observations with established EPA methods and confirms that both techniques captured the PFAS distribution in urban Houston.

## 5 Ambient PFAS in Houston

In total, we detected 22 PFAS during the 19-day measurement campaign (Table S12). All 22 of our observed PFAS have previously been detected in urban regions including Toronto, Beijing, and cities across Asia with offline methods,^43,49,57,90–94^ confirming that these compounds are common in urban atmospheres. I-CIMS detected 15 compounds in the gas-phase and 13 compounds in the particle phase, while LC-ESI Orbitrap MS detected 15 compounds in particle-phase filter samples (Figure 2). The mass defect plots (Figure 3a and 3b) show the basis for non-targeted identification of PFAS with I-CIMS. PFAS cluster near or below a mass defect of zero due to the negative mass defect of fluorine (−0.00144 Th per fluorine atom) which depresses the mass defect of perfluorinated compounds relative to other organics. This distinct mass defect signature allowed for the non-targeted identification of PFAS within the broader mass spectrum. The linear pattern of PFAS seen on the mass defect plot shows the systematic addition of CF_2_ units. Comparing gas-phase (Figure 3a) and particle-phase (Figure 3b) mass defect plots reflects the different compound distributions between phases, with higher mass and lower volatility compounds more abundant in the particle phase. Complementary identification was provided by the LC-ESI Orbitrap MS extracted ion chromatogram (Figure 3c), which shows the resolved chromatographic peaks for PFAS in the Houston particle-phase filter samples. Short-chain PFCAs eluted earliest and long-chain compounds and sulfonic acids eluted latest. Each peak was confirmed by compound-specific MS/MS fragmentation spectra matched against reference spectra from calibration (Figure S7), providing unambiguous structural identification.

**Figure 3:**
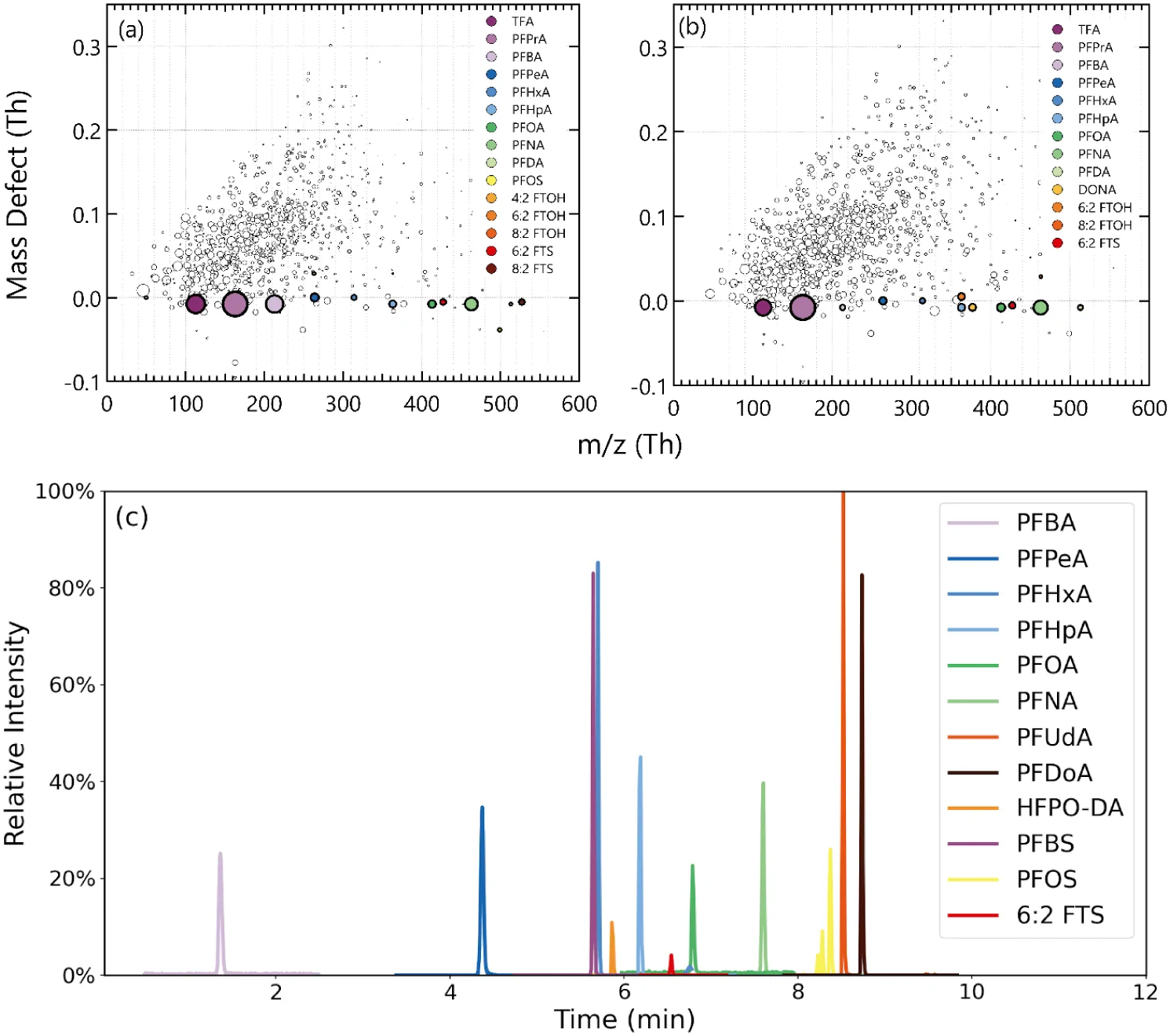
(a) Gas-phase and (b) particle-phase Kendrick mass defect of compounds detected by I-CIMS in Houston. Detected PFAS compounds are denoted by colored circles while all other organic compounds detected in Houston are white circles. PFAS compounds appeared at more negative mass defects compared to other organics. Circle size corresponds to signal strength. (c) Extracted ion chromatogram (EIC) of PFAS measured with the LC-ESI Orbitrap MS from the samples collected in Houston.

### 5.1 Gas-Phase PFAS

Gas-phase PFAS concentrations in Houston were high among urban regions, with a median gas-phase ΣPFAS concentration of 2.2 pptv (17.7 ng m^–3^, Table S13), this median concentration is higher than summertime gas-phase measurements with acetate CIMS in Toronto (3.2 ng m^–3^) at the same temperature and relative humidity,^49^ but only gas-phase short-chain PFCAs were measured in Toronto. Gas-phase PFAS concentrations followed similar time series during the measurement campaign (Figure S12), suggesting co-emission from common sources or the susceptibility to the same photochemical and meteorological processes. Gas-phase PFAS concentrations also exhibited a progressive decrease over the campaign duration, consistent with the seasonal transition from summer to autumn in Houston. This same decreasing trend was observed at this site for gas-phase oxygenated organic molecules during the same campaign and was attributed to declining ambient temperature and reduced photochemical activity,^70^ both of which would reduce gas-phase PFAS through decreased volatilization and reduced secondary PFCA production from FTOH oxidation.^26,95^ PFCAs were the most abundant PFAS class observed (ΣPFCA of 1.6 pptv, or 58% of total ΣPFAS). PFPrA and TFA were responsible for the majority of ΣPFCA, with median concentrations of 0.61 pptv and 0.48 pptv, respectively. Among PFCAs, concentration generally decreased with chain length (Figure S13), consistent with previous observations of PFCA concentrations in both urban and remote environments.^90,96–98^ This pattern may reflect the preferential emission of short-chain PFCAs or atmospheric production: successive “chain unzipping” PFCA oxidation reactions lead to the production of progressively shorter chain PFCAs.^1^ However, gas-phase PFPeA, PFPrA and PFOA deviated from this trend with elevated concentrations relative to adjacent chain lengths, suggesting additional direct emission sources from industry or consumer products.

FTOHs were the second most abundant PFAS class. The median daily gas-phase ΣFTOH (comprising 4:2, 6:2, and 8:2 FTOH) was 1.1 pptv (4960 pg m^–3^), about 70% of ΣPFCA (Figure S14). This contrasts with observations with LC-MS in Toronto that found FTOH accounted for up to 84% of total gas-phase ΣPFAS.^90^ The lower FTOH fraction likely reflects enhanced photochemical conversion of FTOH to PFCA during autumn in Houston, when solar fluxes remain high. Gas-phase ΣFTOH in Houston (thousands of pg m^3^) exceeded concentrations measured with LC-MS in Toronto,^90^ Hamburg,^99^ Manchester,^100^ and the urban Northeastern United States^101^ (hundreds of pg m^–3^), but was significantly lower than measurements of ΣFTOH exceeding 50,000 pg m^–3^ with LC-MS in urban South Korea.^102^ This wide range of FTOH concentrations in urban regions worldwide reflects regionally variable emissions, consistent with their relatively shorter atmospheric lifetimes compared to PFCAs or PFSAs.^103,104^

Correlations with collocated gas-phase species are consistent with a photochemical contribution to gas-phase PFAS profiles, although temperature-driven volatilization and photochemical production cannot be fully separated by correlation analysis alone. Gas-phase PFCA and PFSA concentrations were positively correlated with ozone throughout the measurement campaign (Figure S15) and corresponded with moderate easterly winds (Figure S16), which transport air masses from the Houston Ship Channel petrochemical complex and are associated with enhanced photochemical processing in Houston.^105^ Ambient temperature and ozone are tightly coupled in Houston, therefore the observed correlation of PFAS with ozone may reflect both enhanced PFAS volatilization at higher temperatures and photochemical production of PFCAs. However, the negative correlations between PFCAs and PFSAs and NO_*x*_ (−0.78 < r < −0.06) are not expected from temperature-driven volatilization and are consistent with previous findings that yields of PFCAs formed via FTOH photooxidation decrease with increasing NO_*x*_.^49,106,107^ OH oxidation of FTOH produces fluorinated peroxy radicals (RO_2_);^108^ under low-NO_*x*_ conditions, RO_2_ + HO_2_ pathways favor PFCA formation, while under high-NO_*x*_ conditions, RO_2_ + NO produces alkoxy radicals that fragment through pathways with reduced PFCA yields.^95,106^ Consistent with this mechanism, gas-phase FTOH in Houston correlated positively with NO_*x*_ (0.27 < r < 0.41), indicating FTOH accumulation under conditions unfavorable to PFCA formation. Gas-phase PFCAs also correlated positively with NH_3_ (Figure S15), which likely reflects their shared midday diurnal pattern driven by photochemistry, volatility, and boundary layer dynamics,^71^ rather than a direct chemical relationship. These correlations support a photochemical contribution from oxidation of fluorotelomer precursors to observed PFCA loadings in Houston.

### 5.2 Particle-Phase PFAS

Median concentrations, range of concentrations and detection frequencies of particle-phase PFAS are shown in Table S14. The total particle-phase PFAS mixing ratio was 0.25 pptv (3.1 ng m^–3^). Of the 15 PFAS detected in the gas-phase, 13 were also present in the particle-phase (Figure 2a). Notably, the absence of 8:2 FTS and PFOS from the particle-phase I-CIMS results was confirmed by targeted LC-ESI Orbitrap MS analysis (Figure 2b).^109,110^

PFCAs were the most abundant particle-phase PFAS class with a median daily average mixing ratio of 0.15 pptv (1.5 ng m^–3^). PFHpA, PFPrA, and PFPeA contributed most to particle-phase ΣPFCA, accounting for 0.02 pptv (0.3 ng m^–3^), 0.03 pptv (0.23 ng m^–3^), and 0.02 pptv (0.22 ng m^–3^), respectively. Particle-phase PFCA concentrations generally decreased with chain length, though the distribution was shifted more toward long-chain (C ≥ 8) PFCAS in the particle-phase as opposed to the gas-phase (Figure 2b). PFOA, PFNA and PFDA accounted for 9.1% of the observed particle-phase ΣPFCA (as opposed to only 2.3% in the gas-phase). The particle-phase PFOA fraction (5% of ΣPFCA) is consistent with observations in urban,^111^ rural,^112^ marine,^54^ and arctic^39^ regions. The enrichment of long-chain PFCAs in the particle-phase reflects their lower volatility and greater tendency to partition into aerosols, as discussed further in Section 5.3. DONA, an PFECA, was the only PFAS detected solely in the particle-phase. DONA has previously been detected with LC-MS in the ambient atmosphere in Japan^91^ and Albany, New York.^19^ DONA is associated with fluoropolymer manufacturing and is typically more abundant in indoor air.^5,19^ The presence of DONA in the particle-phase in the ambient atmosphere suggests that long-range transport or emissions from indoor sources may be significant influences on observed PFAS loadings in Houston.

FTOH represented 26% of the particle-phase ΣPFAS. The presence of FTOH in particles despite their relatively higher volatilities reflects the tendency of FTOH compounds to readily sorb onto particle surfaces even in dry conditions.^113,114^ Notably, 4:2 FTOH was not detected in the particle-phase at any point during the measurement campaign. This may be due to the high volatility of 4:2 FTOH favoring its partitioning out of the particle phase or off the FIGAERO filter before the desorption cycle initiated. The particle-phase abundance of FTOH may also be underestimated because of heterogeneous oxidation reactions in the particle phase that consume FTOH to produce PFCAs.^113^

Sulfur-containing PFAS (FTS and PFSAs, grouped here as ΣPFSA) were the least abundant compound class observed in Houston. Among PFSAs, only 6:2 FTS was detected above the LOD in the particle-phase. 6:2 FTS is frequently detected in both urban and remote atmospheres and its emissions can be traced to industrial processes such as electroplating, consumer products, landfill leachate, and aqueous film-forming foams in firefighting.^28,100,115,116^ Particle-phase 6:2 FTS was also unique among the observed PFAS in Houston because it had a strong association with southwesterly winds, which was not observed for any other compound (Figure S17). This suggests that particle-phase 6:2 FTS had a unique emission source in the Houston area that does not co-emit PFCA or FTOH compounds.

### 5.3 Gas-to-Particle Conversion

Gas-to-particle partitioning of PFAS is a critical factor in understanding the environmental fate and public health impact of atmospheric PFAS, and is fundamentally shaped by PFAS chemical structure. Replacement of hydrogen with fluorine on the carbon backbone reduces molecular polarizability and intermolecular dispersion forces, increasing volatility relative to analogous hydrogenated compounds.^1^ The functional group, however, substantially moderates this effect: PFCAs and PFSAs are strong acids that are fully deprotonated under environmental conditions and exist as nonvolatile anions in aqueous and aerosol phases, while FTOHs retain neutral character and higher gas-phase volatility.^117,118^ These structural factors produce orders-of-magnitude variation in volatility and aqueous solubility across the PFAS measured in Houston.

The observed average diurnal patterns of gas- and particle-phase PFAS concentrations revealed the environmental drivers of their gas-particle partitioning (Figure 4). Gas-phase PFCAs and PFSAs peaked in the afternoon (12:00–16:00) and correlated positively with ambient temperature (0.5 < r < 0.93), indicating that higher temperatures enhance volatilization into the gas-phase (Figure S15). Diurnal afternoon peaks of gas-phase PFAS in Houston are consistent with online measurements in Toronto and high time resolution (4 h) offline samples in Beijing.^49,50^ In contrast, diurnal patterns of particle-phase PFCAs and PFSAs correlated negatively with temperature (−0.57 < r < 0.039) and positively with relative humidity (0.017 < r < 0.491, Figure S18), consistent with partitioning of acidic and water-soluble PFAS into aqueous aerosol.^90,119,120^ Positive correlations between particle-phase PFAS and NO_*x*_ (0.13 < r < 0.7) likely reflect this same diurnal pattern, which both species accumulating overnight under shallow boundary layer conditions and high relative humidity. FTOH compounds did not display strong diurnal patterns, likely due to the higher volatility and relatively lower hygroscopicity of FTOH compounds resulting in less pronounced responses to variations in temperature and relative humidity, as described previously.^121,122^

**Figure 4:**
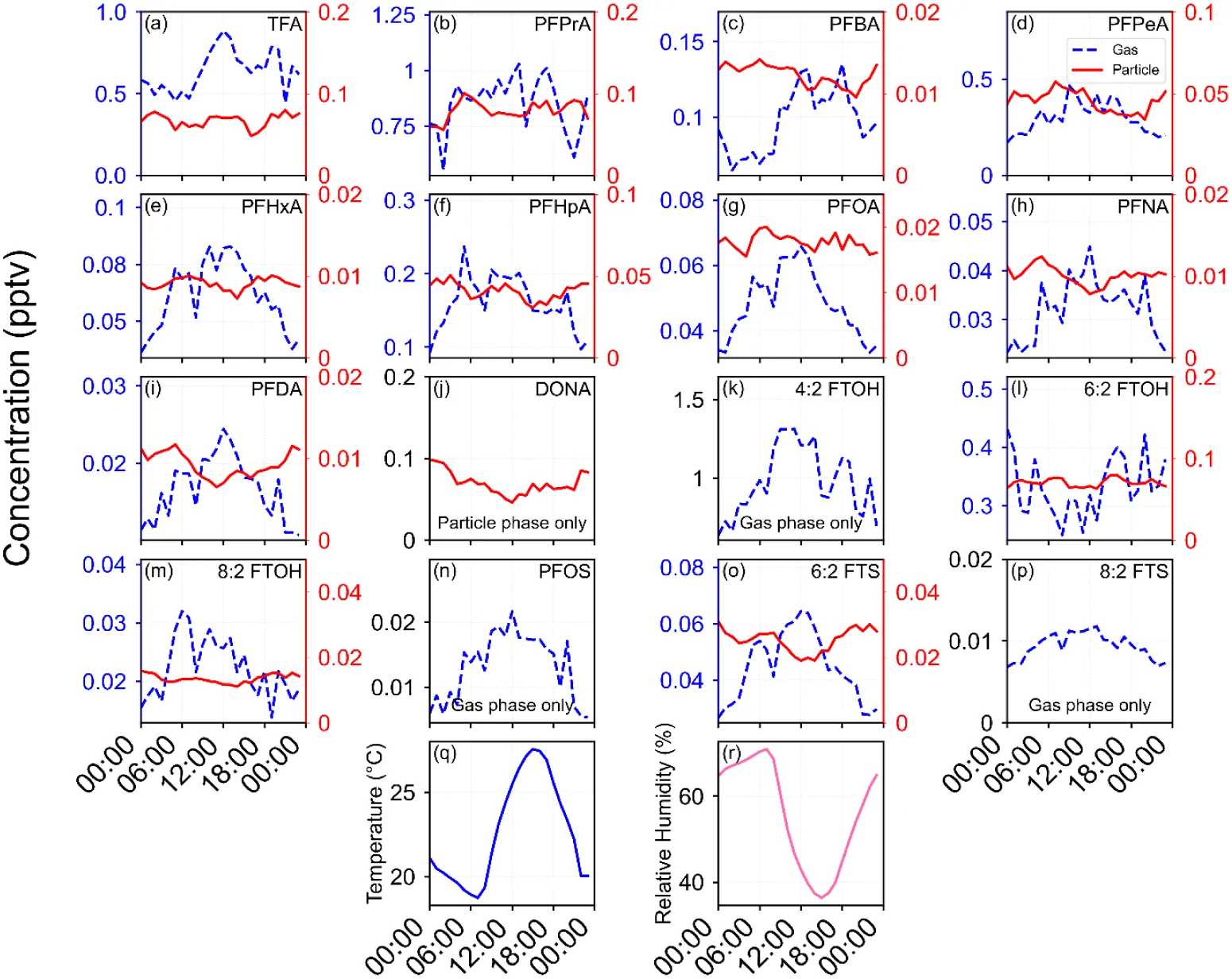
(a–r) Average diurnal cycles of gas-phase (blue dotted lines, left axis) and particle-phase (red solid lines, right axis) PFAS measured with the I-CIMS in Houston. Average (s) temperature and (t) relative humidity are also shown. (k) DONA was only detected in the particle phase, while (l) 4:2 FTOH, (p) PFOS, and (r) 8:2 FTS were only detected in the gas-phase.

The gas-particle partitioning coefficient (*K*_*p*_) and particle-associated fraction (Φ) provide a quantitative framework for interpreting these observations. *K*_*p*_ was calculated from the gas- and particle-phase FIGAERO data using eq 1:^123^

1 $$K_{p}=\frac{\left(c_{p}/TSP\right)}{c_{g}}$$

where *c*_*p*_ is the particle-phase PFAS concentration in ng m^–3^, *c*_*g*_ is the gas-phase PFAS concentration in ng m^–3^, and *TSP* is the mass concentration of particulate matter in μg m^–3^. Φ was calculated using eq 2:^90^

2 $$\Phi =\frac{c_{p}}{c_{g}+c_{p}}$$

These quantitative values of gas-particle partitioning behavior were compared with vapor pressure and subcooled liquid vapor pressure (*P*_*L*_) of PFAS (Figure 5). Vapor pressure and *P*_*L*_ were calculated for each compound using the U.S. EPA’s EPI Suite v4.11.^124^ For PFAS detected in both the gas- and particle-phase, log *K*_*p*_ ranged from −1.81 (PFPrA) to −0.97 (8:2 FTOH), similar to values reported for Toronto,^90^ Beijing,^58^ coastal China,^125^ rural China,^126^ and Arctic atmospheres.^125^ *K*_*p*_ decreased with increasing vapor pressure (Figure 5a), confirming that lower-volatility compounds partition more strongly into the particle phase and consistent with the enrichment of long-chain PFCAs in the particle-phase as described previously. The relationship between Φ and *P*_*L*_ followed a sigmoidal curve (Figure 5b), as previously observed for ambient PFAS^90^ and for other semivolatile organic compounds.^127^ However, the center of the sigmoidal relationship in Houston was shifted to the right by approximately two orders of magnitude compared to wintertime Toronto PFAS measurements.^90^ This rightward shift signifies enhanced partitioning into the particle-phase at a given vapor pressure, which can be attributed to the higher temperature and relative humidity in Houston compared to winter in Toronto and the strong hygroscopicity of PFAS. Under warm and humid conditions, aqueous aerosol or aerosol with a water film provide an efficient condensed-phase sink for water-soluble PFAS, which shifts the gas-particle equilibrium toward the particle-phase even for relatively volatile compounds. Further supporting this, indoor FTOH partition coefficients reported by Eichler et al. are approximately two orders of magnitude lower than our Houston values,^114^ which likely reflects the lower humidity and reduced aqueous aerosol contribution in indoor environments.^128^

**Figure 5:**
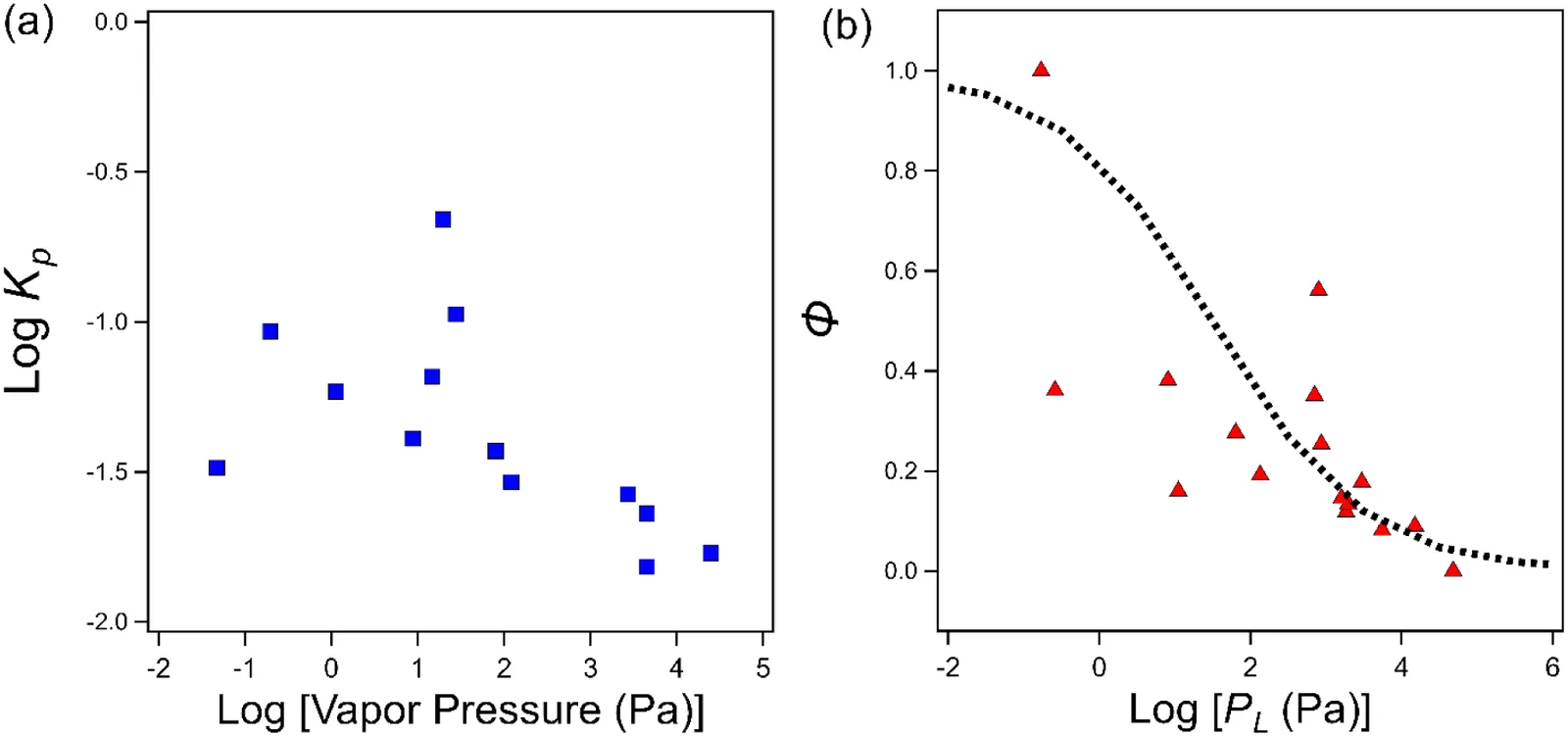
(a) The relationship between the gas-particle partitioning coefficient (*K_p_*) measured in Houston and PFAS vapor pressure. Lower vapor pressures favor higher gas-particle partitioning in PFAS. (b) Particle-associated fraction (*Φ*) of PFAS measured in Houston vs. subcooled liquid vapor pressure (*P_L_*, Pa) of PFAS compounds.

## 6 PFAS at Two Urban Sites

To investigate how different urban environments in the U.S. may influence atmospheric PFAS profiles, we compared targeted analysis of filter samples collected in Houston and Huntsville, Alabama. Huntsville is the most populated metropolitan area in Alabama with high military activity and nearby fluoropolymer production facilities. Eight PFAS compounds were detected in Huntsville: 6:2 FTS, PFHxA, PFHpA, PFOA, PFNA, PFDA, perfluoroundecanoic acid (PFUdA), and perfluorododecanoic acid (PFDoA) (Figure 6). The total concentration of PFAS in Huntsville ranged from 110 – 130 pg m^–3^ (Table S15), 5.5 times lower than the concentrations measured in Houston. PFCAs were the most abundant PFAS in Huntsville, contributing 98% of the total concentration compared to 45% in Houston. This comparison reveals that while PFCA abundance is consistent across U.S. urban environments, total concentrations and chemical profiles vary significantly depending on local sources.

**Figure 6:**
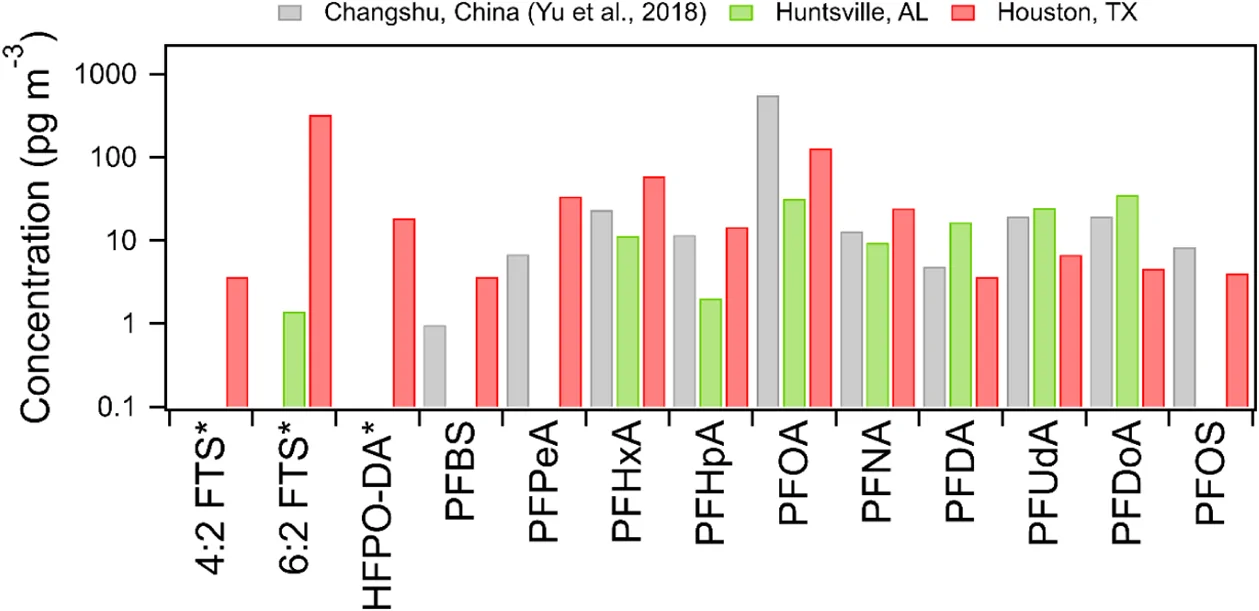
A comparison of mean particle-phase PFAS concentrations measured by LC-MS in Changshu, China,^57^ Huntsville, AL and Houston, TX. Starred compounds were not targeted for the measurements in Changshu, and thus the lack of data does not indicate absence of these compounds. In general, long-chain PFCA concentrations were similar between these three urban areas. Fluorotelomer sulfonic acids were abundant in Houston in contrast to Changshu and Huntsville, indicating a unique PFAS emission profile.

The two cities exhibited strikingly different PFCA chain-length distributions, reflecting their distinct emission landscapes. In Houston, PFCA concentrations decreased with increasing chain length, consistent with patterns observed in China^57^ and most other sites.^90,96–98^ However, Huntsville exhibited a surprising PFCA pattern where compounds increased with chain length, with long chain compounds (C8–C12) predominating. This pattern likely reflects Huntsville’s proximity to potential long-chain PFCA sources, including Redstone Arsenal to the south (where there are frequent rocket fuel burns that necessitate the use of legacy PFAS-containing aqueous film-forming foams) and fluoropolymer manufacturing facilities near Decatur, Alabama (∼25 km southwest) that have historically produced long-chain PFCAs.^129^ The persistence of legacy PFAS in the atmosphere from nearby fluoropolymer manufacturing despite phasing out of industrial production and use has also been noted in Osaka, Japan, where long-chain PFAS remain atmospherically relevant 10 years after industrial production ceased.^91^ The 6:2 FTS concentration in Huntsville (1–2 pg m^–3^) was two orders of magnitude lower than Houston (300 pg m^–3^), indicating that emission sources of fluorotelomer sulfonates prevalent in Houston are absent or minimal near Huntsville. These contrasting profiles demonstrate that urban PFAS profiles are highly variable based on distinct local emission sources.

PFOA concentrations at both U.S. sites were substantially lower than observations in Changshu, China in 2019, when PFOA was still in active production.^57^ However, in Houston and Huntsville, PFOA concentration was higher relative to other long-chain PFCAs. This elevated PFOA in the atmosphere of U.S. urban sites is consistent with observations in North Carolina that found elevated PFOA at a variety of urban, rural, and agricultural sites despite the phasing out of PFOA production over 20 years prior.^34^ Elevated PFOA in the urban U.S. atmosphere could be explained by atmospheric oxidation of fluorotelomer precursors, volatilization from contaminated soils or water, exchange with indoor air from buildings containing PFAS-treated materials and consumer products,^18,19^ or continued emissions from products manufactured before regulatory restrictions.^130^ As stated previously, PFOA was positively correlated with ozone in Houston (Section 5.1), which supports photochemical production as a potential source. The persistence of PFOA across geographically and industrially diverse U.S. sites as well as the previously noted persistence of long-chain PFCAs near historical point sources underscores that legacy PFAS contamination continues to influence urban atmospheric composition even as direct emissions decline.

The targeted analysis in Huntsville did not detect newer-generation PFAS included in the EPA Method 1633 target list, including GenX, DONA, PFECAs, or PFESAs.^56^ In contrast, GenX was detected at 18 pg m^–3^ in Houston. The absence of these replacement PFAS in Huntsville despite the nearby presence of active fluoropolymer manufacturing facilities may reflect differences in the effectiveness of modern emissions control technologies for newer versus legacy PFAS compounds,^131^ limitations of targeted methods in capturing the full range of PFAS currently in industrial use, or both. As regulations drive shifts toward these replacement chemical substances, non-targeted measurement approaches will be increasingly critical to accurately characterize PFAS in the atmosphere. These results underscore that comprehensive PFAS monitoring requires both targeted methods for quantifying known compounds and non-targeted techniques for identifying novel or unregulated PFAS not yet captured by standard target lists.

## 7 Limitations

Several methodological limitations apply to the findings of this study. The FIGAERO inlet of the I-CIMS included a 1 μm cyclone impactor, while particle-phase samples for offline LC-ESI Orbitrap MS analysis were collected with a 2.5 μm cyclone impactor. Both methods exclude coarse mode particles associated with significant PFAS reservoirs including sea-spray aerosol, resuspended soil and road dust,^132–134^ which means absolute concentrations reported here may underestimate total particle-phase PFAS loadings in the real atmosphere. However, the size-cut mismatch between the two instruments does not account for the discrepancy in particle-phase concentrations between I-CIMS and LC-ESI Orbitrap MS. The observed discrepancy is most plausibly attributable to analyte loss during filter sample collection and handling, including volatilization of PFAS from filters during sample collection, transport and storage; incomplete sample extraction, and losses during SPE workup. The quartz fiberglass filters used for offline LC-ESI Orbitrap MS aerosol sampling are also subject to gas-phase PFAS sorption artifacts during sample collection.^90,135,136^ As a result, absolute particle-phase concentrations measured by LC-MS may be biased relative to true suspended particulate PFAS. The cross-validation comparison between I-CIMS and LC-MS in Section 4 is based on relative composition and compound identification rather than absolute concentrations, and the gas-particle partitioning analysis in Section 5.3 relies exclusively on concurrent gas-phase and particle-phase I-CIMS measurements with the FIGAERO inlet, so the quartz fiberglass filter sorption artifact does not propagate into the partitioning conclusions presented here. The LC-MS analysis is also based on composite filter samples, which limits the temporal resolution of the offline measurements and prevents direct sub-daily comparison with the I-CIMS data.

The I-CIMS calibration introduces additional uncertainties. Iodide-adduct formation can show humidity- and temperature-dependent sensitivity, particularly for compounds quantified through the proton-transfer pathway.^61,74,82,83^ We did not perform systematic RH- or temperature-dependent calibrations across all compound classes. Additionally, the I-CIMS background signal during calibration was elevated for some PFAS, including TFA, PFPrA, and PFBA (Figure S9) possibly due to fluorinated artifacts from PTFE components in the FIGAERO inlet and sampling lines, which we characterized through regular gas- and particle-phase background measurements (Section S1.1) and were subtracted from all reported concentrations. Although the background-subtracted ambient signals are above the dynamic LOD on a campaign-averaged basis, sub-hourly resolved measurements during periods of low ambient signal approached the detection threshold, introducing higher relative uncertainty in time-resolved interpretation at diurnal minima.

The correlation analysis between PFAS and environmental variables cannot fully deconvolute photochemical mechanisms from meteorological covariation, because ambient temperature, ozone, NO_*x*_, and boundary layer dynamics are tightly coupled. Although the NO_*x*_-dependent patterns of FTOHs and PFCAs are consistent with established laboratory mechanisms for FTOH photooxidation, the relative magnitudes of photochemical and meteorological contributions to observed loadings cannot be quantitatively determined from these observations alone.

The two-site comparison between Houston and Huntsville identifies clear differences in PFAS profiles attributable to distinct local emissions. However, the two measurements were conducted approximately 3 years apart and during different seasons (October 2022 in Houston and June-July 2025 in Huntsville), and contributions to PFAS loadings from temporal trends or seasonal differences cannot be fully separated from the geographic comparison. The dominant differences in PFCA chain-length distributions are most plausibly attributable to local emission sources rather than temporal or seasonal effects, but broader conclusions about U.S. urban PFAS variability require expanded measurements across additional cities, seasons and years.

## 8 Conclusions and Implications

We measured ambient atmospheric PFAS in Houston with iodide CIMS validated by concurrent offline LC-ESI Orbitrap MS based on EPA Method 1633. Both techniques captured consistent relative abundance patterns despite differences in absolute concentrations ranging from a factor of 2 to 8, supporting I-CIMS as a reliable approach for real-time PFAS monitoring once compound-specific calibration is performed. Calibration factors varied substantially among compounds and across instruments with similar IMR and inlet configurations, indicating that literature sensitivity values cannot substitute for instrument-specific calibration and motivating future intercomparison efforts across the CIMS community. Pairing online I-CIMS with annular denuder or sorbent tube sample collection for gas-phase LC-ESI Orbitrap analysis would extend cross-method validation to both gas- and particle-phases, and applying suspect screening or non-targeted workflows to LC-ESI Orbitrap MS/MS data would expand offline compound coverage beyond the EPA Method 1633 target list.

Consistent with prior urban observations from Toronto and Beijing, short-chain PFCAs dominated gas-phase loadings in Houston, gas-phase PFAS correlated with temperature and ozone, and particle-phase PFAS correlated with humidity.^49,50,58,90^ The particle-associated fraction as a function of subcooled liquid vapor pressure in Houston was shifted by approximately two orders of magnitude relative to wintertime Toronto and a further two orders relative to indoor environments.^90,114^ This more efficient uptake of PFAS into aqueous aerosol under humid conditions implies that sub-tropical urban regions may experience disproportionately high particle-phase PFAS burdens, which has direct consequences for inhalation exposure and wet deposition to surface waters. The inversion of PFCA chain-length distribution between Houston and Huntsville further demonstrates that local industrial emissions, particularly fluoropolymer manufacturing, can override regional and seasonal trends in shaping urban atmospheric PFAS loadings. The persistence of atmospheric PFOA at two distinct urban sites years after domestic production ceased highlights that legacy PFAS contamination remains atmospherically relevant through continuing secondary sources including atmospheric oxidation of fluorotelomer precursors, volatilization from contaminated soil or water, or emissions from pre-regulation consumer and industrial products. As atmospheric PFAS emissions evolve with regulatory and technological changes, atmospheric PFAS composition will evolve in ways that require sustained monitoring across diverse environments to understand the extent of human exposure.

## Supplementary Material



## Data Availability

The data used in the figures is available at https://zenodo.org/records/20631442.

## Notes

Text S1: additional I-CIMS methods information; Text S2: additional LC-ESI Orbitrap MS methods information; Text S3: additional measurement platform instrumentation; Figures S1–S19; Tables S1–S18 (PDF)
