# Supplementary material for: PFAS in the Urban Atmosphere Detected with I‑CIMS and LC-ESI Orbitrap Mass Spectrometer
Source: ACS EST Air. 2026 Sep 9;3(10):2811–26. doi: 10.1021/acsestair.6c00191 (PMC13660390; doi:10.1021/acsestair.6c00191)
Supplement: Supplementary file 1 [file ea6c00191_si_001.pdf]

Supplemental information for

PFAS in the Urban Atmosphere Detected with I-CIMS and LC-ESI Orbitrap Mass Spectrometer

Lee Tiszenkel[1], Vignesh Vasudevan-Geetha[1], Zachary Watson[1], James Flynn[2], and Shan-Hu Lee[1]*

[1] Dept of Atmospheric Science, University of Alabama at Huntsville, Huntsville, Alabama

[2] Department of Earth and Atmospheric Sciences, University of Houston, Houston, Texas

*Corresponding authors. Email: lt0021@uah.edu, shanhu.lee@uah.edu

Supplemental Information contains:

Supplementary Text S1-S3
Supplementary Figures S1-S18
Supplementary Tables S1-S18
Supplementary References

Text S1. Additional I-CIMS Methods Information

1.1. I-CIMS Inlet and Background Measurements

To minimize sampling losses, the air sampling inlet for the HR-ToF CIMS was made with a "near-isokinetic" inlet approximately 3 m above ground level[1-3]. Ambient airflow was taken at 80 slpm into a 50 cm stainless steel tube with an inner diameter of 8 cm. The gas- and particle-phase inlets of the HR-ToF CIMS were connected to the end of this intake. The gas-phase inlet was a 0.5 m long ¼" polytetrafluoroethylene (PTFE) tube through which 2.2 slpm was drawn into the gas-phase FIGAERO port. The particle-phase inlet was a 25 cm long ½" stainless steel tube through which 5 slpm was drawn through a 1-µm-pore PTFE filter in the FIGAERO inlet. The particle-phase inlet was fitted with a cyclone impactor (Mesa Labs SCC1.197) to remove particulate matter greater than 1µm in diameter. Particles were collected for 40 minutes on the FIGAERO filter and then subjected to a 2.5 slpm flow of heated nitrogen with a temperature ramp from ambient temperature to 200 °C over 10 minutes followed by an additional 10 min of heat soaking at a constant 200 °C. Gas-phase backgrounds were taken for 10 minutes of every hour of ambient monitoring by opening a programmatically controlled solenoid valve that displaced the 2.2 slpm gas-phase inlet flow with a 3 slpm overblow of pure N_2 from the headspace of a liquid N_2 dewar into a stainless-steel union tee connected to the gas-phase inlet. This resulted in a 10-minute background measurement preceding each 25-minute ambient gas-phase measurement. Particle-phase backgrounds were taken every 7 hours by directing the inlet flow through an upstream borosilicate glass filter (Balston 9933-11-BQ) before the FIGAERO filter during gas-phase measurements. The filtered air was captured on to the FIGAERO filter and subjected to a thermal desorption cycle measurement, which was taken as the particle-phase background. Figure 1 shows the differences between particle-phase backgrounds and measurement cycles for each compound,

comparisons between gas- and particle-phase backgrounds, and comparisons between gas- and particle-phase LODs. The first 2 minutes of each background and ambient gas-phase measurement were excluded from the data to account for the instrument e-folding time of 2 min.

1.2. Ozone-dependent I-Adduct Formation

Measurements of acids with I-CIMS can be subject to variability in the iodide adduct (HAI^-) and conjugate base (A^-) signals due to ambient conditions[4, 5]. Ambient measurements of TFA with I-CIMS in Toronto have shown a highly variable correlation between the iodide adduct TFA signal (m/z = 240.897, $C_2F_3O_2HI^-$) and the conjugate base signal (m/z = 112.985, $C_2F_3O_2^-$)[6]. This variability suggests that measurement conditions including ambient ozone and relative humidity affected the ionization due to formation of iodine oxides in the CIMS inlet that react with acids to form their conjugate base. During observations in Houston, the correlation between the conjugate base and iodide adduct is strong, especially at higher signals (Figure S19). Lower IO^- signals tended to bias the TFA adduct formation and higher IO^- signals biased the conjugate base form, as expected. Because the relative abundance of the conjugate base and I-adduct varies with ambient conditions, calibration factors and reported concentrations were derived from the dominant ion form for each compound (Table S3). The selected ion signal was normalized to the sum of the primary reagent ions (I^-, IH_2O^-, and IO^-) scaled to 10^6 counts per second. The same dominant ion form was used consistently in both calibration and ambient quantification. A lack of detection of the I-adduct was due to either many additional peaks at the m/z of the I-adduct, or the I-adduct being above m/z 600, where the sensitivity of the instrument was significantly lower. High resolution peak fits for the 18 PFAS detected in this study with I-CIMS are shown in Figure S2.

1.3. I-CIMS Calibration Error Analysis

A full desorption cycle with only pure methanol deposited on the filter was run between each calibration step to ensure full desorption of PFAS from the filter, and additional desorption cycles were run in case the signal during these blank steps was higher than the background. The ion signal was integrated over the course of the 10-minute desorption cycle to obtain a calibration signal in counts per second. For compounds detected as both the I-adduct and the conjugate base, the calibration signal was taken from the dominant ion form (Table S3).

Error in calibration was determined through least squares linear regression of the calibration curve. The residual standard error was calculated as the square root of the sum of squared residuals divided by the degrees of freedom. Standard errors in the slope of each calibration curve were calculated as the residual standard error divided by the square root of the sum of squared deviations from the mean. 95% confidence intervals for calibration parameters were calculated using a Student's t-distribution with n-2 degrees of freedom. Error in concentrations calculated from calibration curves was calculated according to the uncertainty from the calibration curves and uncertainty that increases with distance from the mean calibration signal according to:

$$s_x = \frac{s_y}{|b|} \cdot \sqrt{\frac{1}{m} + \frac{1}{n} + \frac{(y_0 - \bar{y})^2}{b^2 \sum (x_i - \bar{x})^2}} \quad (1)$$

Where s_x is the error of the calculated concentration, s_y is the residual standard error of the calibration curve, b is the slope of the calibration curve, m is the number of replicate measurements, n is the number of calibration points, y_0 is the measured signal, $\bar{y}$ is the mean signal of all calibration points, x_i is the concentration for calibration point i, and $\bar{x}$ is the mean concentration of all calibration points.

I-CIMS sensitivities can be susceptible to changes in relative humidity[7, 8]. RH-dependent biases vary across compounds and can act in either direction[9]. Our calibration for PFAS was performed under low-humidity conditions (RH <5%), and the resulting calibration factors may not fully

represent ambient sensitivities; however, the magnitude and direction of any such bias for PFAS cannot be definitively assessed from the present data. Mean errors in compound concentrations calculated by these calibration curves were between 10 and 95%. These errors are typical of errors in CIMS PFAS concentrations previously reported which are on the order of 10 - 150%[6, 10-12].

The calibration curves for TFA, PFPrA, PFBA, and PFNA (Figure S9) exhibited elevated intercepts compared to other PFAS calibration curves, which indicates non-zero background signals during the FIGAERO desorption cycles for these compounds. PFCAs are pervasive low-level contaminants in indoor air, solvents, and PFAS-handling materials and are therefore susceptible to background contributions during calibration. Possible sources include trace PFCA contamination of the PTFE filter substrate, incomplete desorption between calibration steps despite intermediate methanol blank cycles, and adsorption from laboratory air during filter handling. The dominant source cannot be identified from the calibration data alone. Because calibration factors are derived from the slope of the linear regression rather than the intercept, these backgrounds do not bias the derived sensitivities, and the dynamic background subtraction and LOD described in Section 2.2.1 accounts for residual backgrounds at each ambient measurement period even as backgrounds change over the course of the measurement campaign.

Text S2. Additional LC-ESI Orbitrap MS Methods Description

A schematic of the full SPE workflow is shown in Figure S5. Each filter sample and blank sample were spiked with 2 ng of extracted internal standard (EIS) (Table S5) and extracted three times with 10 mL, 15 mL and 5 mL 0.3% methanolic ammonium hydroxide in a polypropylene centrifuge tube placed in an ultrasonic bath for 20 minutes each cycle. The extracts of each sample were combined in a separate polypropylene centrifuge tube and 5 mL water added to make a total volume of 35 mL. The volume of the extract was then reduced to ~5 mL by evaporation under a

gentle stream of nitrogen. Before the cleanup step, the concentrated extracts were diluted with 40 – 45 mL of water and the pH adjusted to 6 - 6.5 with 5% formic acid solution or 3% aqueous ammonium hydroxide solution. Waters WAX/GCB cartridges (Milford, MA, USA) were used for the solid phase extraction (SPE) and carbon cleanup steps to remove interferences. The cartridge was loosely packed with clean silanized glass wool (Supelco, Bellfonate, USA) to half of its height and then connected to the reservoir via a SPE adapter and a nylon syringe filter. The cartridge was then subjected to pre-conditioning with 15 mL 1% methanolic ammonium hydroxide and 5 mL 0.3 M formic acid solution. The SPE was then loaded with the sample, and the eluate was discarded. The loaded SPE cartridge was rinsed with 10 mL water and 5 mL 1:1 0.1 M formic acid in methanol. After drying, the analytes were eluted from the SPE into a polypropylene tube using 5 mL 1% methanolic ammonium hydroxide. 2 ng of NIS (non-extracted internal standard) and 25 µL acetic acid was added to the sample. The 5 mL sample aliquot was then evaporated to ~250 uL under a gentle stream of nitrogen and then reconstituted to 750 µL after rinsing the walls of the polypropylene tubes with solvents having similar composition as the final sample (methanol with 4% water, 1% ammonium hydroxide and 0.0625% acetic acid). 300 µL from the final sample aliquot was transferred to a 500 µL polypropylene vial for further instrumental analysis.

A delay column (Thermoscientific Hypersil GOLD^{TM} C18 reverse phase, 3x 50 mm, 1.9 µm particle size) was installed between the binary pump and the autosampler to shift the retention time of any residual PFAS in the LC system. Chromatographic separation of analytes was achieved using C-18 reverse phase column (2.1 x 50 mm, 1.5 µm particle size, Thermoscientific Accucore) kept at 40°C. The mobile phase used were 2 mM ammonium actetate in 95:5 water : acetonitrile (A) and acetonitrile (B) in a 12-minute gradient elution program with 2-minute post-run equilibration (Table S16). The Orbitrap mass spectrometer was operated in negative ion mode

with parallel reaction monitoring (PRM) scan for high sensitivity quantification[13]. The optimized MS settings, collision energy and ion parameters for targeted analytes in PRM analysis is detailed in Table S17 and S18. Collision energy optimization of each analyte was achieved by maximizing the parent ion fragmentation with minimal daughter ion fragmentation.

Text S3. Key Trace Gas & Meteorology Measurements at the Houston Site

Measurement of temperature and relative humidity were taken with a Vaisala HMP-45c probe. Wind speed and direction were measured with an RM Young 05305 wind monitor. Ozone, NOx and SO_2 were measured with Thermo Scientific 49c, 42c, and 43i, respectively. Ammonia was measured with an ethanol CIMS[14-16]. Particle size distributions from 1 to 1000 nm were collected with a Particle Size Magnifier (PSM, Airmodus A10), and two scanning mobility particle sizers (SMPS) consisting of a CPC (TSI 3772, 3776), electrostatic classifier (TSI 3080), and a differential mobility analyzer (TSI 3081, 3085). Meteorological data, ozone, NOx, SO_2 and ammonia measurements were collocated in the measurement platform.

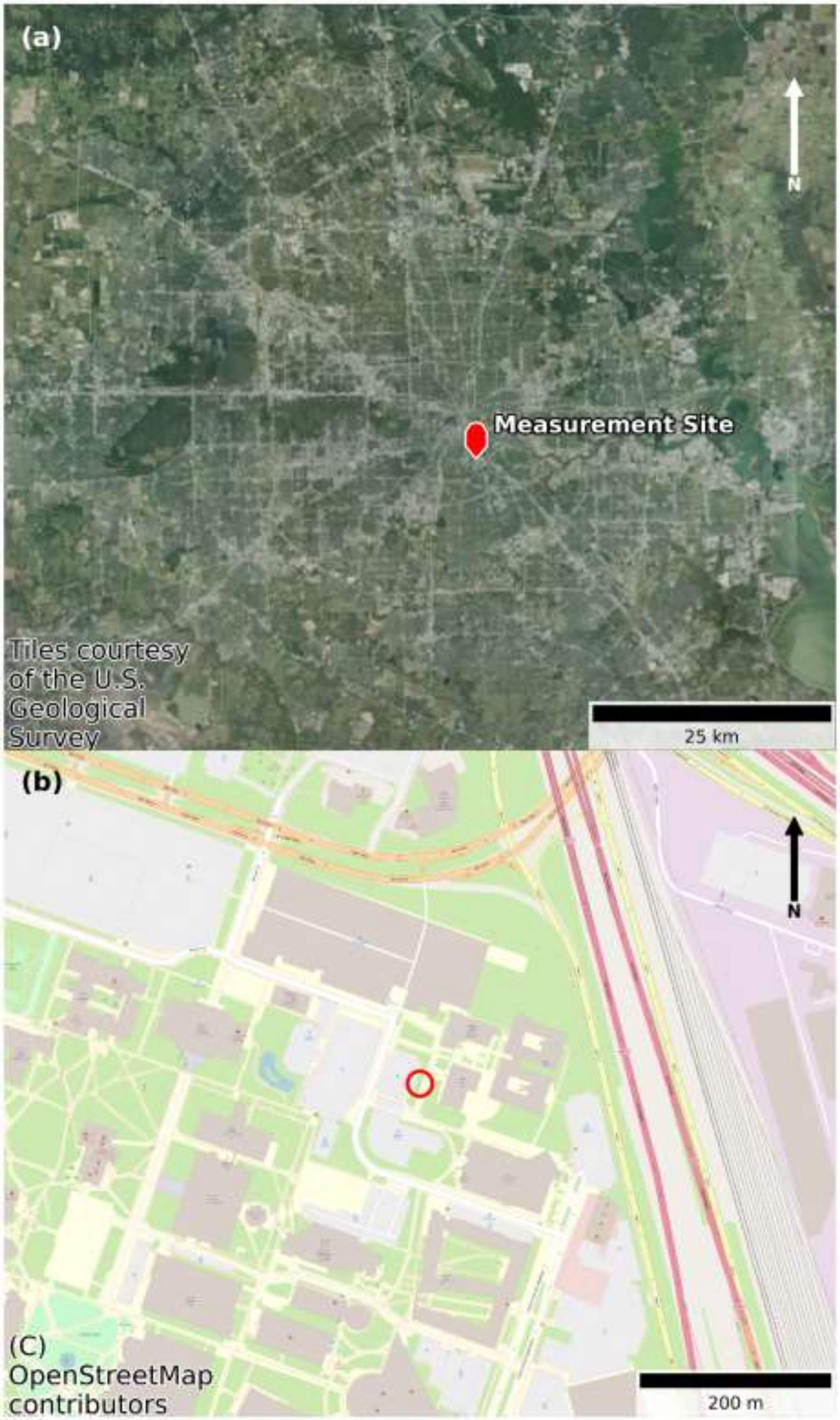

Figure S1. Map of the measurement area (a) indicated by the red marker in the greater Houston area, and (b) indicated by the red circle in the immediate vicinity.

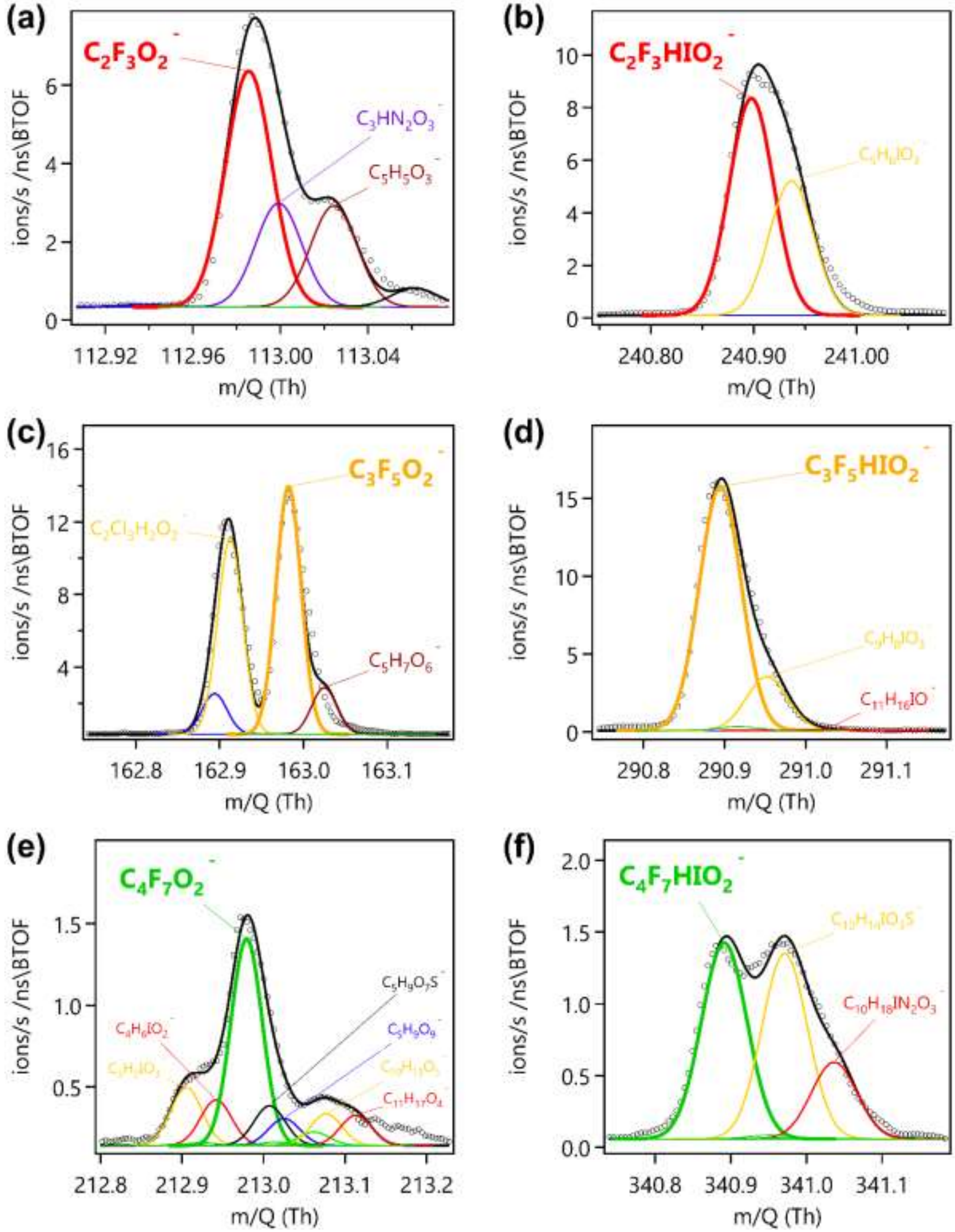
(a)
C2F3O2-
C3HN2O3-
C5H5O3-
ions/s /ns\BTOF
m/Q (Th)
(b)
C2F3HIO2-
ions/s /ns\BTOF
m/Q (Th)
(c)
C3F5O2-
C2Cl3H2O2-
C5H7O6-
ions/s /ns\BTOF
m/Q (Th)
(d)
C3F5HIO2-
C11H16IO-
ions/s /ns\BTOF
m/Q (Th)
(e)
C4F7O2-
C5H9O7S-
C4H6IO2-
C5H9O9-
C11H17O4-
ions/s /ns\BTOF
m/Q (Th)
(f)
C4F7HIO2-
C10H18IN2O3-
ions/s /ns\BTOF
m/Q (Th)

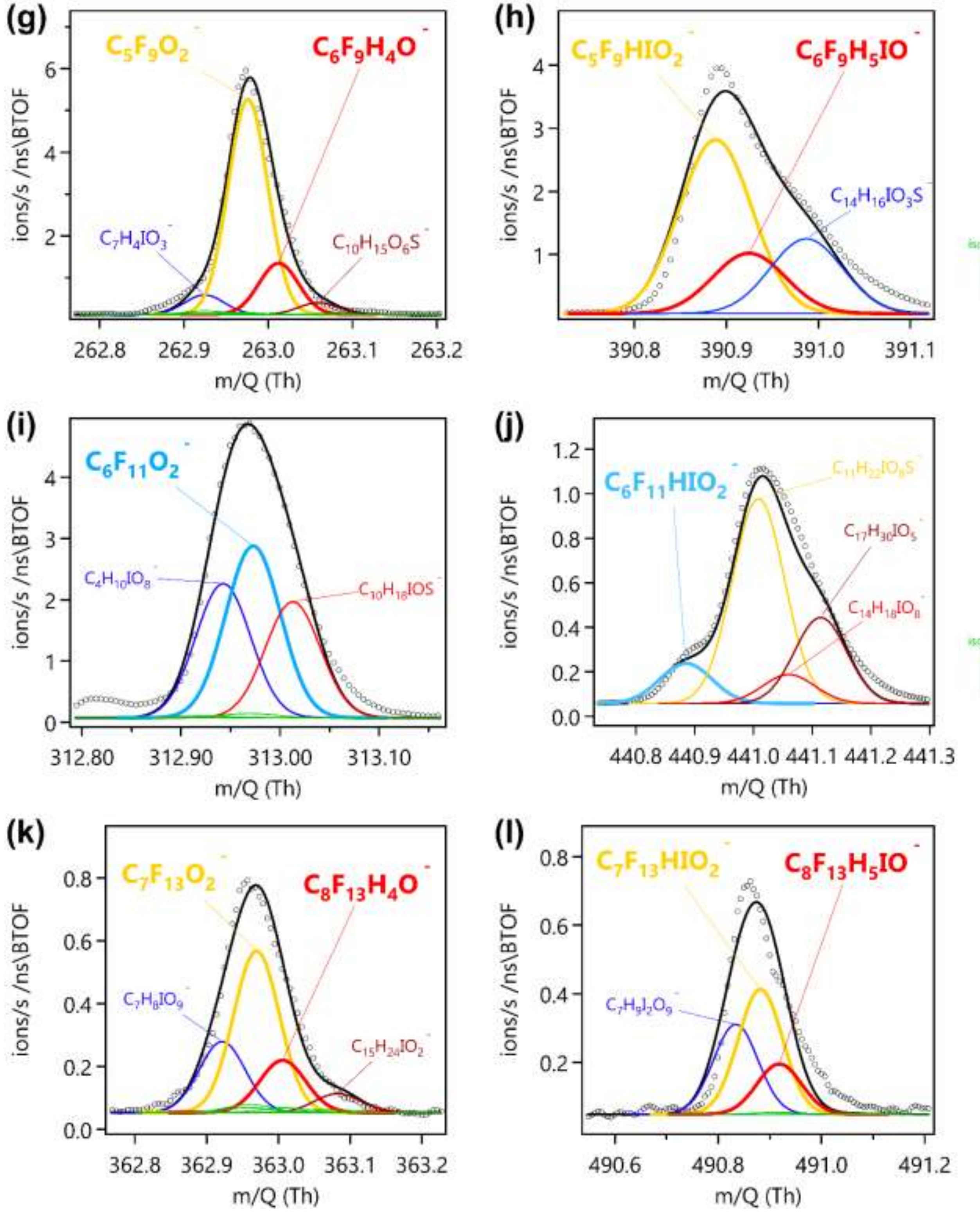
(g)
$C_5F_9O_2^-$
$C_6F_9H_4O^-$
$C_7H_4IO_3^-$
$C_{10}H_{15}O_6S^-$
ions/s /ns\BTOF
m/Q (Th)
(h)
$C_5F_9HIO_2^-$
$C_6F_9H_5IO^-$
$C_{14}H_{16}IO_3S^-$
ions/s /ns\BTOF
m/Q (Th)
(i)
$C_6F_{11}O_2^-$
$C_4H_{10}IO_8^-$
$C_{10}H_{18}IOS^-$
ions/s /ns\BTOF
m/Q (Th)
(j)
$C_6F_{11}HIO_2^-$
$C_{17}H_{30}IO_5^-$
$C_{14}H_{18}IO_8^-$
ions/s /ns\BTOF
m/Q (Th)
(k)
$C_7F_{13}O_2^-$
$C_8F_{13}H_4O^-$
$C_7H_8IO_9^-$
$C_{15}H_{24}IO_2^-$
ions/s /ns\BTOF
m/Q (Th)
(l)
$C_7F_{13}HIO_2^-$
$C_8F_{13}H_5IO^-$
$C_7H_9I_2O_9^-$
ions/s /ns\BTOF
m/Q (Th)

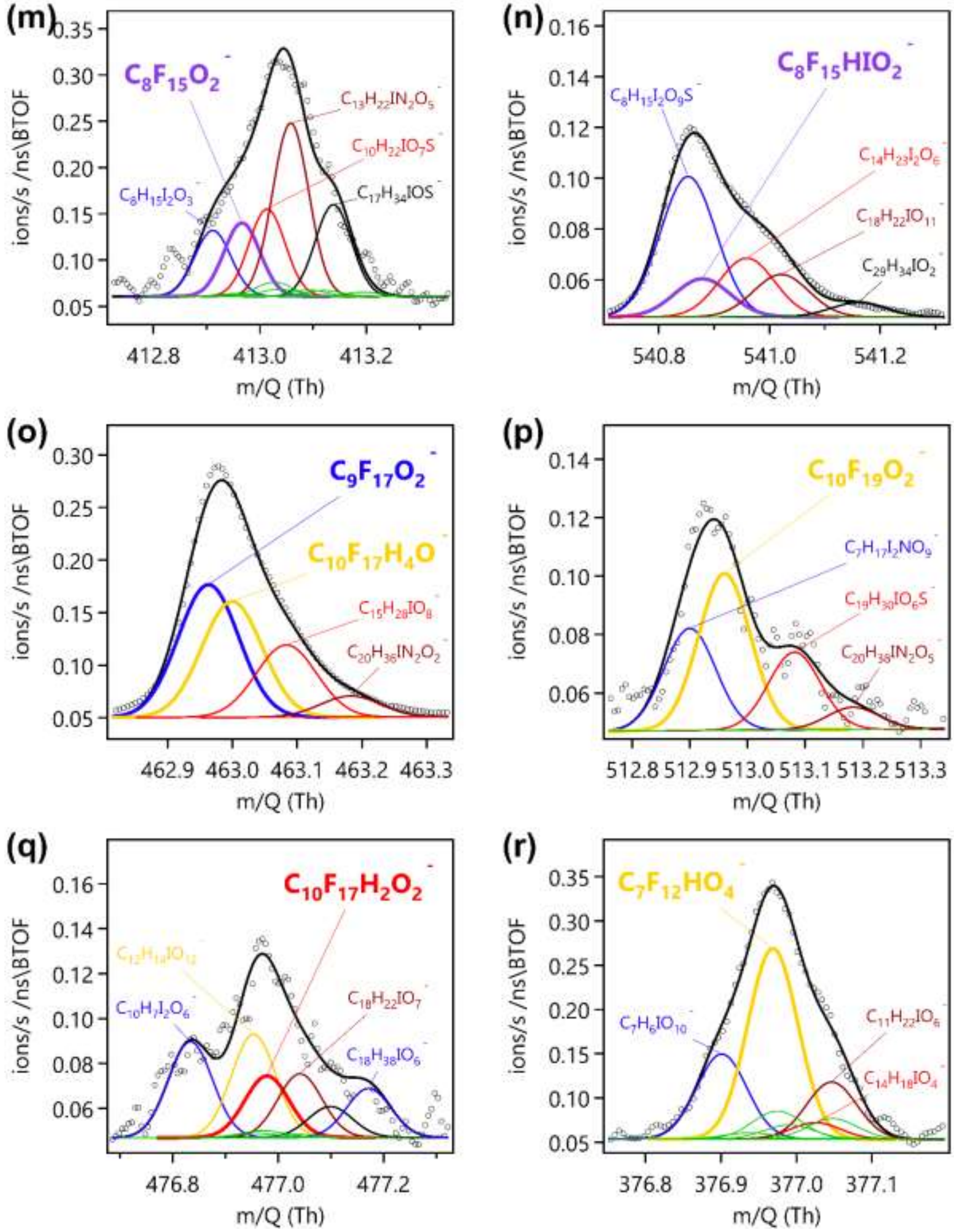

(m)
$C_8F_{15}O_2^-$
$C_{13}H_{22}IN_2O_5^-$
$C_{10}H_{22}IO_7S^-$
$C_8H_{15}I_2O_3^-$
$C_{17}H_{34}IOS^-$
(n)
$C_8F_{15}HIO_2^-$
$C_8H_{15}I_2O_9S^-$
$C_{14}H_{23}I_2O_6^-$
$C_{18}H_{22}IO_{11}^-$
$C_{29}H_{34}IO_2^-$
(o)
$C_9F_{17}O_2^-$
$C_{10}F_{17}H_4O^-$
$C_{15}H_{28}IO_8^-$
$C_{20}H_{36}IN_2O_2^-$
(p)
$C_{10}F_{19}O_2^-$
$C_7H_{17}I_2NO_9^-$
$C_{19}H_{30}IO_6S^-$
$C_{20}H_{38}IN_2O_5^-$
(q)
$C_{10}F_{17}H_2O_2^-$
$C_{12}H_{18}IO_{12}^-$
$C_{10}H_7I_2O_6^-$
$C_{18}H_{22}IO_7^-$
$C_{18}H_{38}IO_6^-$
(r)
$C_7F_{12}HO_4^-$
$C_7H_6IO_{10}^-$
$C_{11}H_{22}IO_6^-$
$C_{14}H_{18}IO_4^-$
ions/s /ns\BTOF
m/Q (Th)

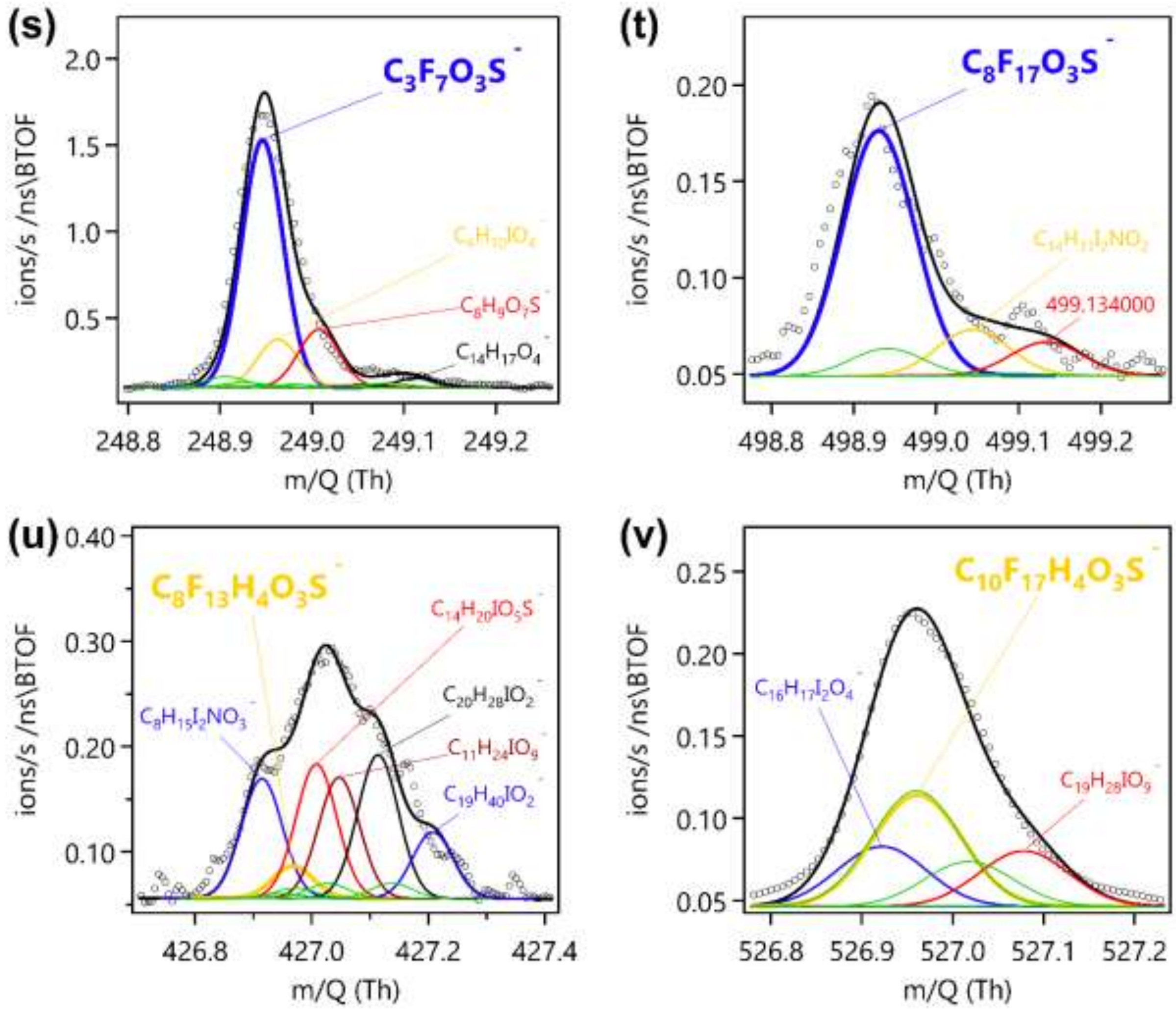

Figure S2. High-resolution MS fits for (a,b) TFA, (c,d) PFPrA, (e,f) PFBA, (g,h) PFPeA (yellow solid line) and 4:2 FTOH (red solid line), (i, j) PFHxA, (k, l) PFHpA (yellow solid line) and 6:2 FTOH (red solid line), (m, n) PFOA, (o) PFNA (blue solid line) and 8:2 FTOH (yellow solid line), (p) PFDA, (q) 8:2 FTAC, (r) DONA, (s) PFPrS, (t) PFOS, (u) 6:2 FTS, (v) 8:2 FTS. PFNA, PFDA, 8:2 FTAC, DONA, 8:2 FTOH, PFPrS, PFOS, 6:2 FTS and 8:2 FTS were not detected in their I-adduct forms.

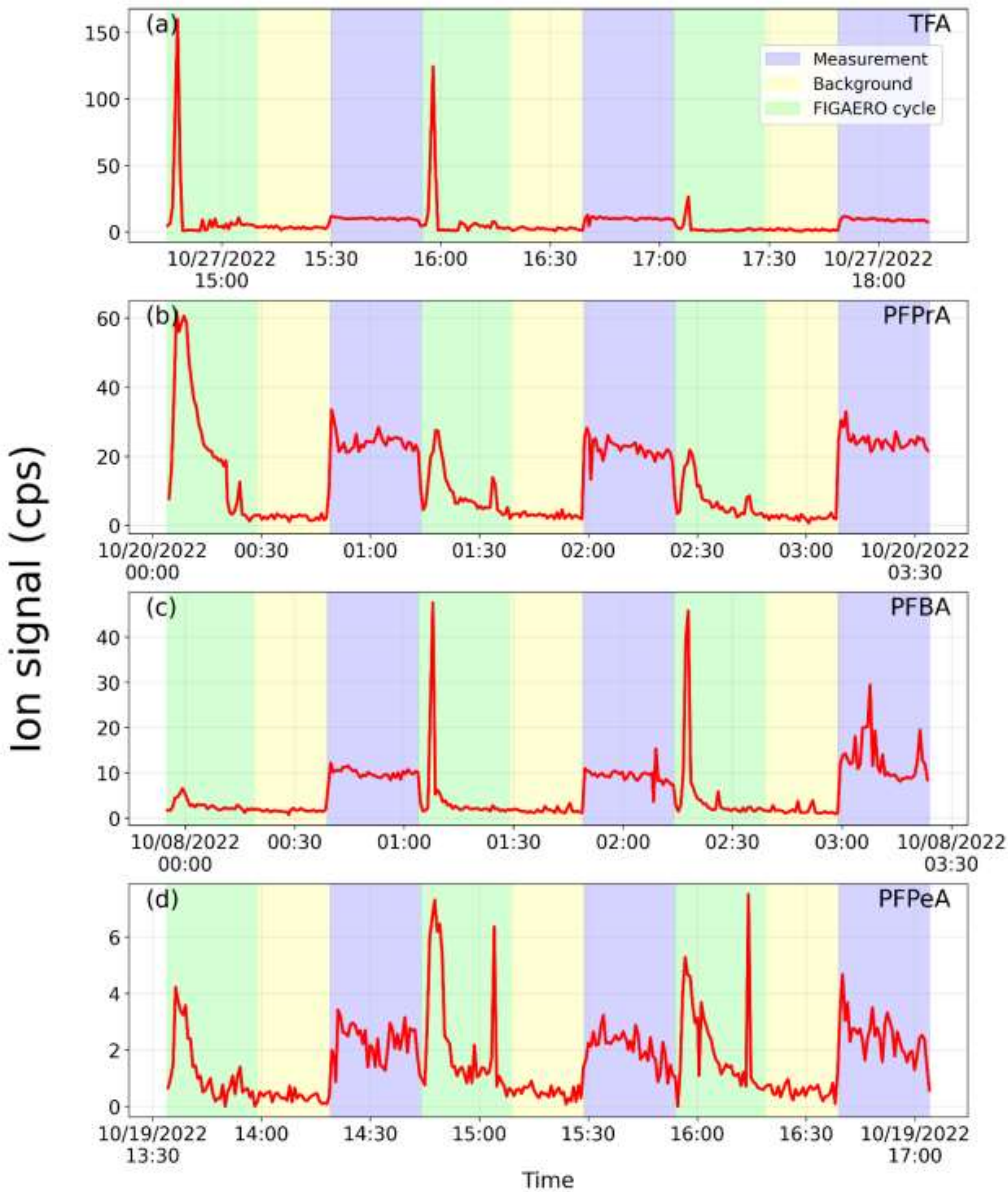
(a)
TFA
Measurement
Background
FIGAERO cycle
150
100
50
0
10/27/2022 15:00
15:30
16:00
16:30
17:00
17:30
10/27/2022 18:00
(b)
PFPrA
60
40
20
0
10/20/2022 00:00
00:30
01:00
01:30
02:00
02:30
03:00
10/20/2022 03:30
(c)
PFBA
40
30
20
10
0
10/08/2022 00:00
00:30
01:00
01:30
02:00
02:30
03:00
10/08/2022 03:30
(d)
PFPeA
6
4
2
0
10/19/2022 13:30
14:00
14:30
15:00
15:30
16:00
16:30
10/19/2022 17:00
Ion signal (cps)
Time

161

162

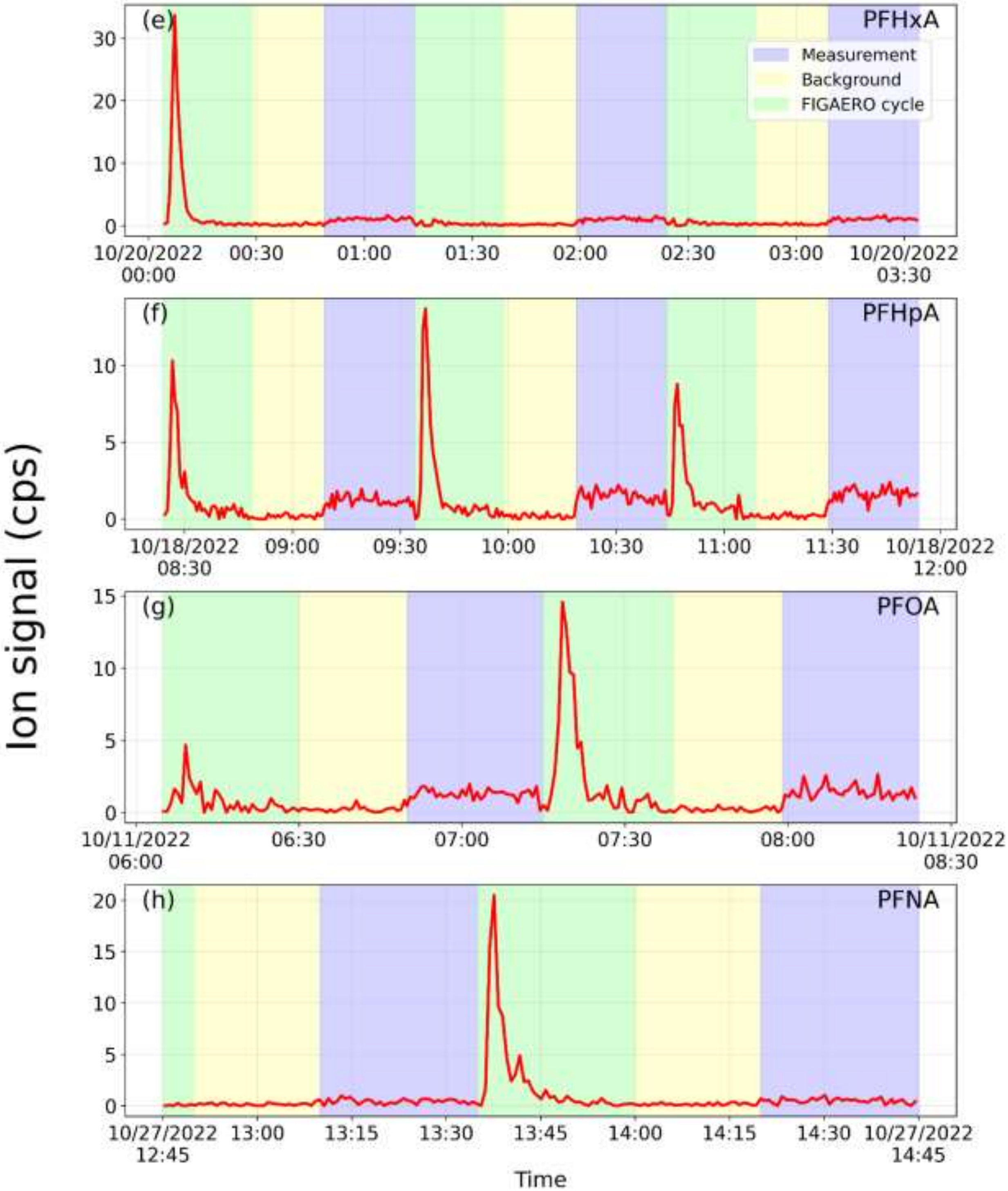

Ion signal (cps)
(e)
PFHxA
Measurement
Background
FIGAERO cycle
10/20/2022 00:00
00:30
01:00
01:30
02:00
02:30
03:00
10/20/2022 03:30
(f)
PFHpA
10/18/2022 08:30
09:00
09:30
10:00
10:30
11:00
11:30
10/18/2022 12:00
(g)
PFOA
10/11/2022 06:00
06:30
07:00
07:30
08:00
10/11/2022 08:30
(h)
PFNA
10/27/2022 12:45
13:00
13:15
13:30
13:45
14:00
14:15
14:30
10/27/2022 14:45
Time

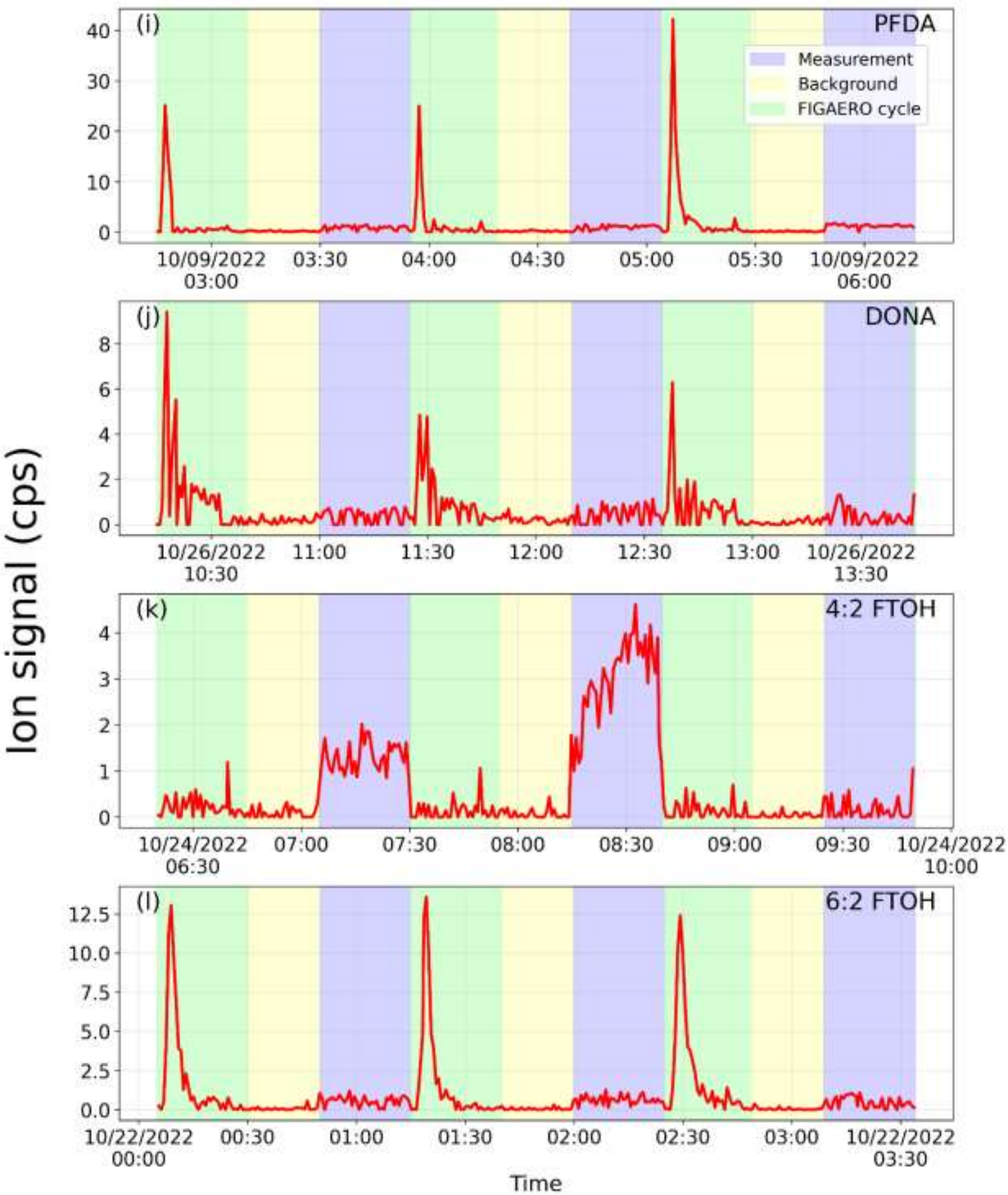

(i)
PFDA
Measurement
Background
FIGAERO cycle
(j)
DONA
(k)
4:2 FTOH
(l)
6:2 FTOH
Ion signal (cps)
Time

163

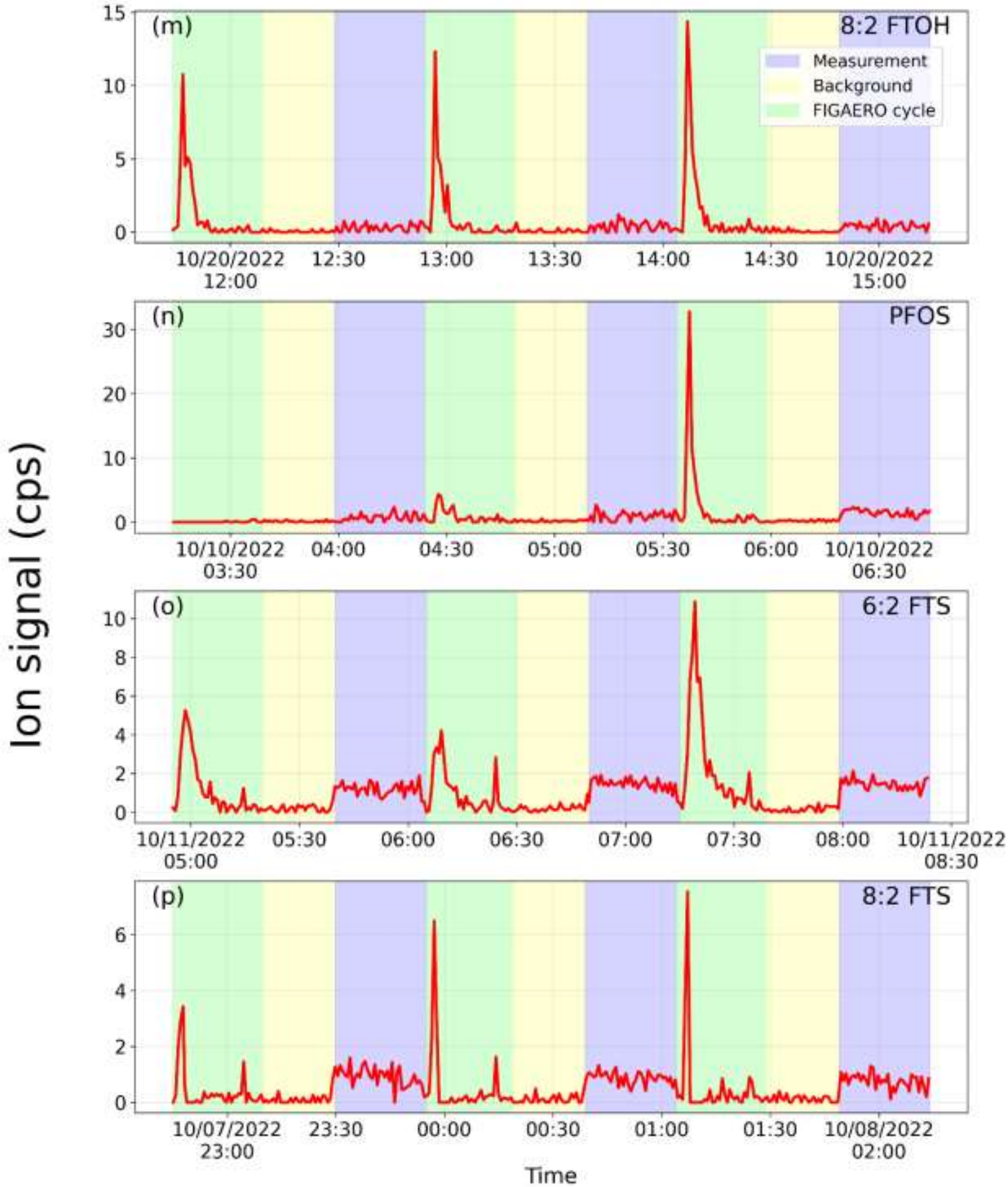

Figure S3. The influence of background measurements (yellow shade) during measurements. Background was taken by displacing the intake of the gas-phase inlet with an overblow of N2 from the headspace of a liquid nitrogen dewar. When ambient measurements resumed (blue shade), signals sharply increased. Each measurement cycle used the corresponding previous background cycle to calculate concentrations and dynamic LODs. The green-shaded regions indicate when the FIGAERO desorption cycle was running.

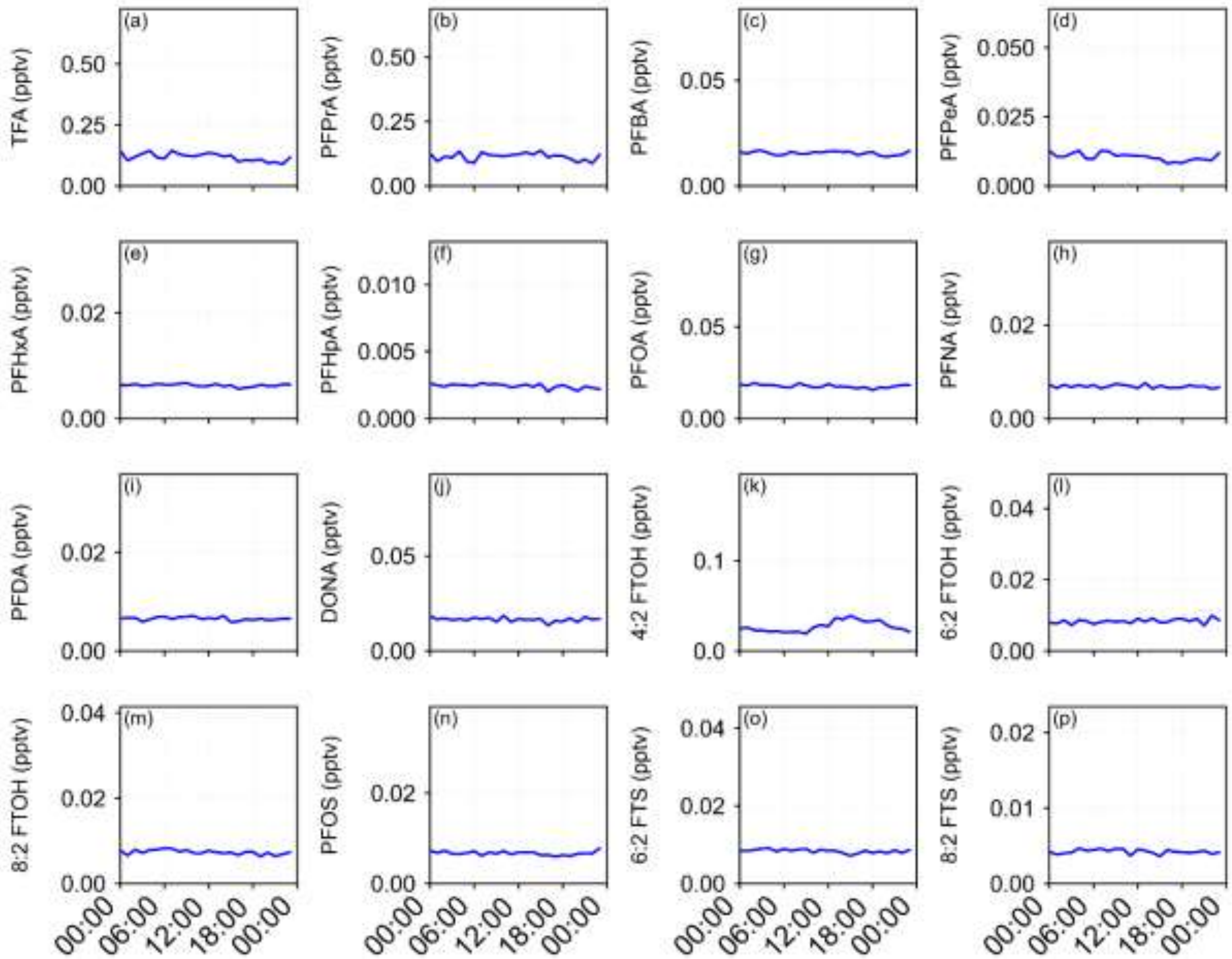

Figure S4. Average diurnal cycles of the gas-phase background for PFAS detected in Houston with I-CIMS, indicating that background signals are not subject to changes in ambient conditions.

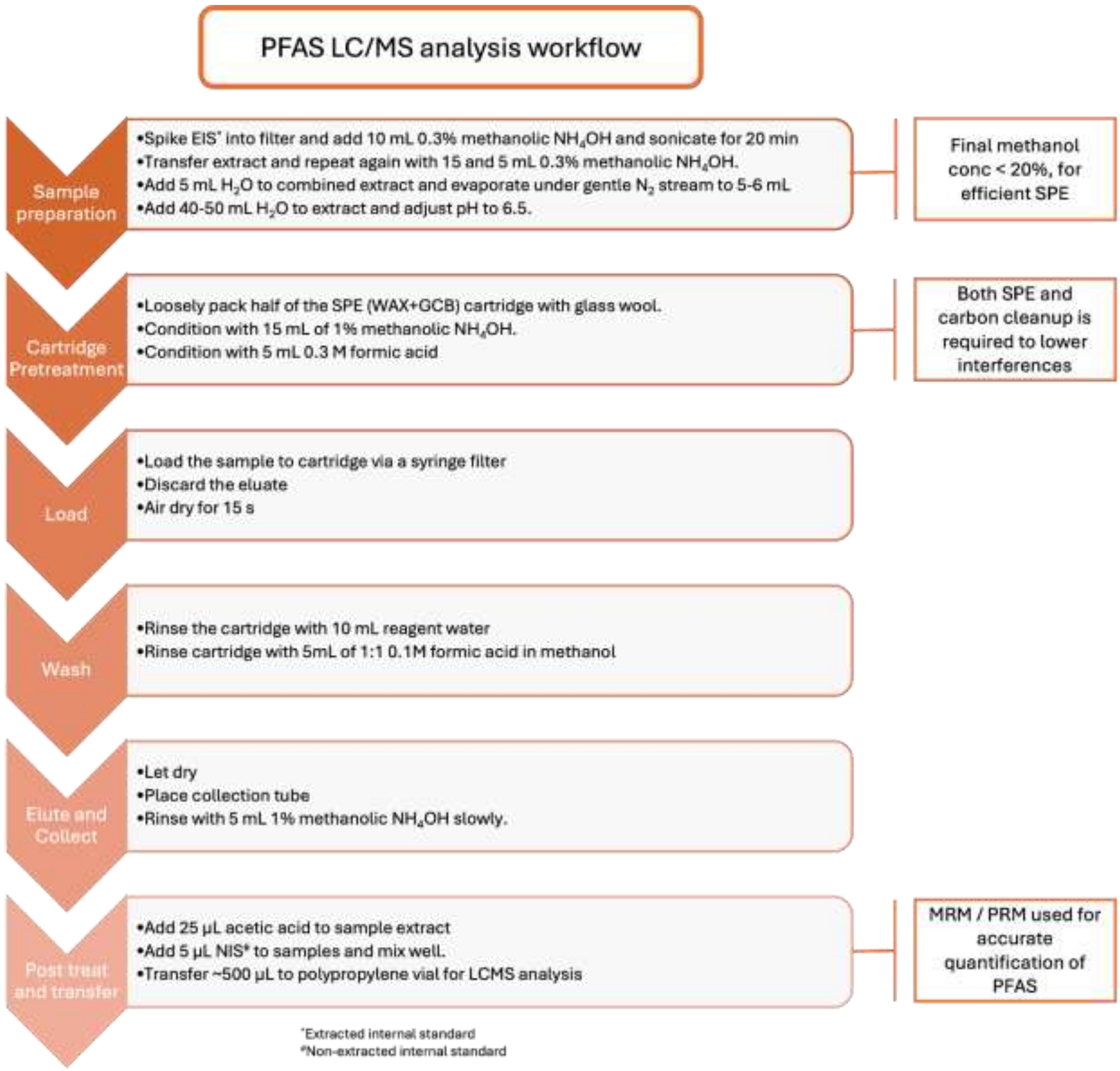

Figure S5. LC-ESI Orbitrap MS analysis workflow for sample preparation, extraction and analysis.

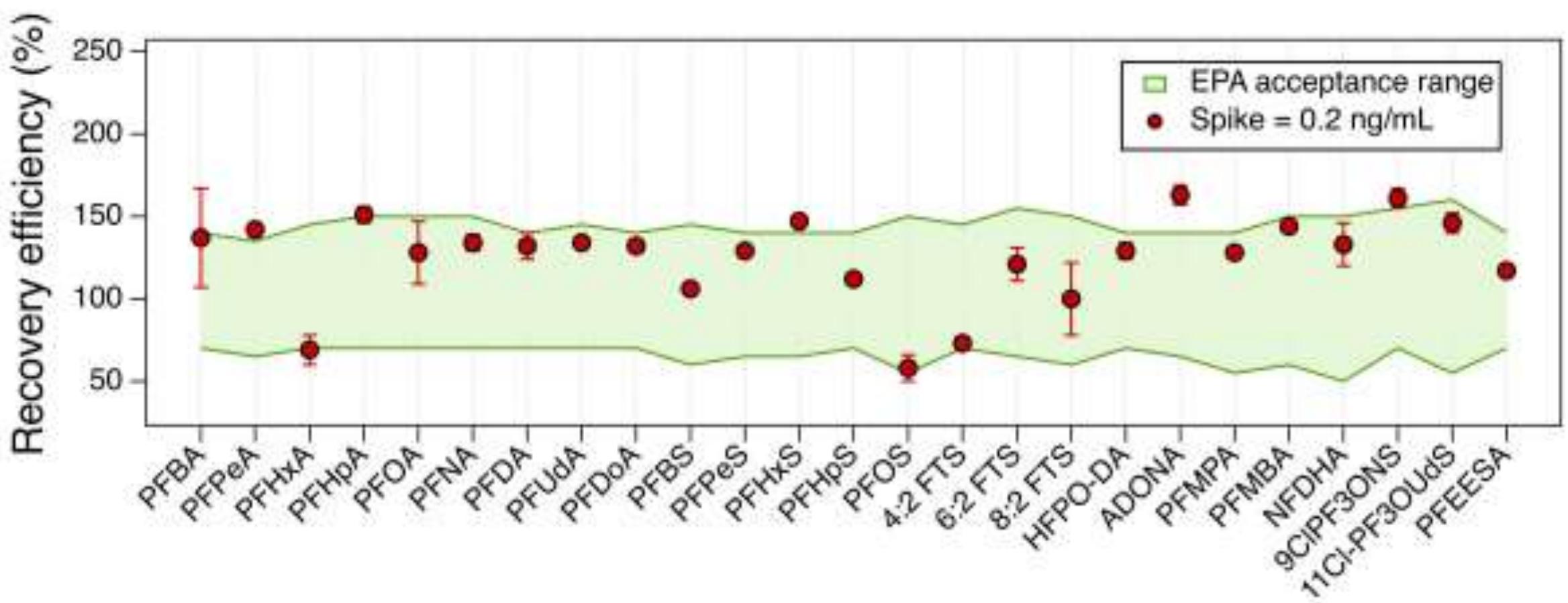

Figure S6. Recovery efficiencies of LC-ESI Orbitrap MS extraction for the 25 target PFAS compounds analyzed in this study, determined by spiking pre-baked quartz fiber filters with native standards at a final extract concentration of 0.2 ng/mL followed by the EPA Method 1633 extraction protocol. Red circles show mean recovery values and error bars indicate one standard deviation across replicate measurements. The green shaded region indicates the analyte-specific EPA Method 1633 acceptance range. PFPeA, PFHxS, ADONA, and 9Cl-PF3ONS exhibited recoveries slightly above the upper acceptance limit; all other analytes fell within the acceptance range. No analytes were excluded from quantitative reporting on the basis of recovery values.

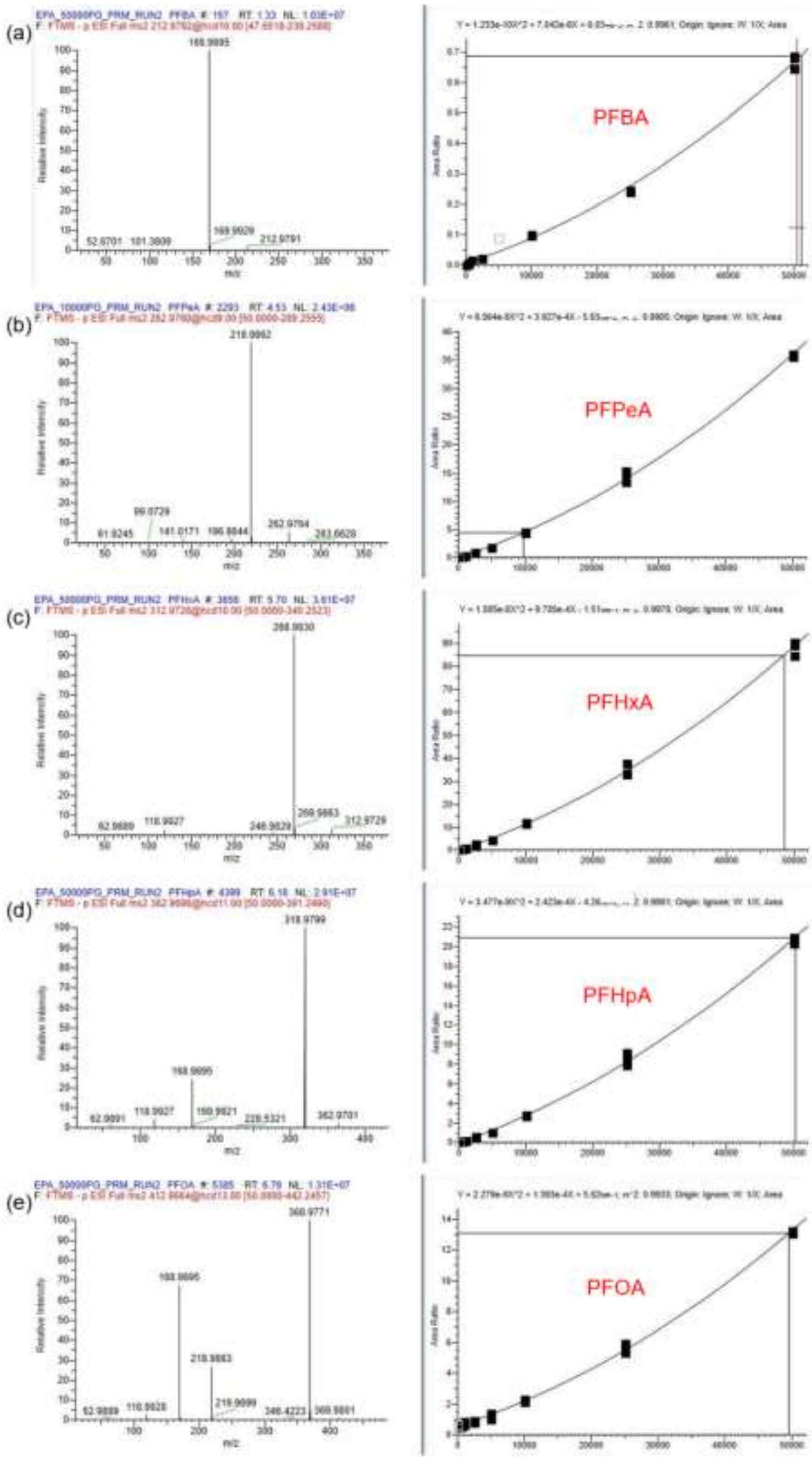
(a)
PFBA
(b)
PFPeA
(c)
PFHxA
(d)
PFHpA
(e)
PFOA

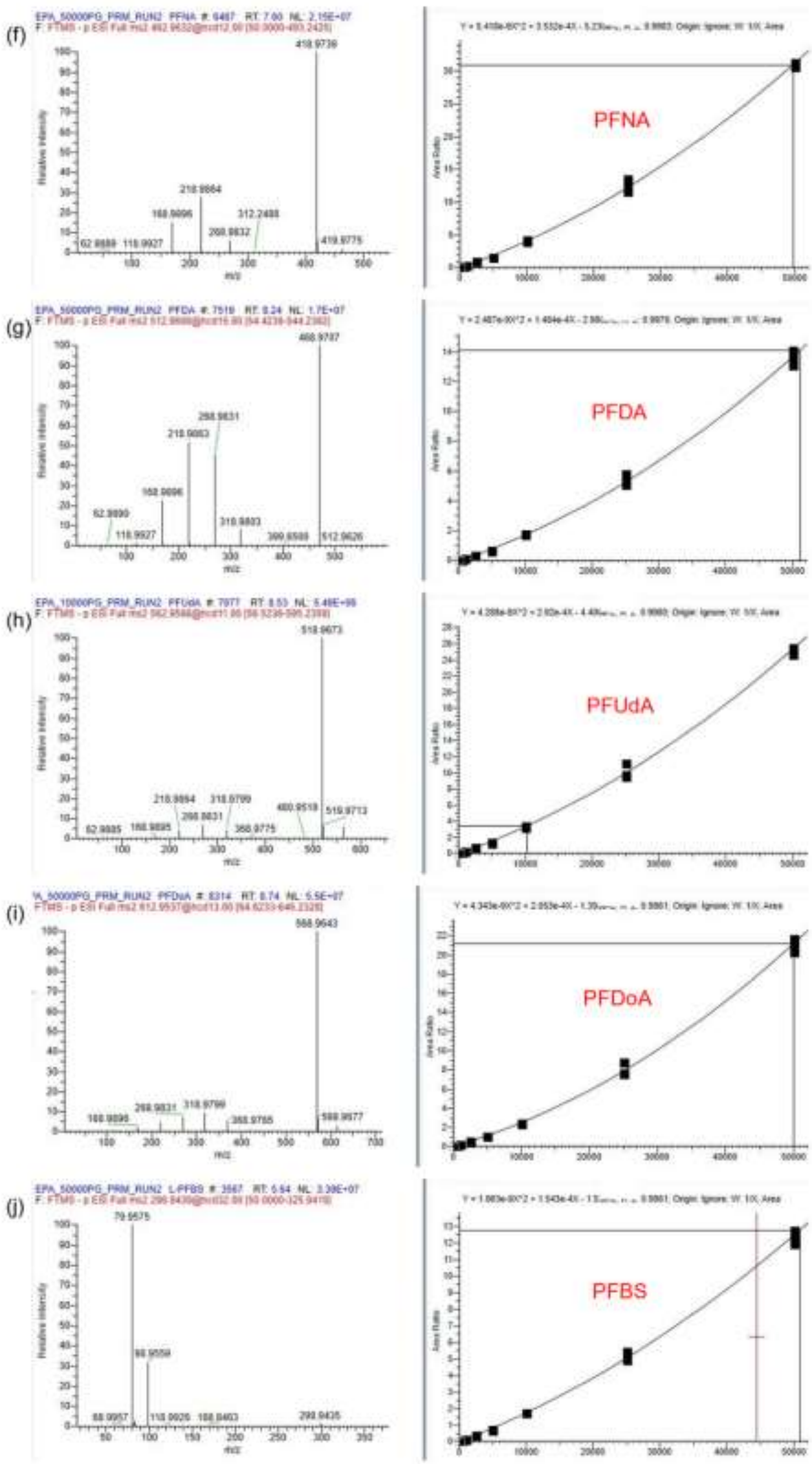
(f)
PFNA
(g)
PFDA
(h)
PFUdA
(i)
PFDoA
(j)
PFBS

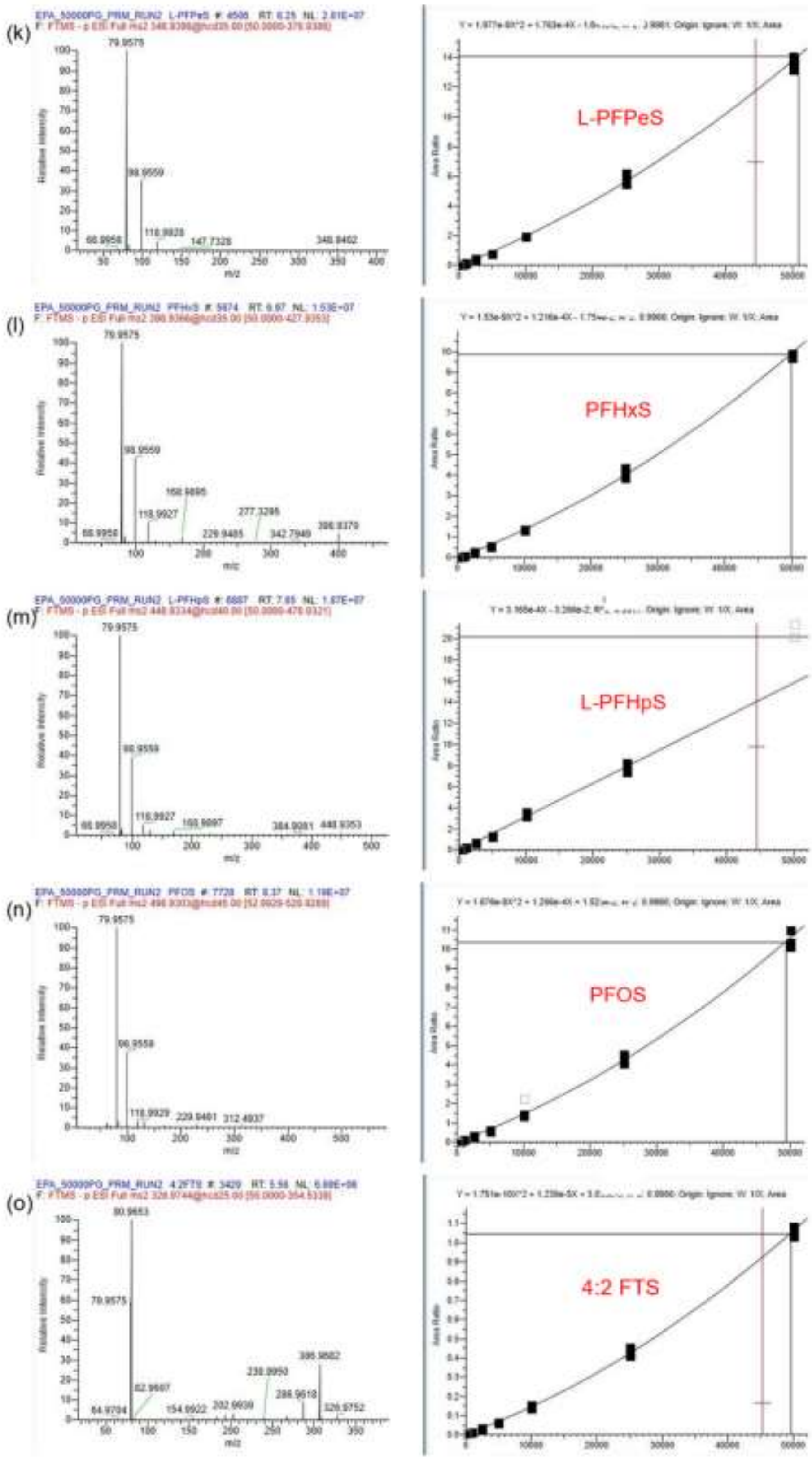
(k)
L-PFPeS
(l)
PFHxS
(m)
L-PFHpS
(n)
PFOS
(o)
4:2 FTS

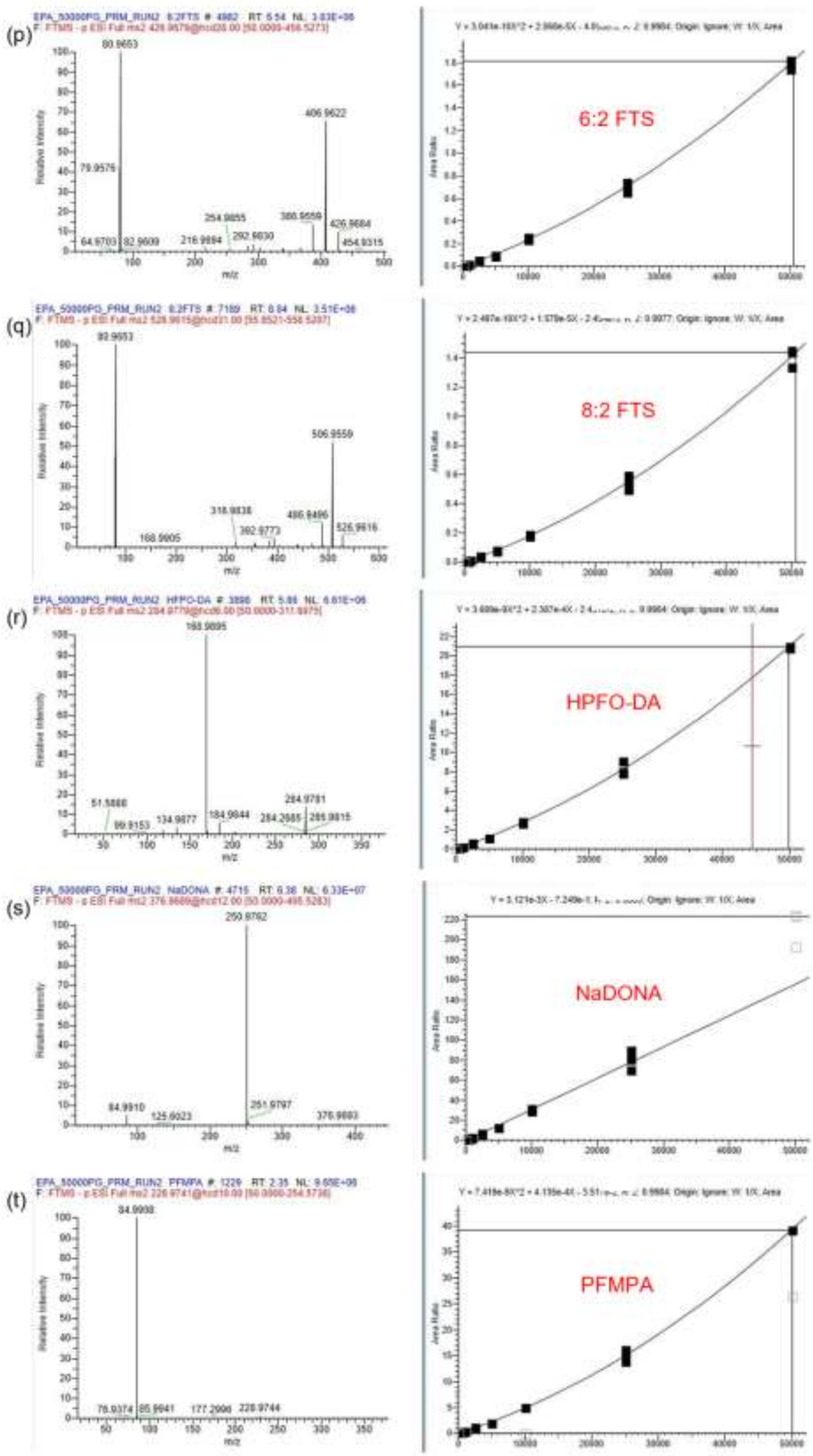
(p)
80.9653
406.9622
79.9576
Relative Intensity
m/z
Area Ratio
6:2 FTS
(q)
80.9653
506.9559
8:2 FTS
(r)
168.9895
HPFO-DA
(s)
250.9762
NaDONA
(t)
84.9908
PFMPA

(u)

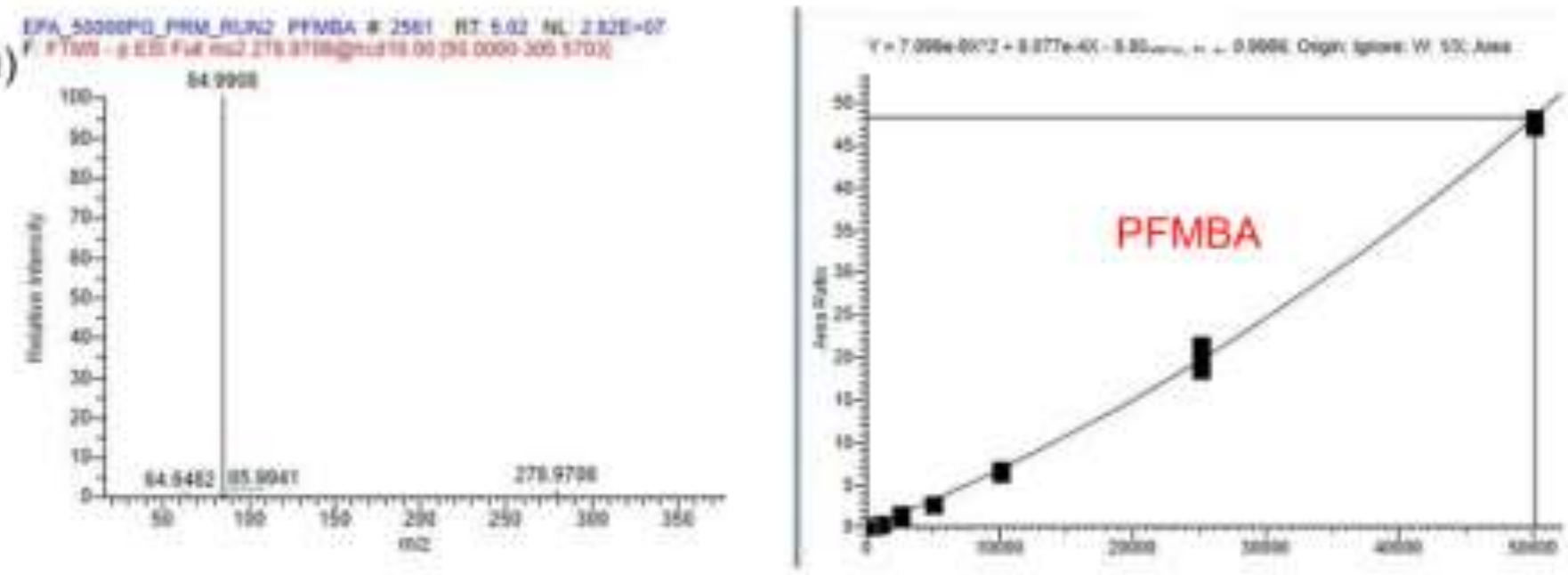

84.9908
PFMBA

(v)

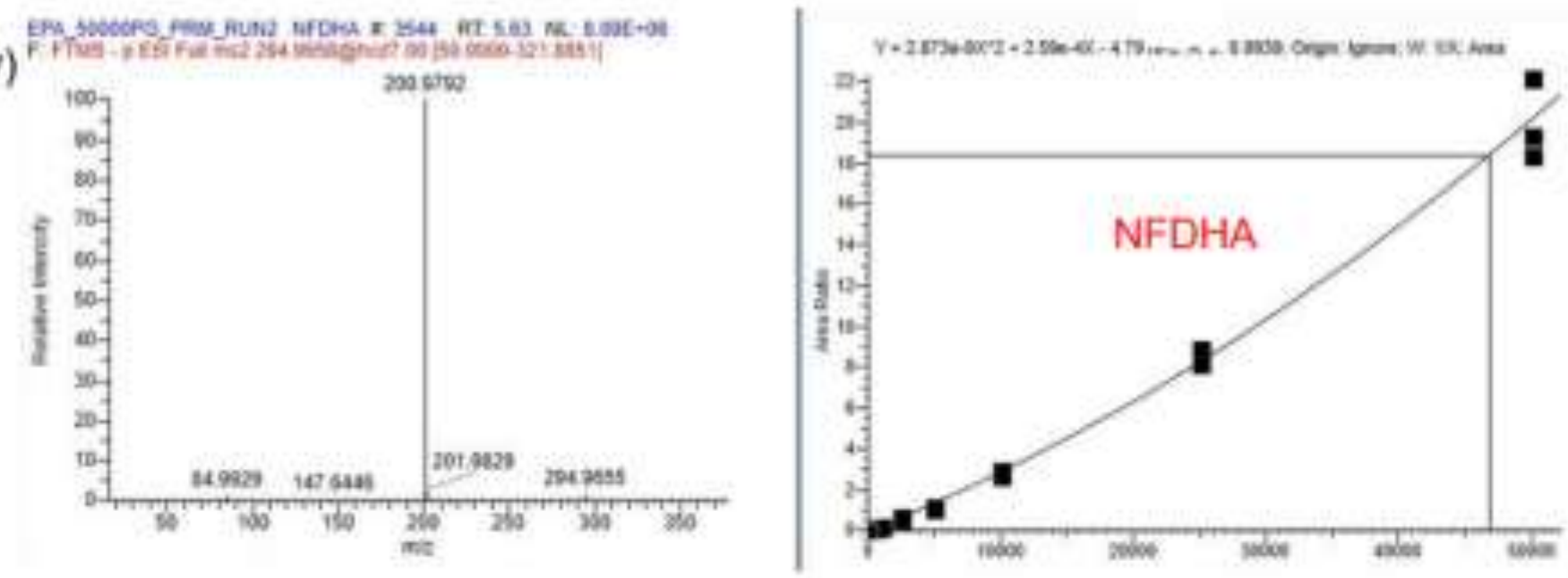

200.9792
NFDHA

(w)

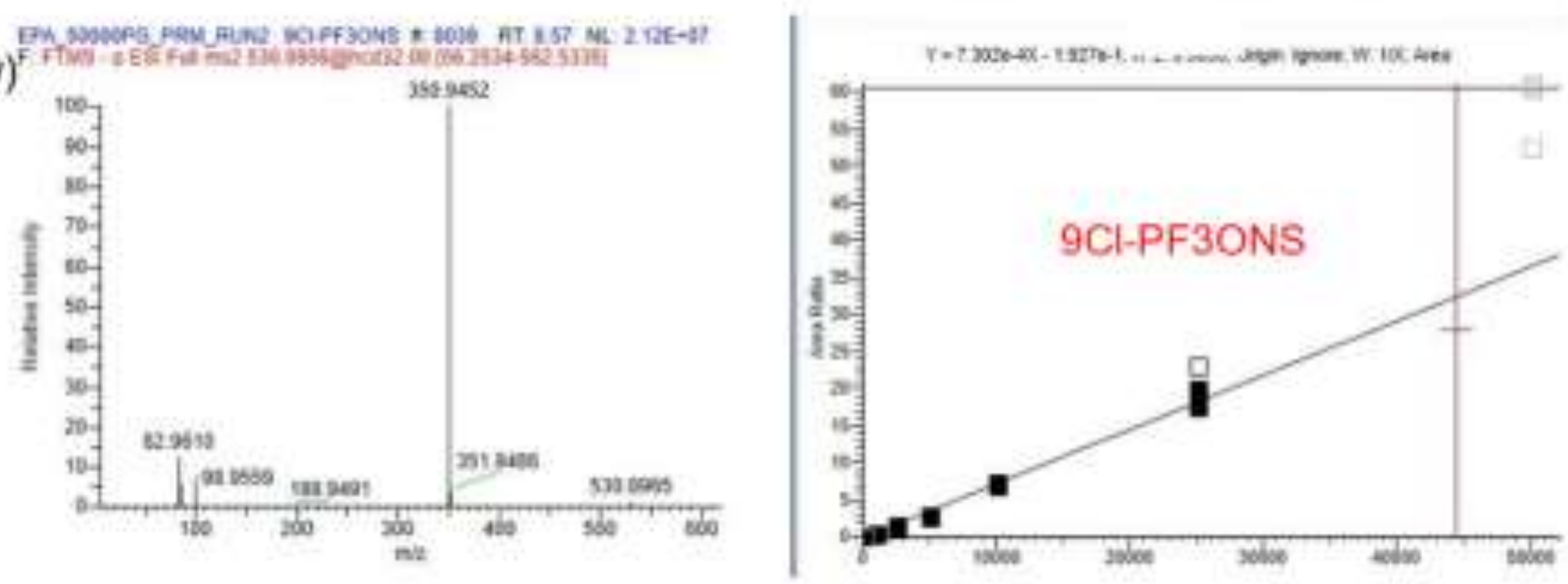

350.9452
9Cl-PF3ONS

(x)

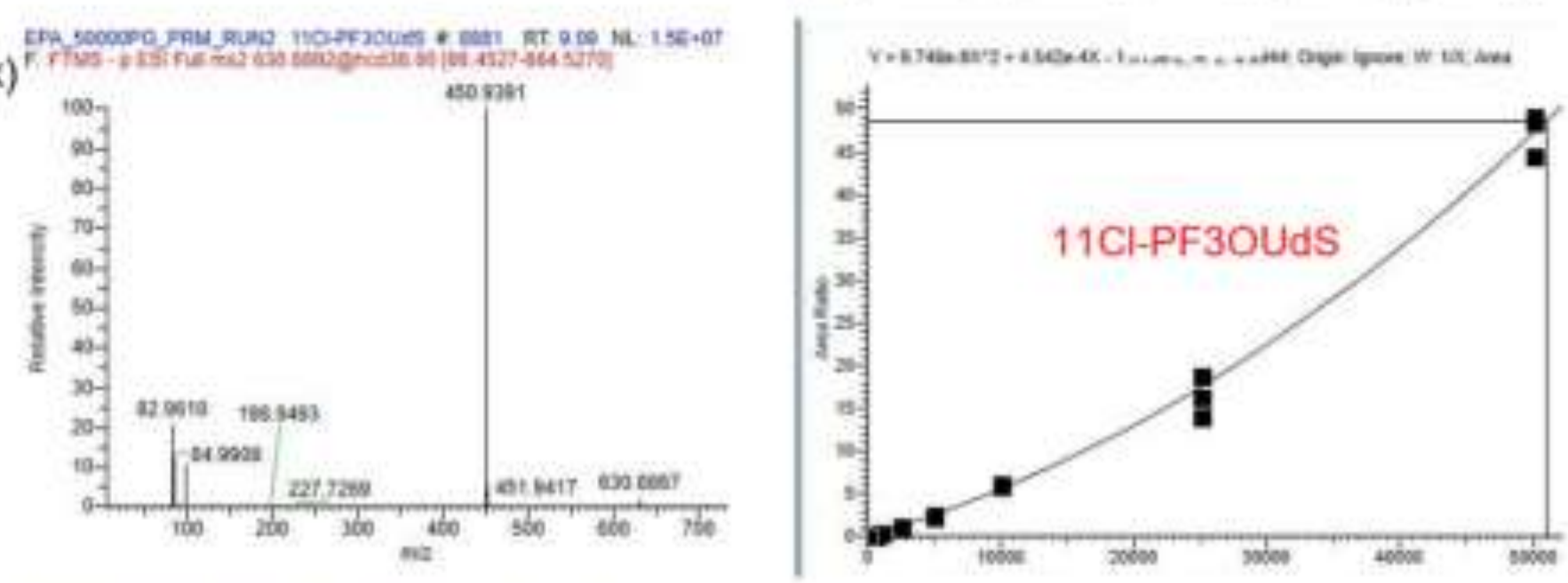

450.9391
11Cl-PF3OUdS

(y)

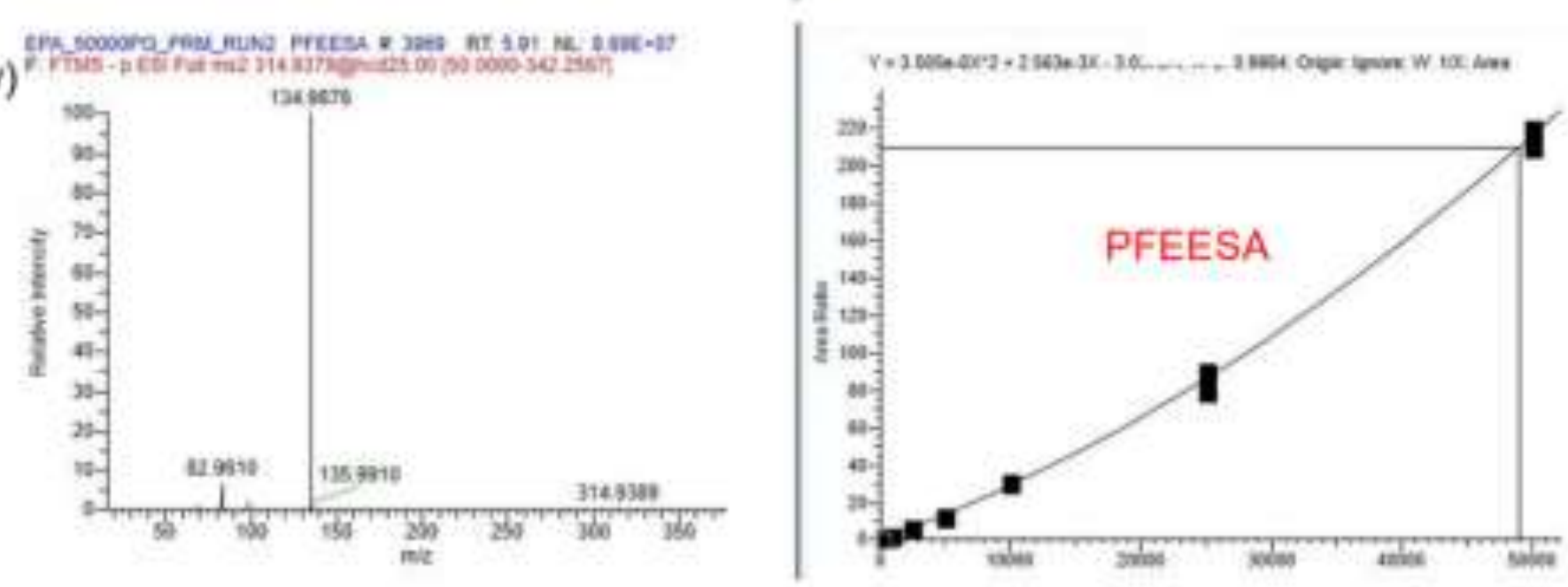

134.9876
PFEESA

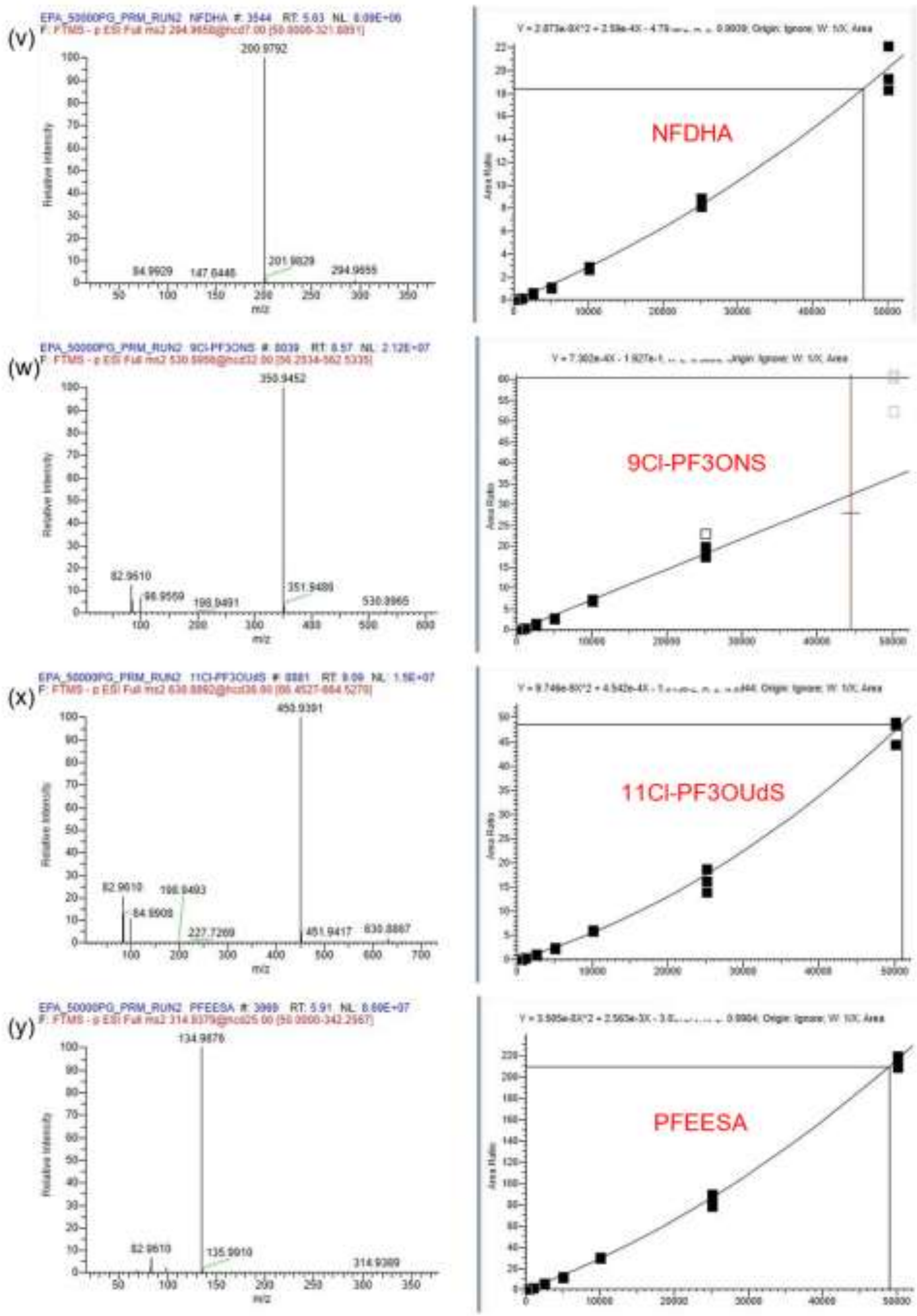

Figure S7. (a-y) MS/MS fragmentation ions used for quantification and resulting calibration curves of PFAS from calibration of the LC-ESI Orbitrap MS according to EPA Method 1633.

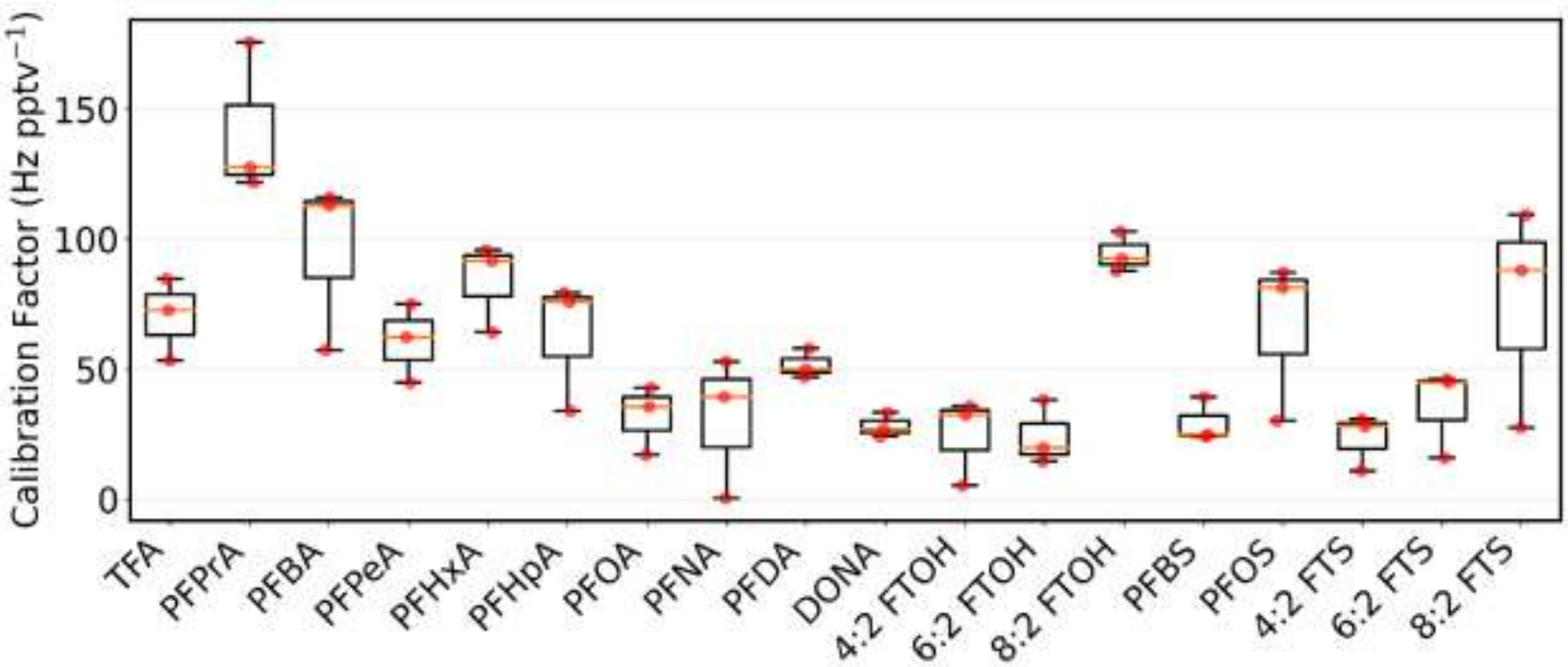

Figure S8. Box plots of I-CIMS calibration factors for PFAS. Three calibration trials were run for each compound. Solid red lines denote the median value for calibration factors for each compound. The boxes span the 25th to 75th percentile values for calibration factors based on three trials, and the whiskers denote the full range of calibration factors.

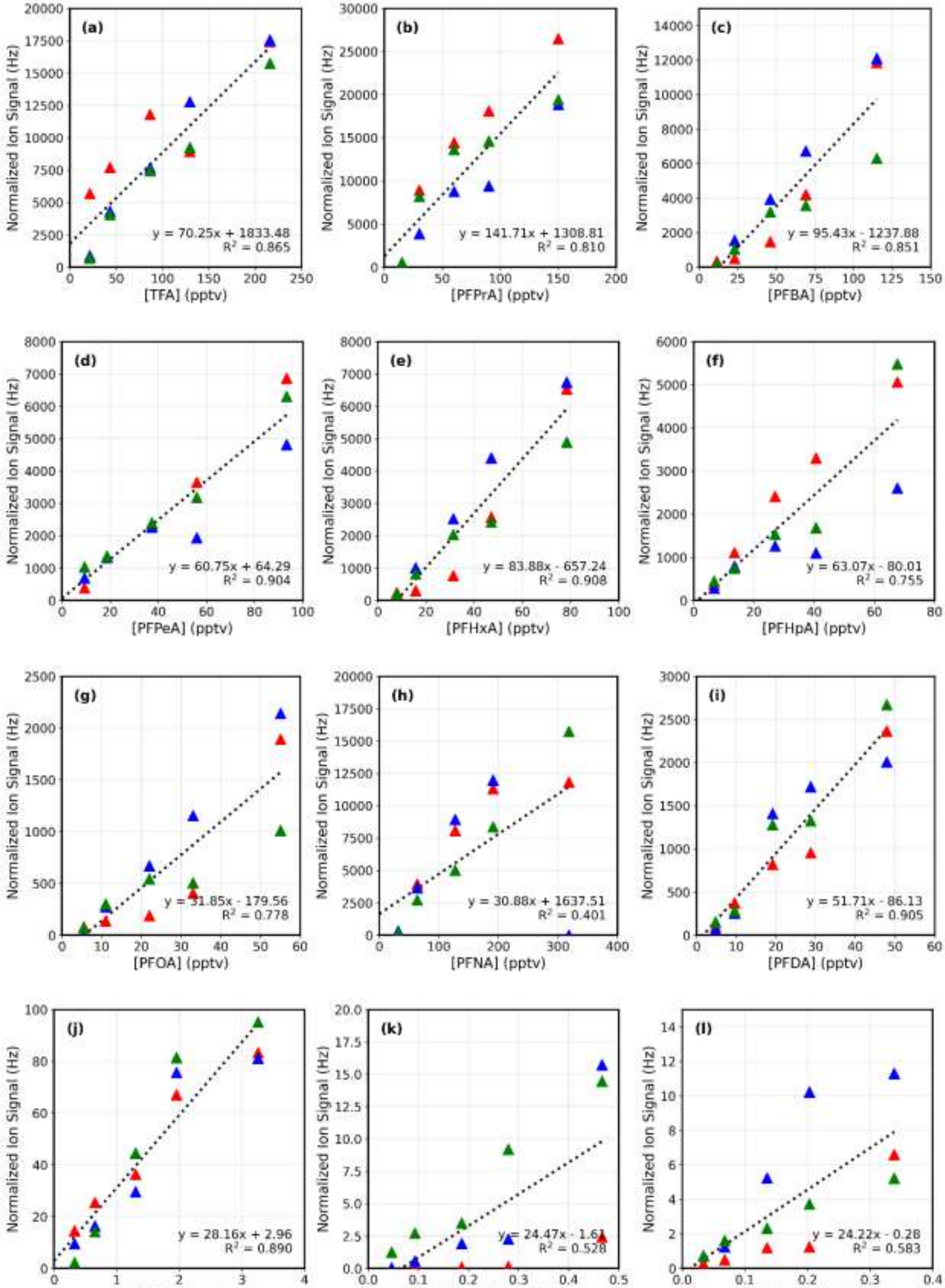
(a)
y = 70.25x + 1833.48
R² = 0.865
Normalized Ion Signal (Hz)
[TFA] (pptv)
(b)
y = 141.71x + 1308.81
R² = 0.810
[PFPrA] (pptv)
(c)
y = 95.43x - 1237.88
R² = 0.851
[PFBA] (pptv)
(d)
y = 60.75x + 64.29
R² = 0.904
[PFPeA] (pptv)
(e)
y = 83.88x - 657.24
R² = 0.908
[PFHxA] (pptv)
(f)
y = 63.07x - 80.01
R² = 0.755
[PFHpA] (pptv)
(g)
y = 31.85x - 179.56
R² = 0.778
[PFOA] (pptv)
(h)
y = 30.88x + 1637.51
R² = 0.401
[PFNA] (pptv)
(i)
y = 51.71x - 86.13
R² = 0.905
[PFDA] (pptv)
(j)
y = 28.16x + 2.96
R² = 0.890
[DONA] (pptv)
(k)
y = 24.47x - 1.61
R² = 0.528
[4:2 FTOH] (pptv)
(l)
y = 24.22x - 0.28
R² = 0.583
[6:2 FTOH] (pptv)

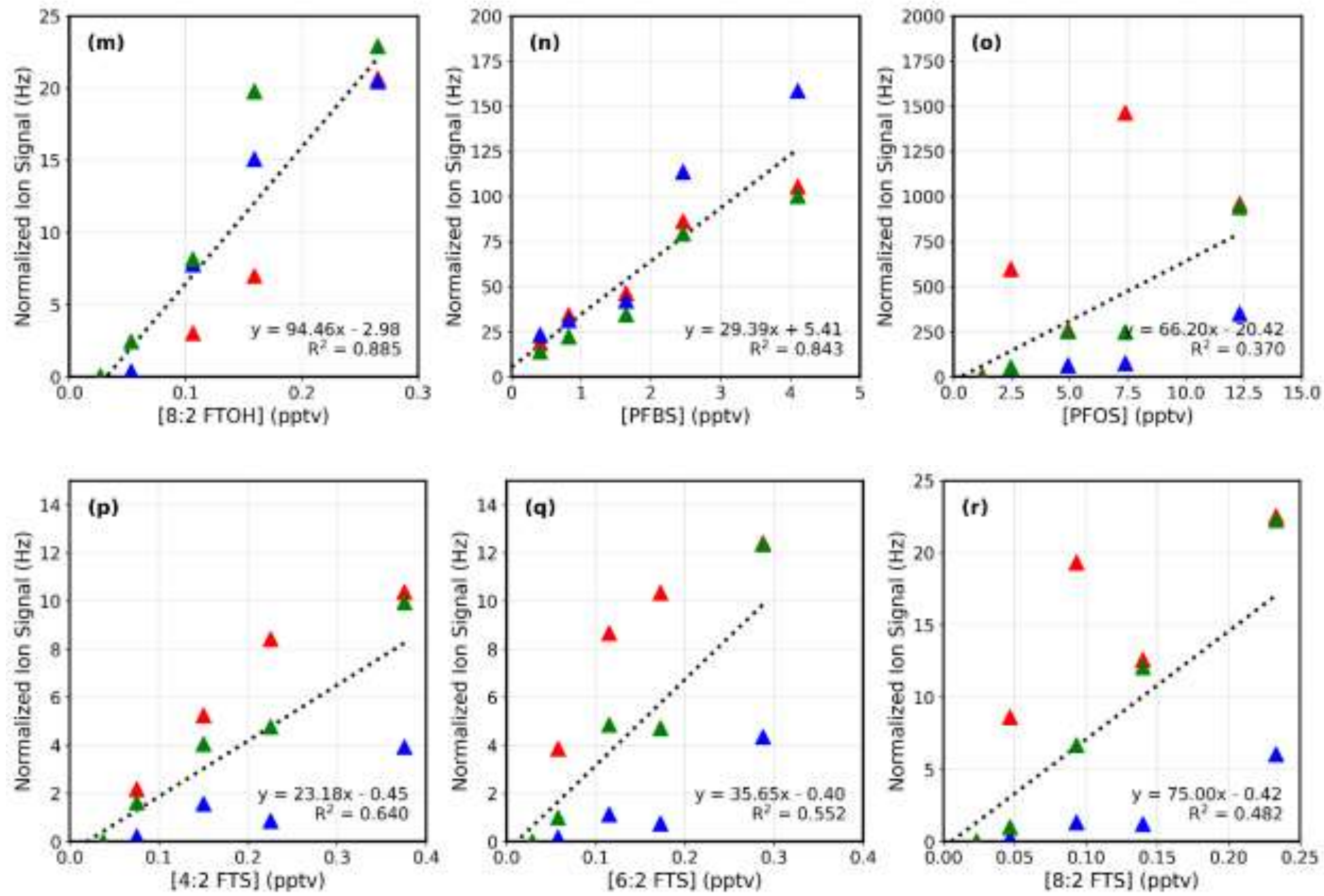

Figure S9. (a-r) Calibration curves for PFAS compounds calibrated with the I-CIMS for this study. Red, green, and blue triangles indicate individual calibration trials. Three trials were run for each compound. Calibration curves were based on the linear fit of all trials (black dotted line) with the linear regression equation displayed on each graph.

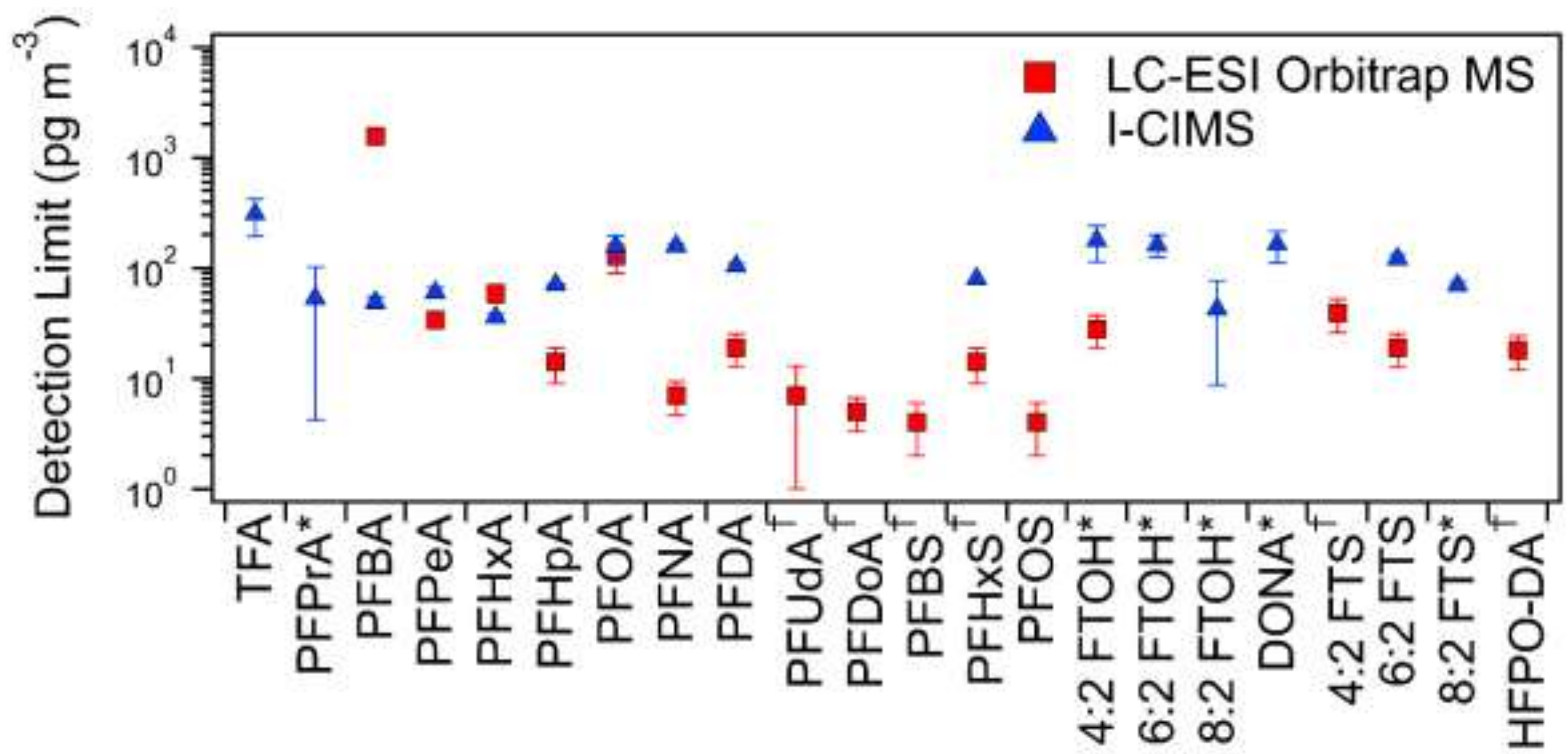

Figure S10. Limits of detection (LODs) for particle-phase PFAS compounds detected by LC-ESI Orbitrap MS (red squares) and gas-phase PFAS detected by the I-CIMS (blue triangles). Error bars for I-CIMS represent the standard deviation in LOD calculation based on variability in background signals. Error bars for LC-ESI Orbitrap MS LODs are based on results from 7 different calibration trials.

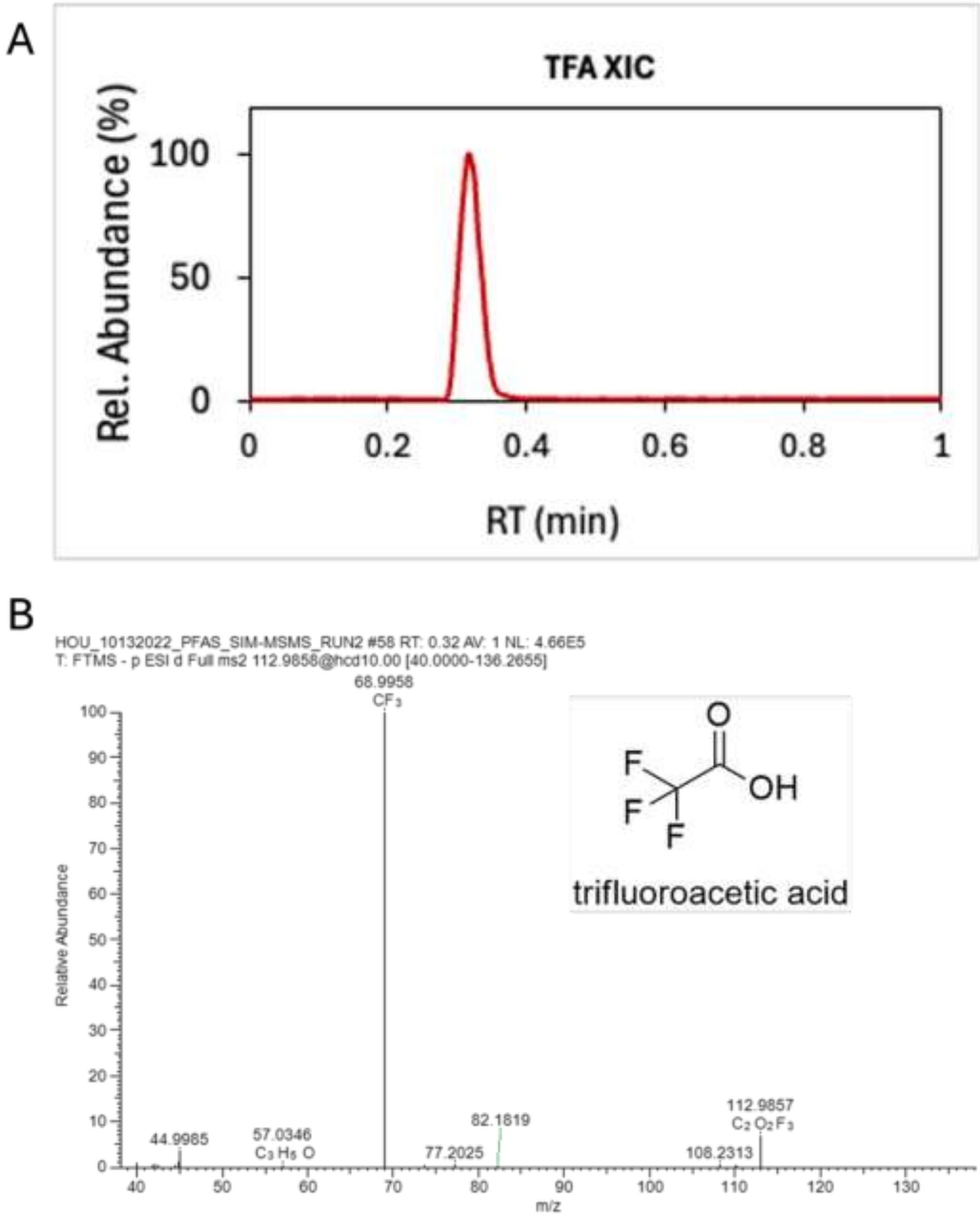

Figure S11. Trifluoroacetic acid (TFA) detected in Houston particulate matter sample (A) UPLC-ESI-MS/MS extracted ion chromatogram (m/z = 112.9856) of detected TFA. (B) MS/MS fragmentation of TFA showing a $-CF_3$ fragment hence providing structural confirmation.

227 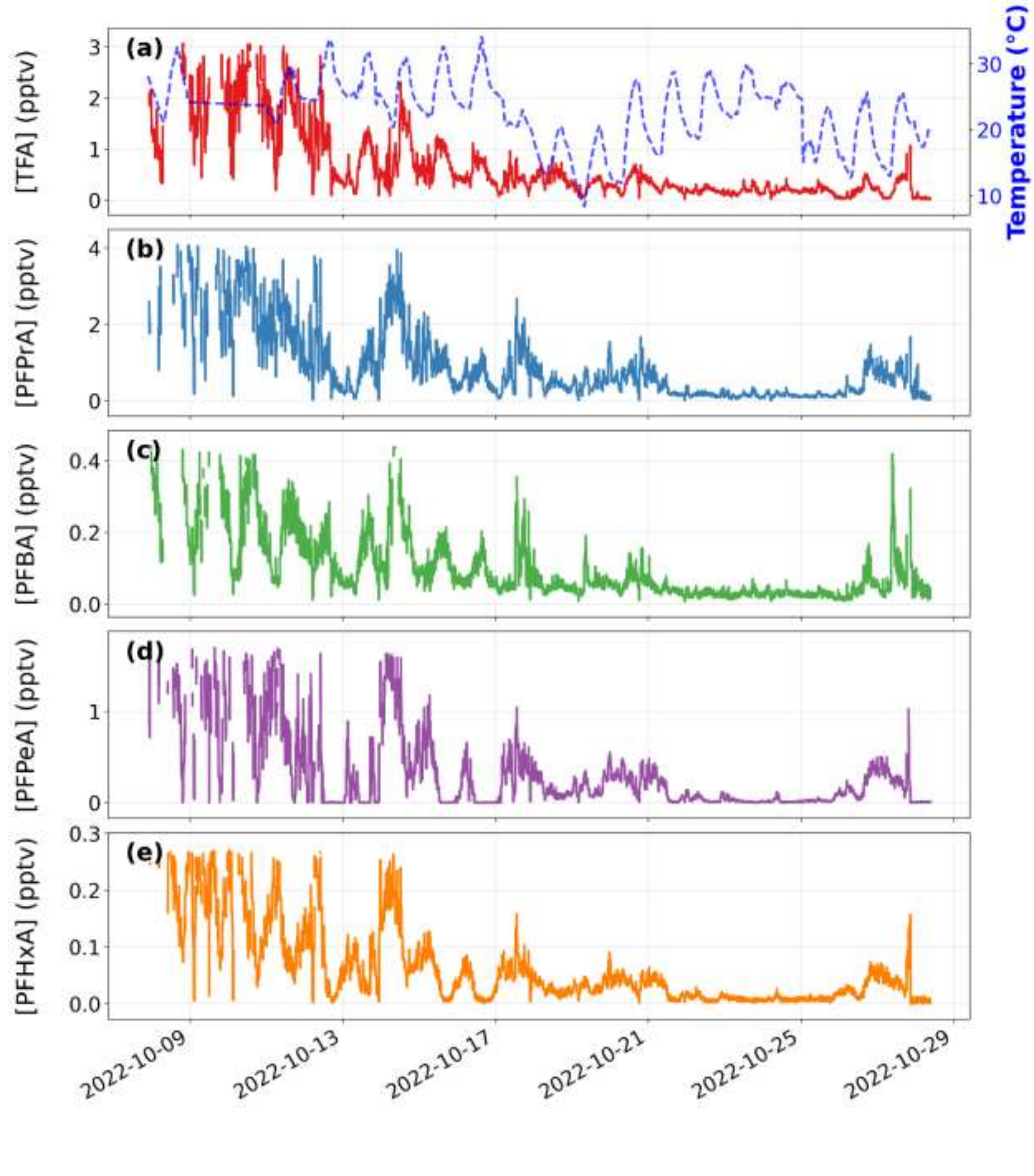

(a)
[TFA] (pptv)
Temperature (°C)
(b)
[PFPrA] (pptv)
(c)
[PFBA] (pptv)
(d)
[PFPeA] (pptv)
(e)
[PFHxA] (pptv)
2022-10-09
2022-10-13
2022-10-17
2022-10-21
2022-10-25
2022-10-29

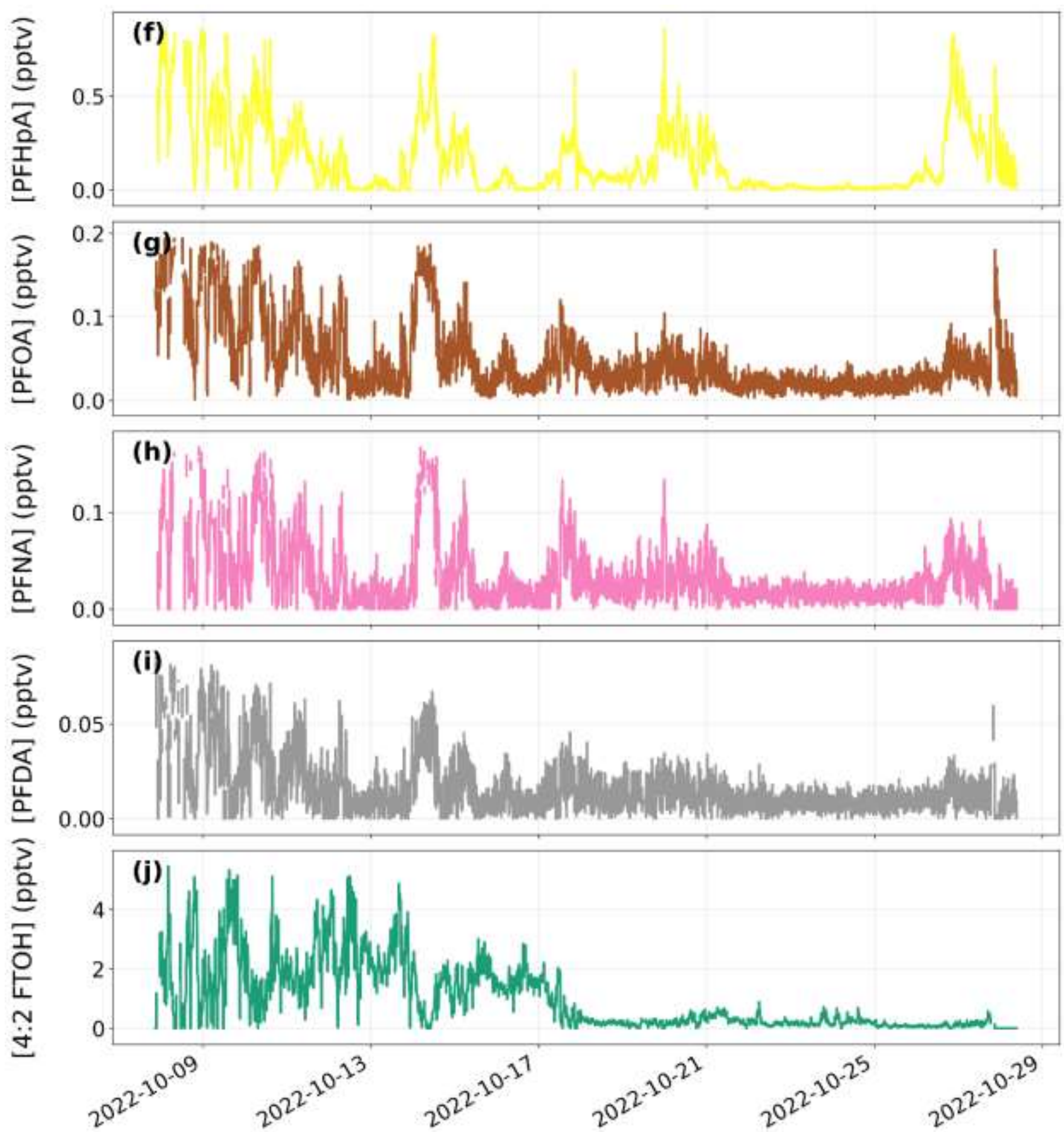
(f)
[PFHpA] (pptv)
0.5
0.0
(g)
[PFOA] (pptv)
0.2
0.1
0.0
(h)
[PFNA] (pptv)
0.1
0.0
(i)
[PFDA] (pptv)
0.05
0.00
(j)
[4:2 FTOH] (pptv)
4
2
0
2022-10-09
2022-10-13
2022-10-17
2022-10-21
2022-10-25
2022-10-29

228

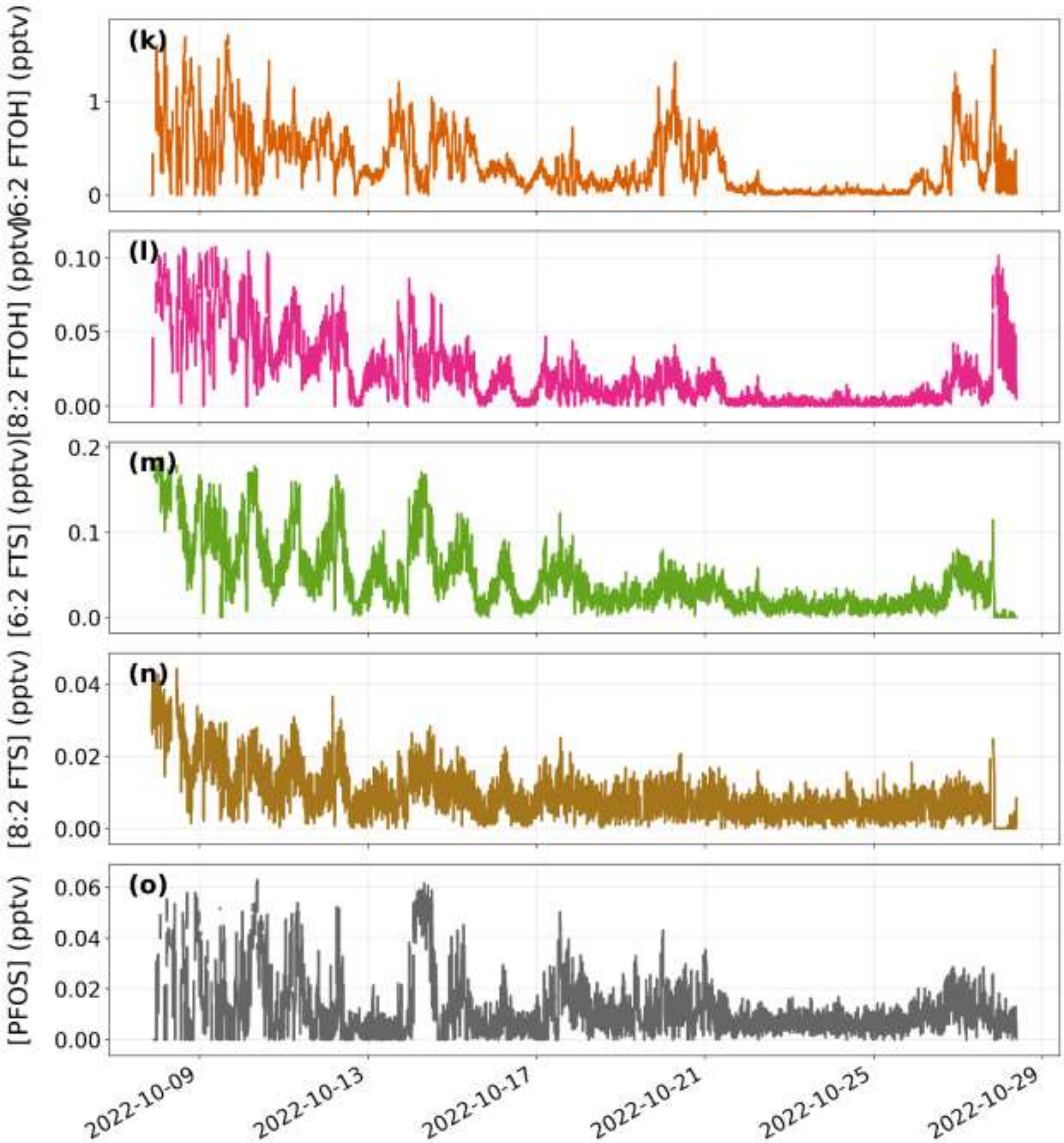

Figure S12. (a-o) Full time series of all gas-phase PFAS detected by I-CIMS throughout the measurement campaign.

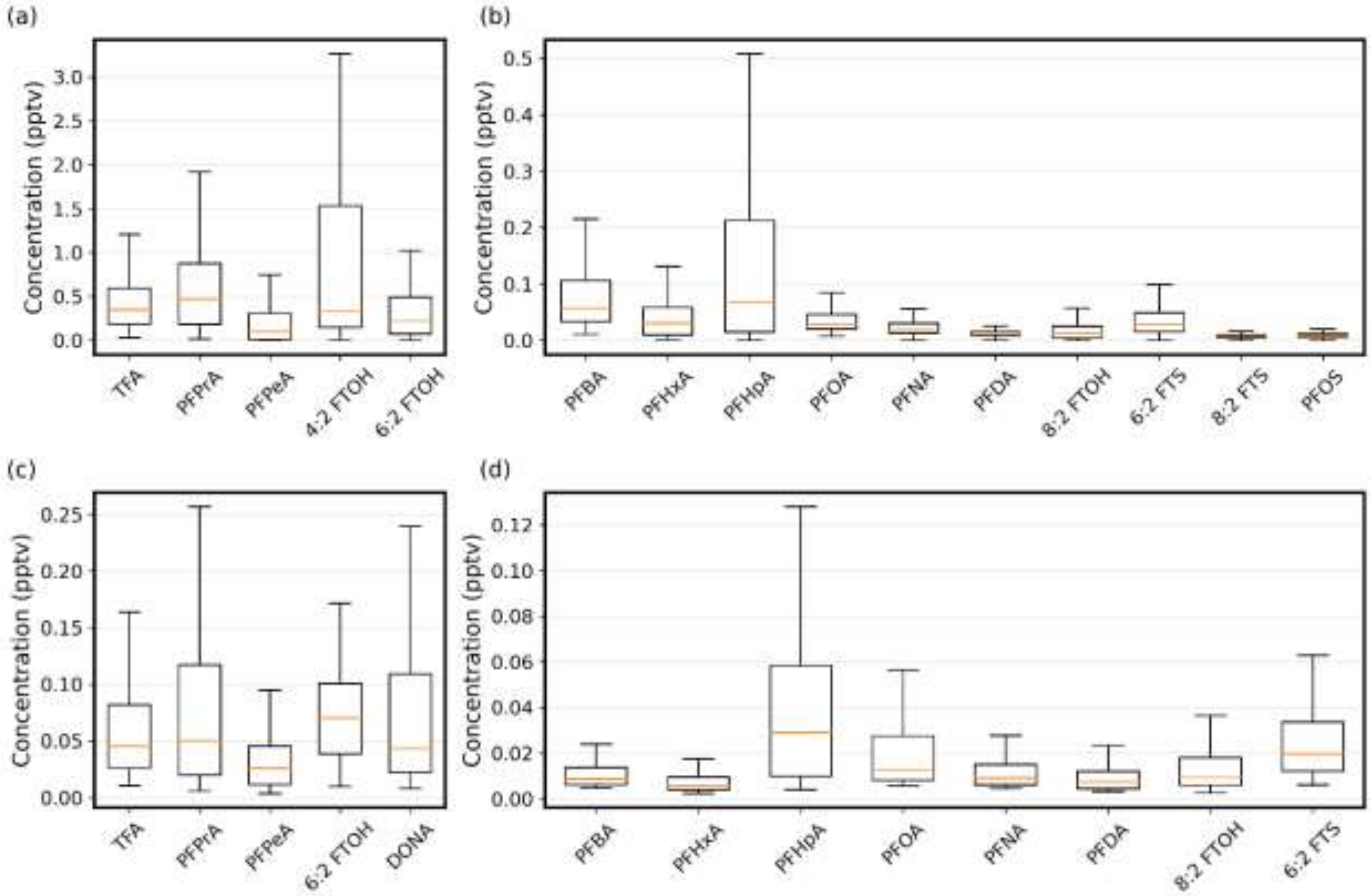

Figure S13. Moving 24-hour averages for (a, b) gas-phase PFAS, (c, d) particle-phase PFAS observed during the measurement period. Figures were split due to different concentration ranges. The boxes span the 25th to 75th percentile values and the whiskers denote the full range.

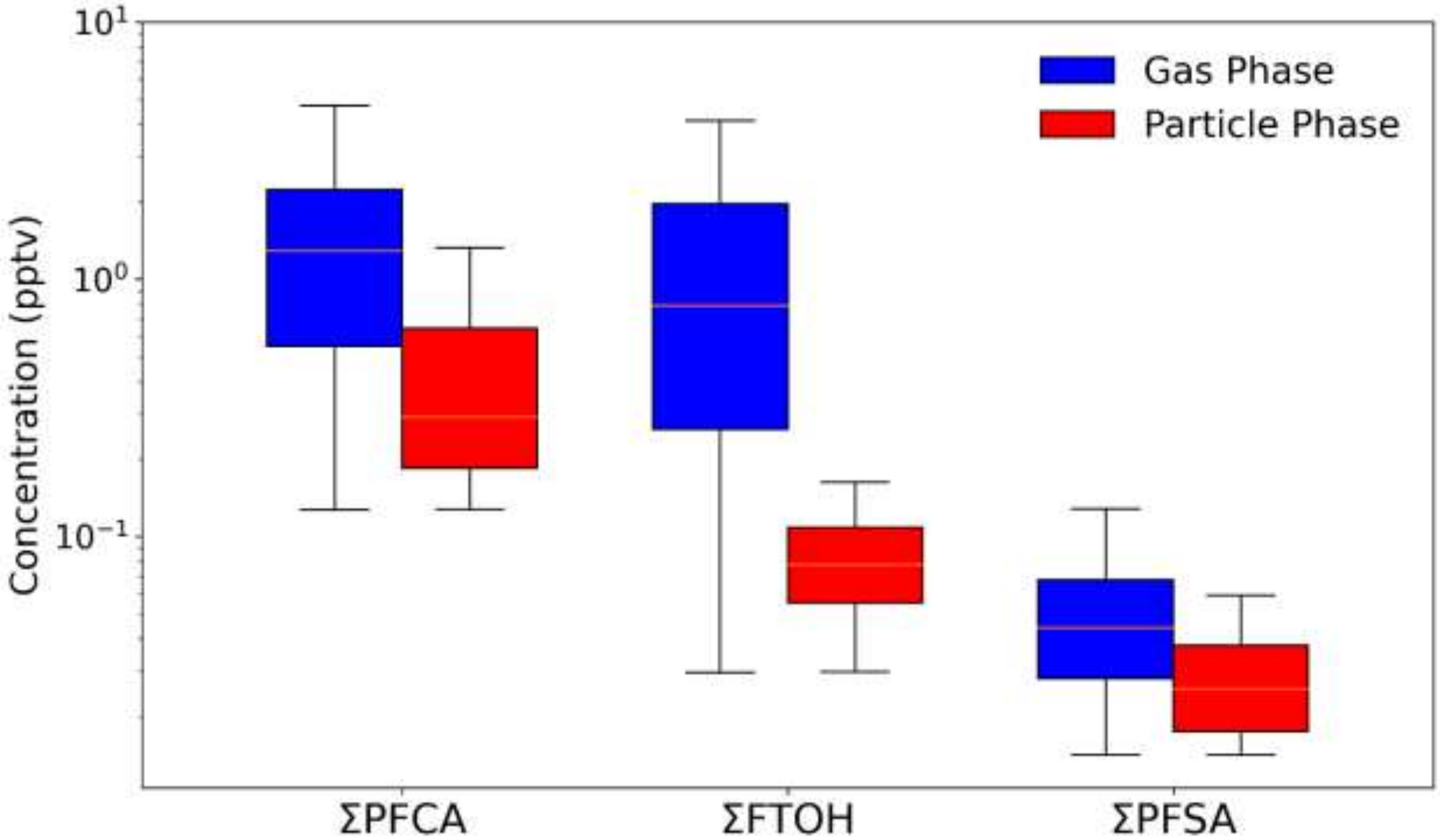

Figure S14. 24-hour mean concentrations of ΣPFAS classes measured in Houston in the I-CIMS gas-phase (blue), and particle phase (red).

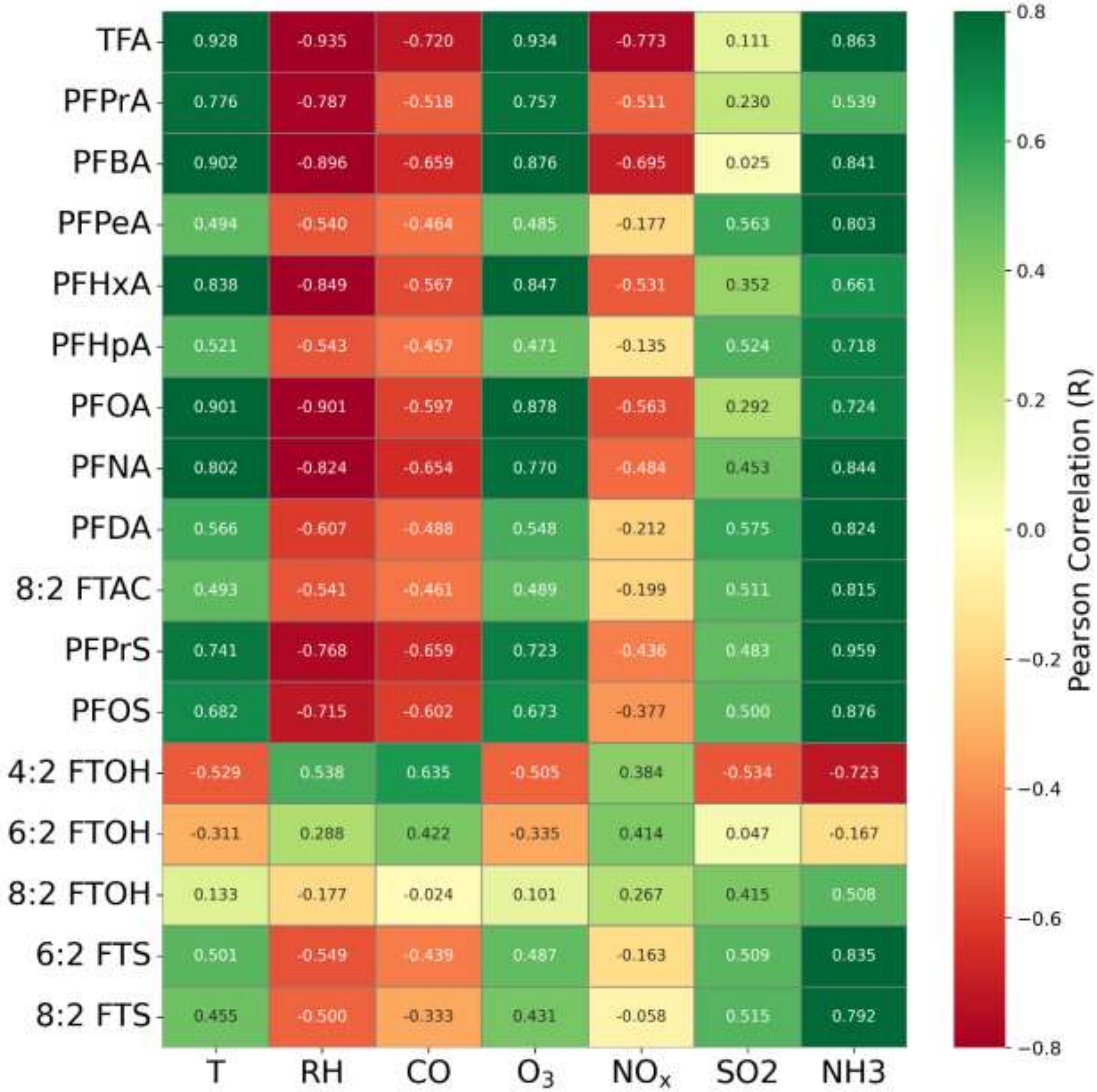

Figure S15. Correlations between gas-phase PFAS and environmental variables/pollutants. Gas-phase PFAS correlated well with temperature and were negatively correlated with RH, with the exception of FTOH compounds. PFCA and PFSA compounds Were positively correlated with O_3 and negatively correlated with NO_x, which is consistent with photochemical formation mechanisms.

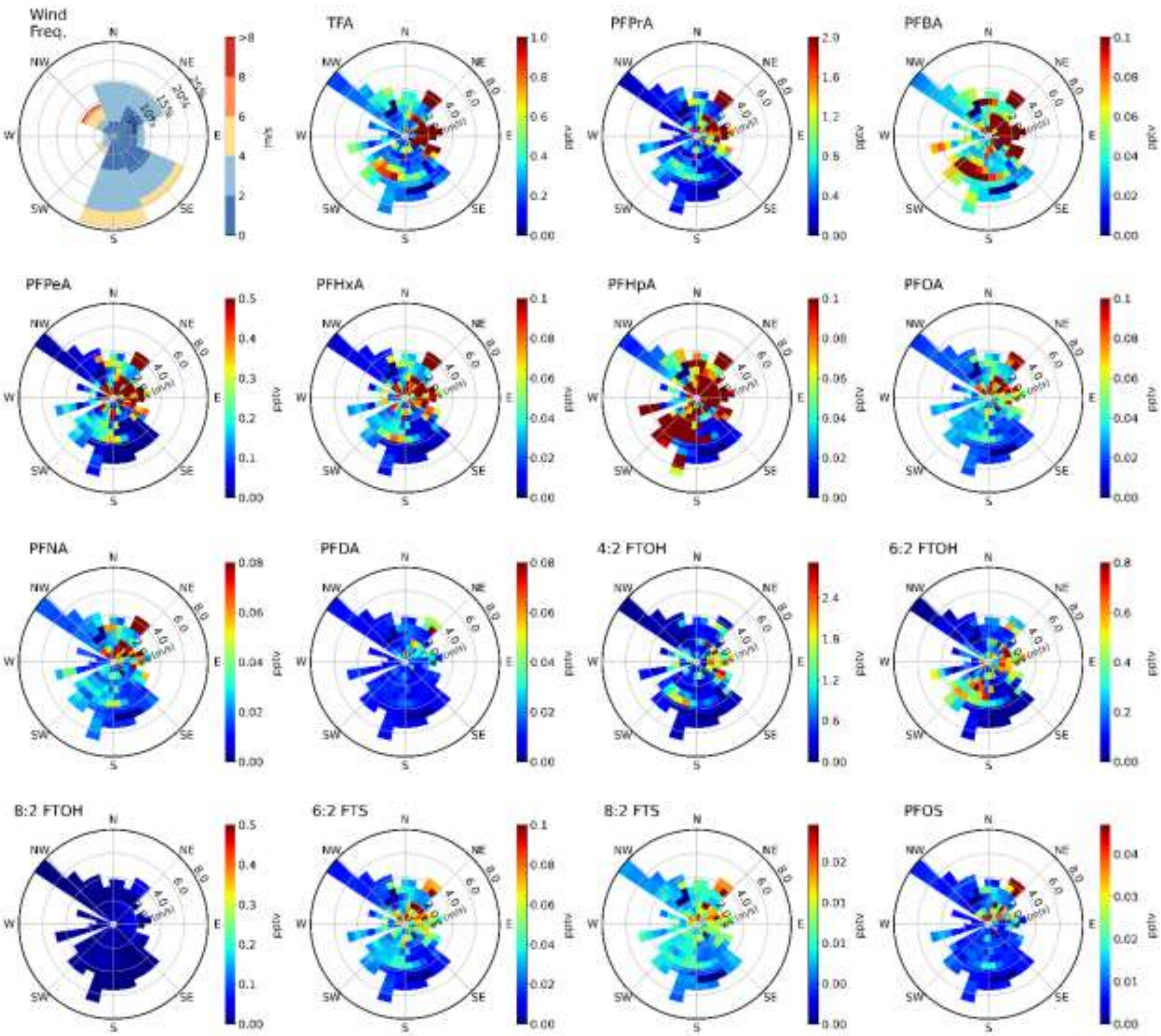

Figure S16. Gas-phase PFCA correlation with wind speed and direction. Easterly and northeasterly winds tended to have the highest concentrations of gas-phase PFCAs, but low concentrations of PFCAs were detected from all directions.

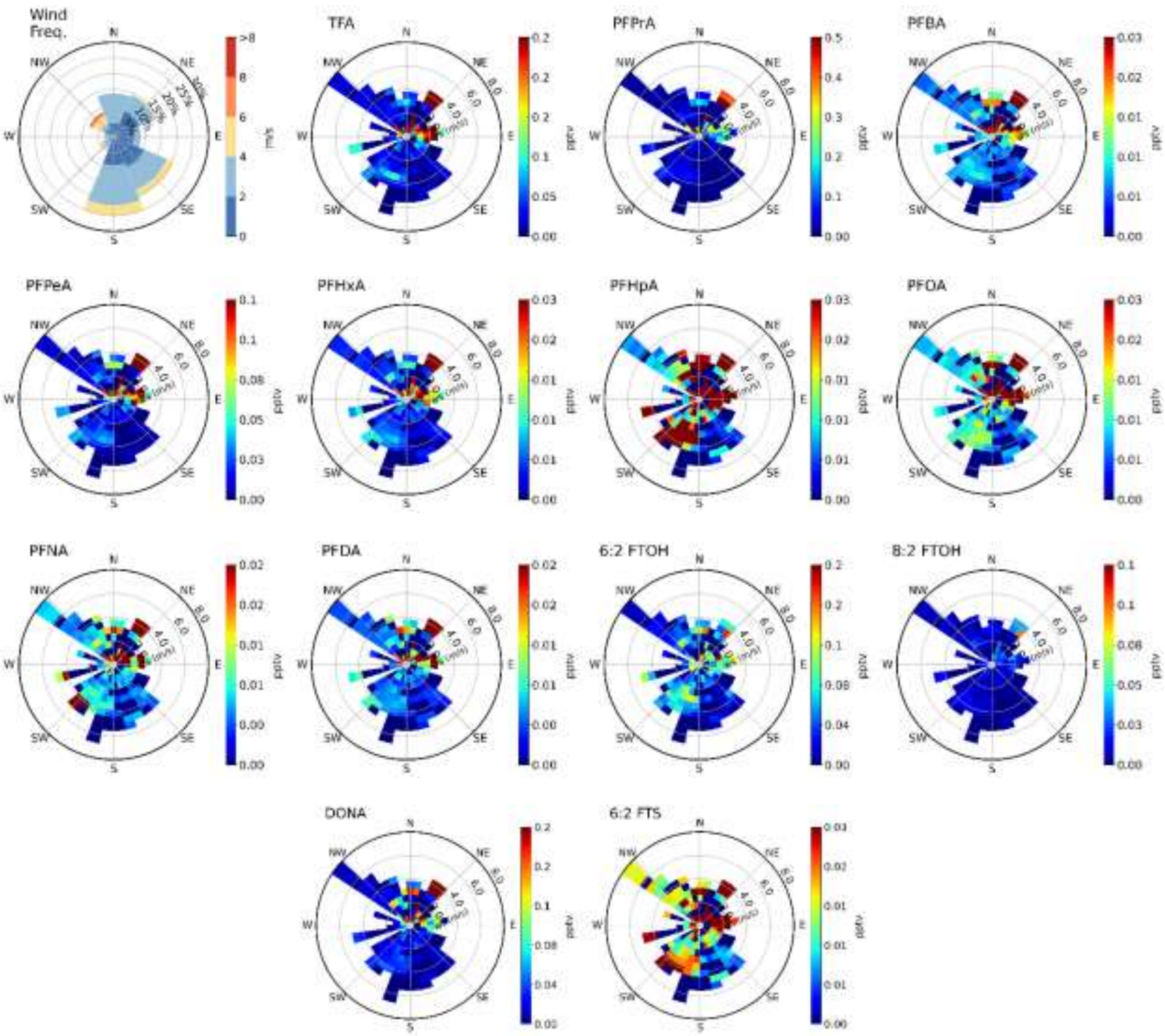

Figure S17. Particle-phase PFAS correlation with wind speed and direction. Like gas-phase, the highest concentrations were detected from the East and Northeast. Particle-phase PFOA had elevated concentrations from a variety of directions, including low-speed northerly winds and moderate southwesterly winds.

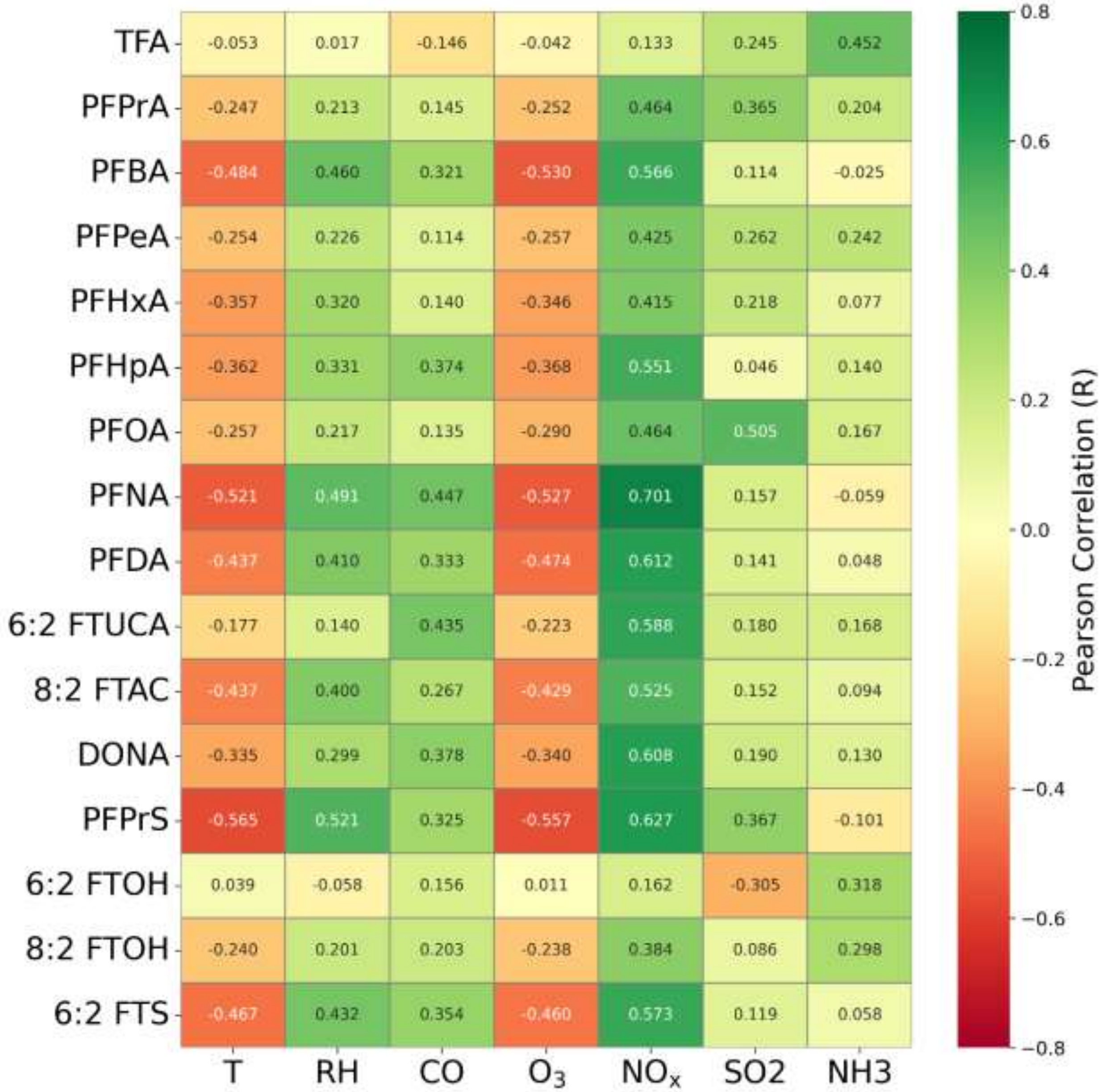

Figure S18. Correlations between particle-phase PFAS and environmental variables/pollutants. Particle-phase PFAS correlated positively with RH and negatively with temperature, which implies that low temperatures favor partitioning into the particle-phase. Positive correlations with RH are consistent with PFAS entering aqueous particles.

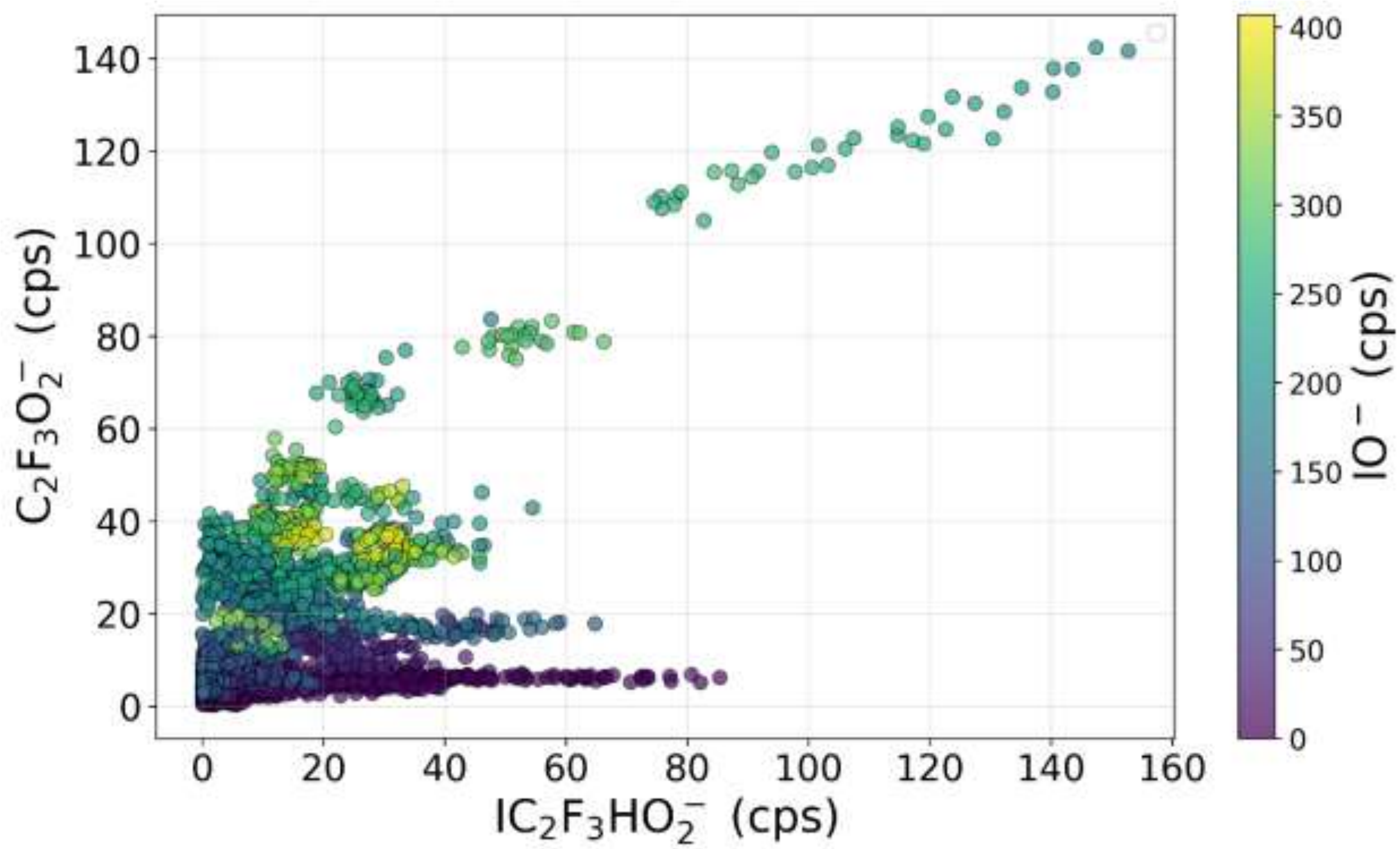

Figure S19. The ion signal for the conjugate base of trifluoroacetic acid ($C_2F_3O_2^-$) as a function of the ion signal of the iodide adduct of trifluoroacetic acid ($IC_2F_3HO_2^-$) Points are colored according to the corresponding ion signal of IO-. Low IO- signals biased the formation of the iodide adduct. At moderate IO- signals, the correlation between the conjugate base and iodide adduct correlated linearly.

Table S1. Voltage settings used for the HR-ToF I-CIMS in this study. The voltages were optimized to oxidized organic molecules using the Thuner automatic mass spectrometer tuning software (Tofwerk, Switzerland).

Component	Setting (V)
Nozzle	0
SSQ Entry Plate	-1.7
SSQ Front	0.3
SSQ Back	6.2
Lens Skimmer	8.2
Skimmer	10.1
BSQ Front	12.1
BSQ Back	9.6
Skim 2	16.7
Ion Lens 2	64.9
Deflector Flange	117.2
Deflector	123.0

Table S2. CAS numbers, linear formulae and structures for all PFAS compounds detected in this study.

Compound	Formula	CAS	Structure
TFA	CF_3COOH	76-05-1	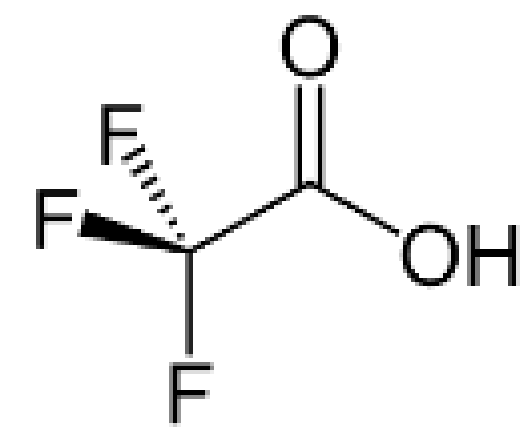
PFPrA	CF_3CF_2COOH	422-64-0	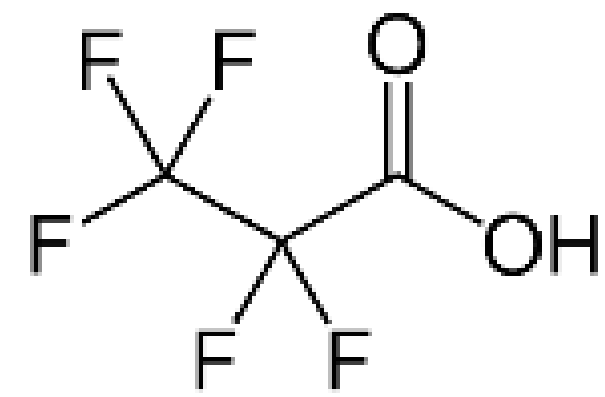
PFBA	$CF_3(CF_2)_2$ $COOH$	375-22-4	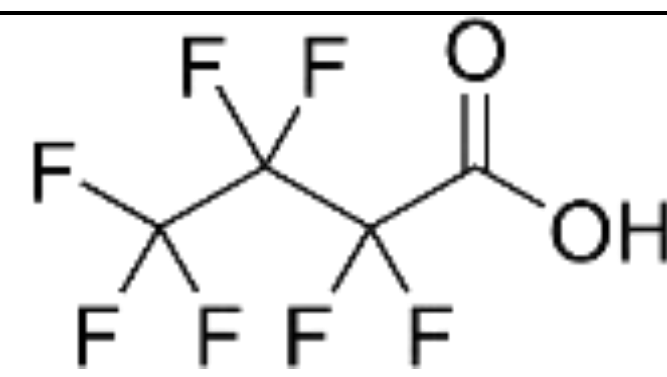
PFPeA	$CF_3(CF_2)_3COOH$	2706-90-3	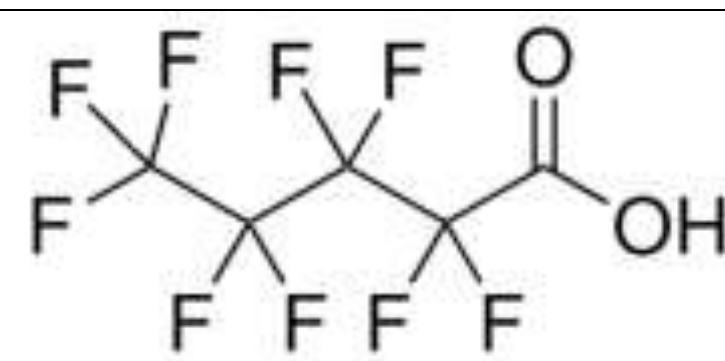
PFHxA	$CF_3(CF_2)_4COOH$	307-24-4	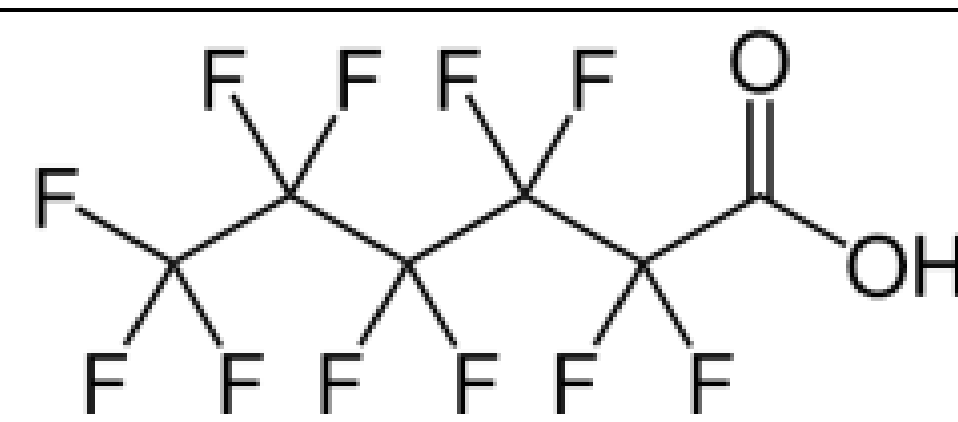
PFHpA	$CF_3(CF_2)_5COOH$	375-85-9	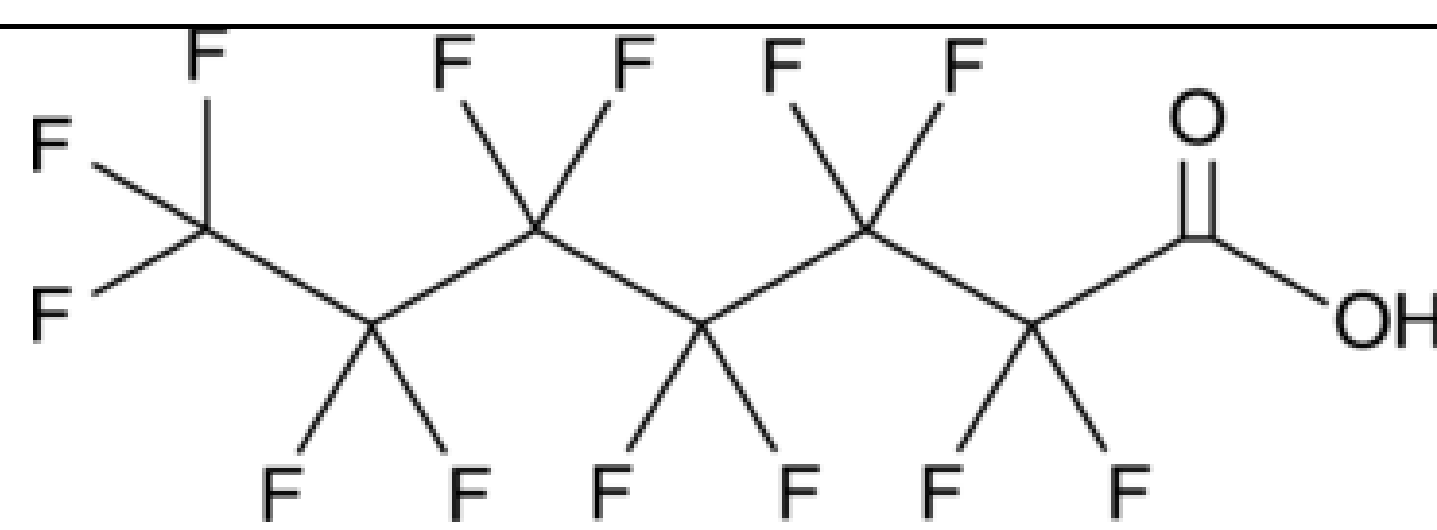

PFOA	$CF_3(CF_2)_6COOH$	335-67-1	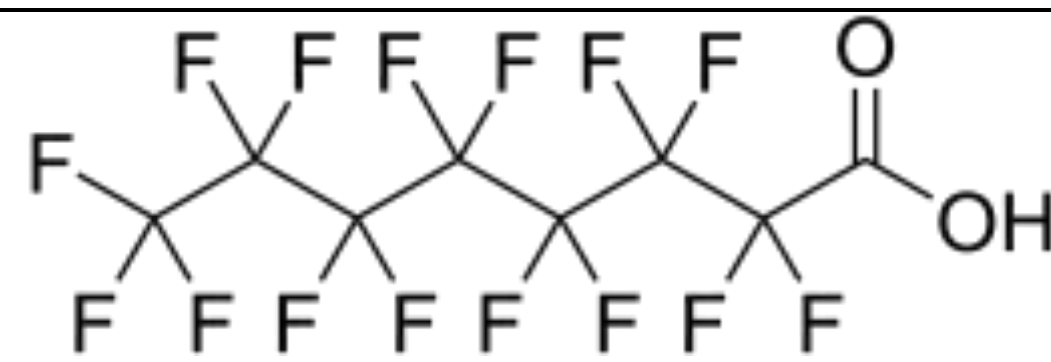
PFNA	$CF_3(CF_2)_7COOH$	375-95-1	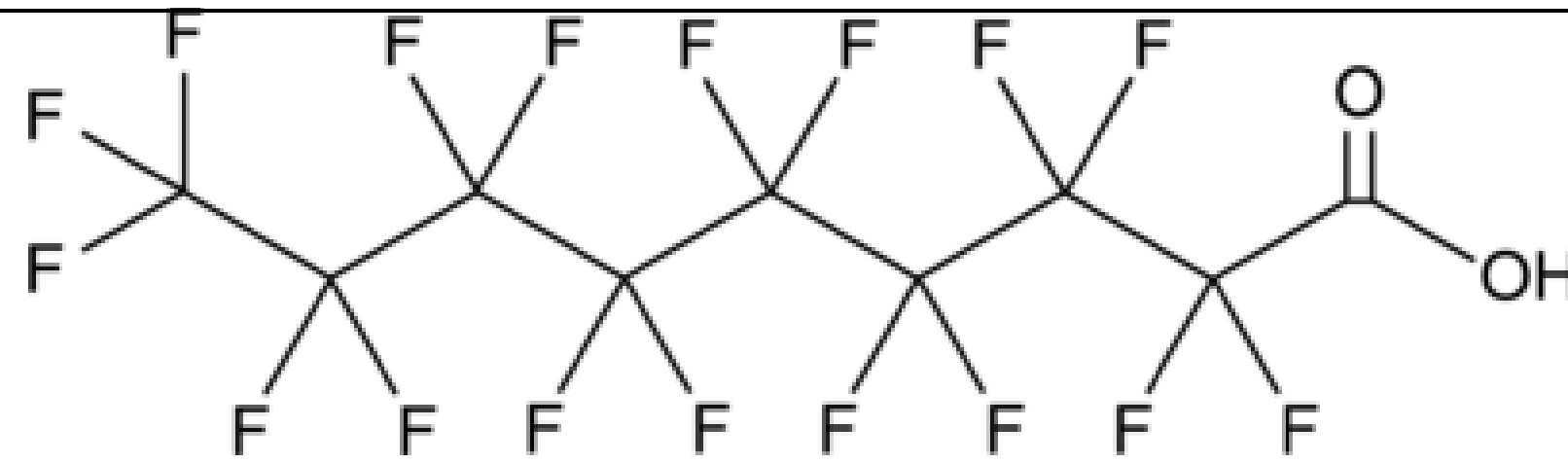
PFDA	$CF_3(CF_2)_8COOH$	335-76-2	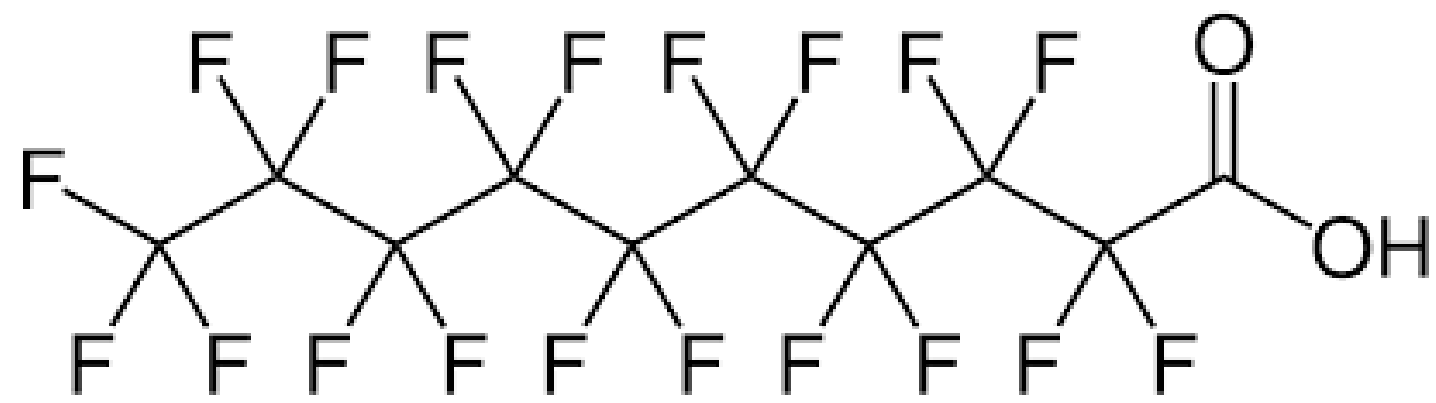
PFUdA	$CF_3(CF_2)_9COOH$	2058-94-8	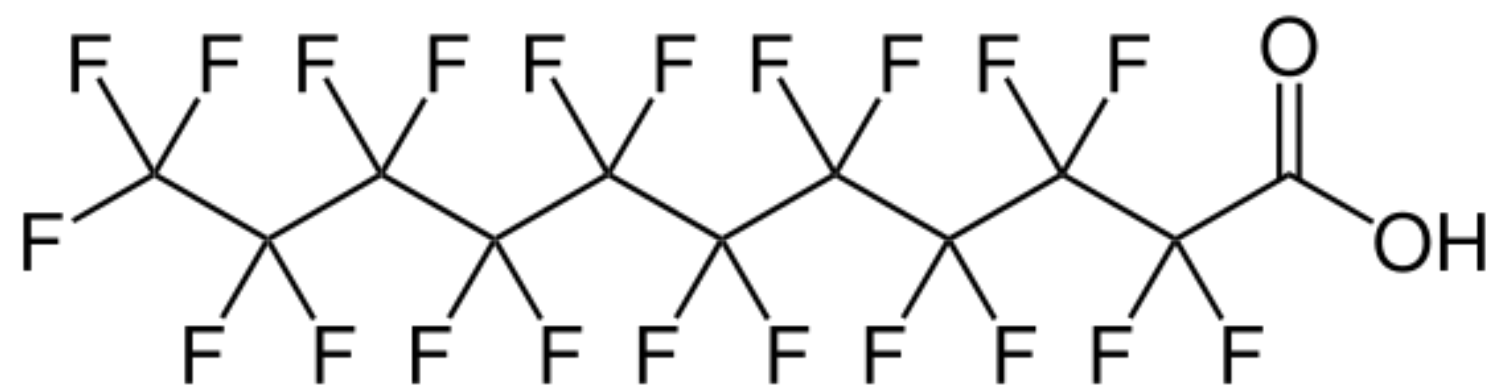
PFDoA	$CF_3(CF_2)_{10}COOH$	307-55-1	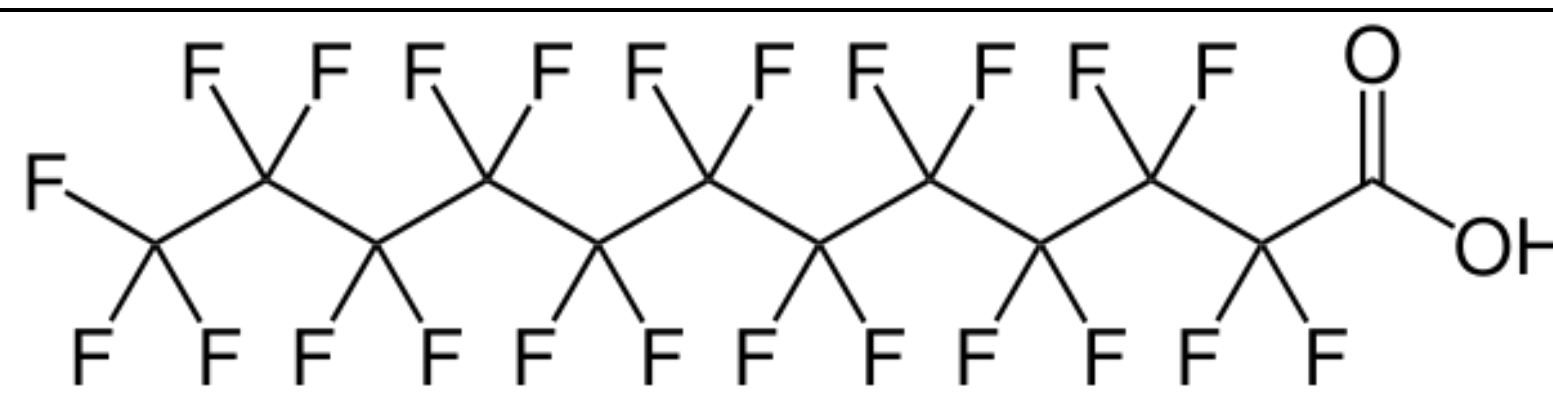
PFPrS	$CF_3(CF_2)_2SO_3H$	423-41-6	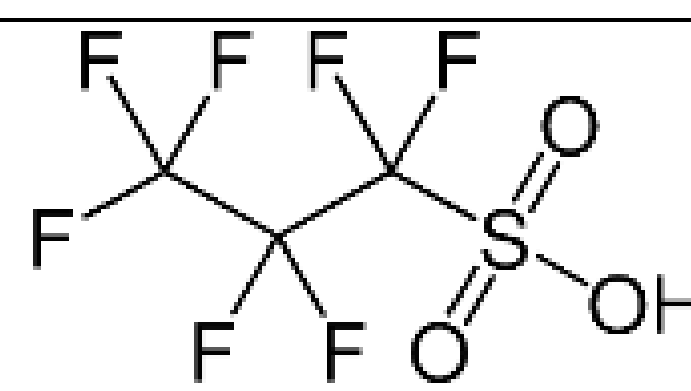
PFBS	$CF_3(CF_2)_3SO_3H$	375-73-5	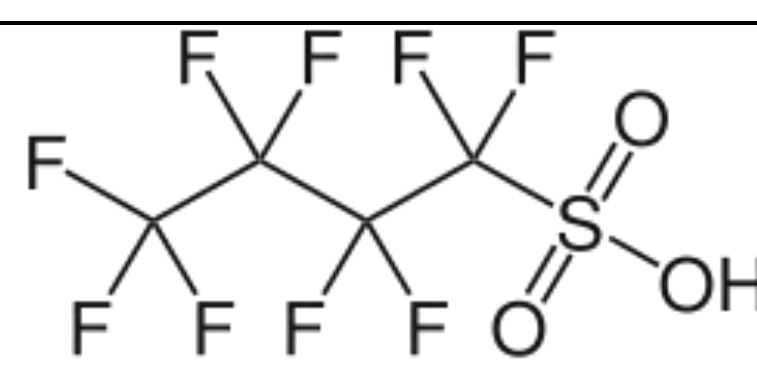

PFHxS	$CF_3(CF_2)_5SO_3H$	355-46-4	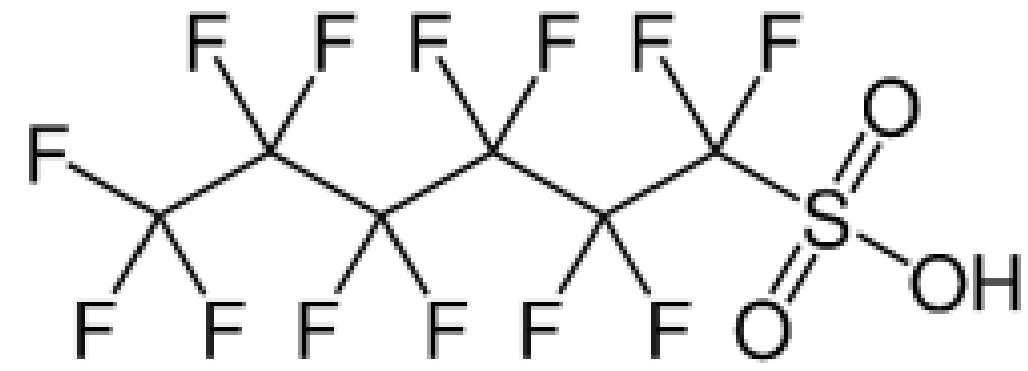
PFOS	$CF_3(CF_2)_7SO_3H$	1763-23-1	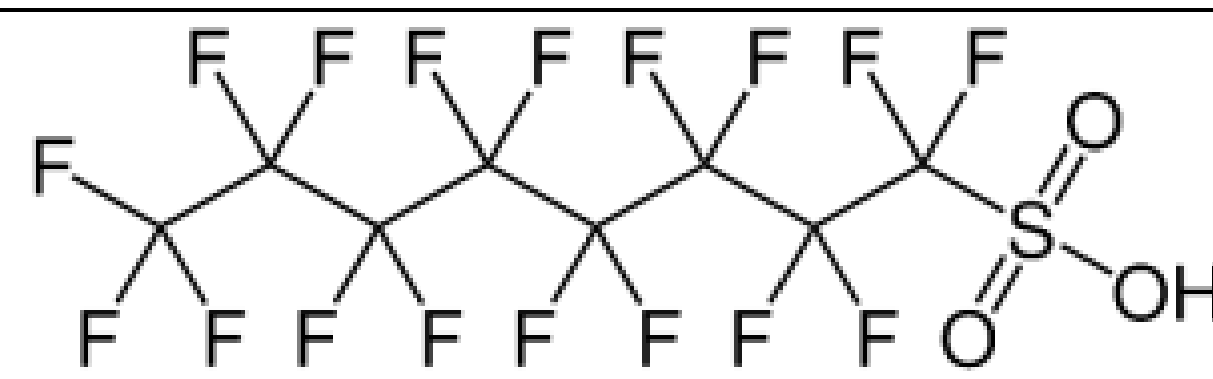
4:2 FTOH	$CF_3(CF_2)_3CH_2CH_2OH$	51756-80-0	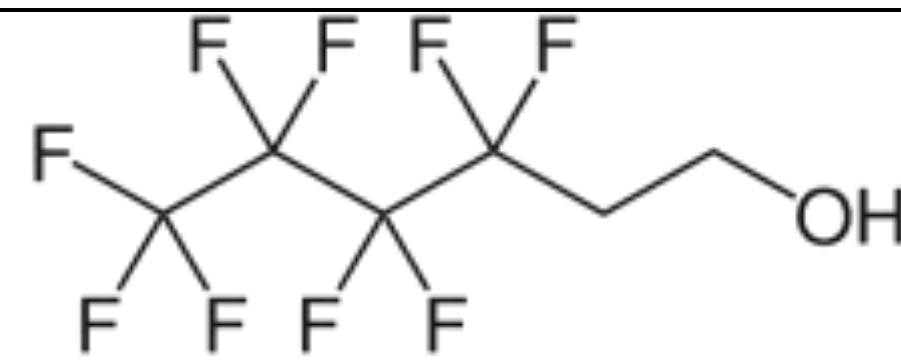
6:2 FTOH	$CF_3(CF_2)_5CH_2CH_2OH$	647-42-7	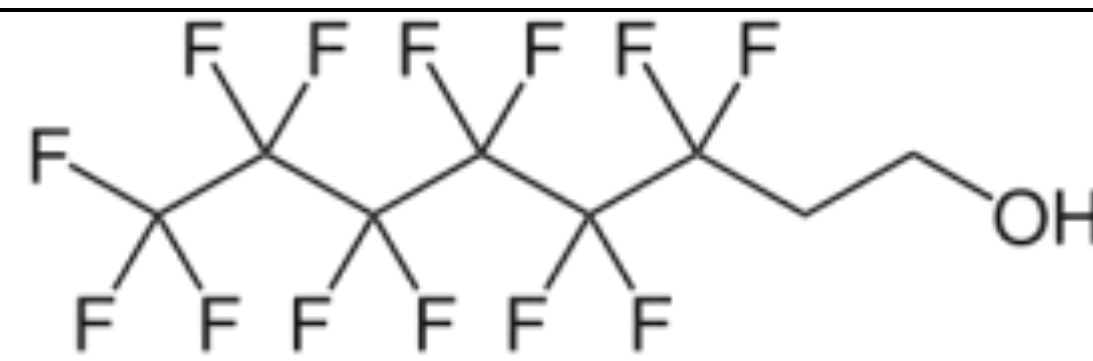
8:2 FTOH	$CF_3(CF_2)_7CH_2CH_2OH$	678-39-7	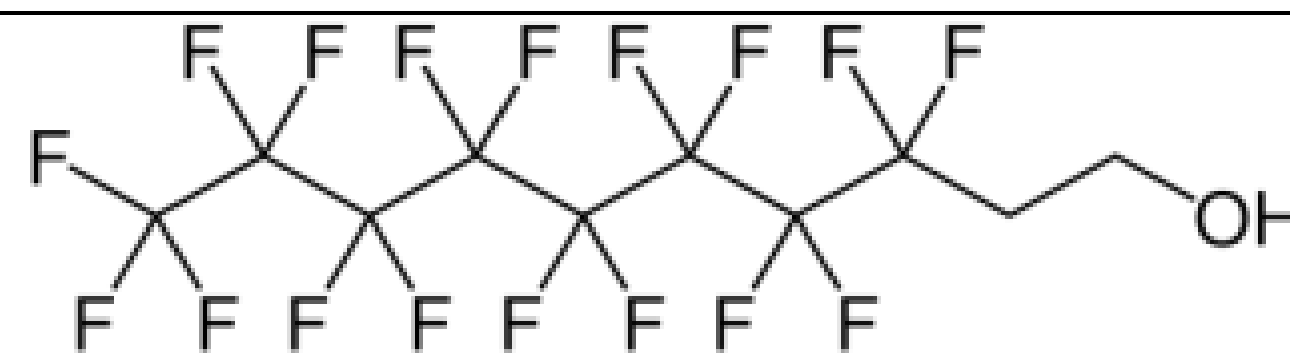
DONA	$CF_3O(CF_2)_3OCFHCF_2COOH$	919005-14-4	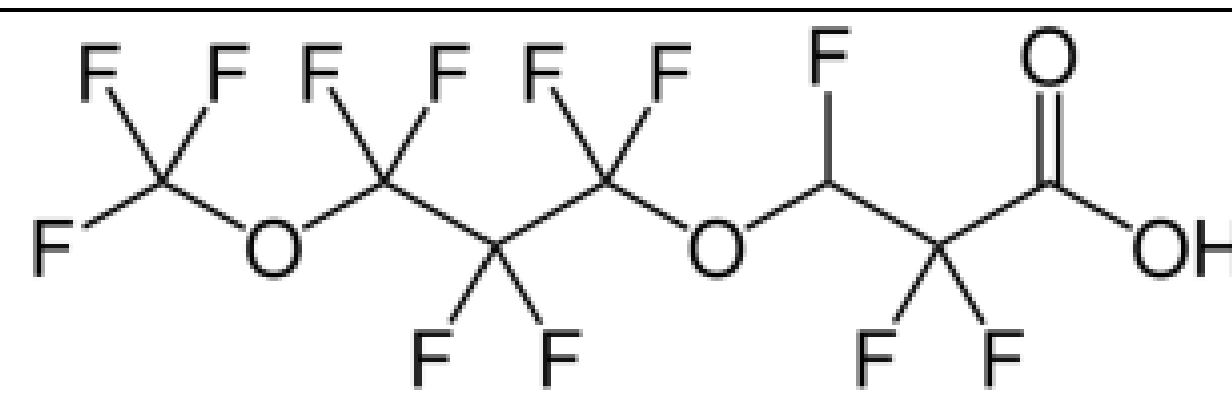
4:2 FTS	$CF_3(CF_2)_3(CH_2)_2SO_3H$	757124-72-4	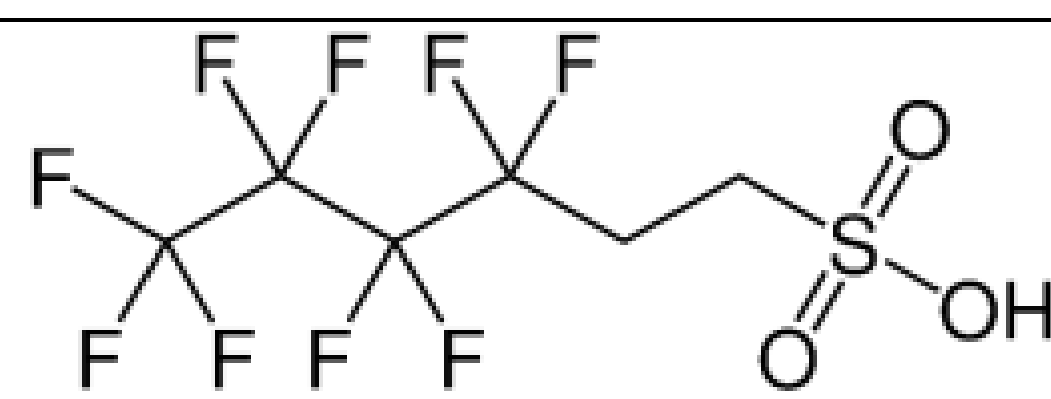
6:2 FTS	$CF_3(CF_2)_5(CH_2)_2SO_3H$	27619-97-2	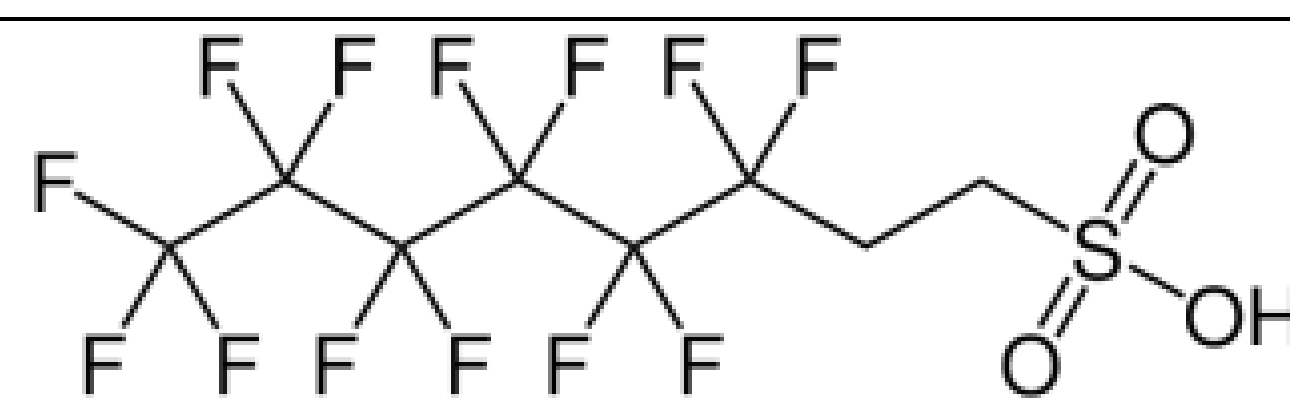

8:2 FTS	$CF_3(CF_2)_7(CH_2)_2SO_3H$	39108-34-4	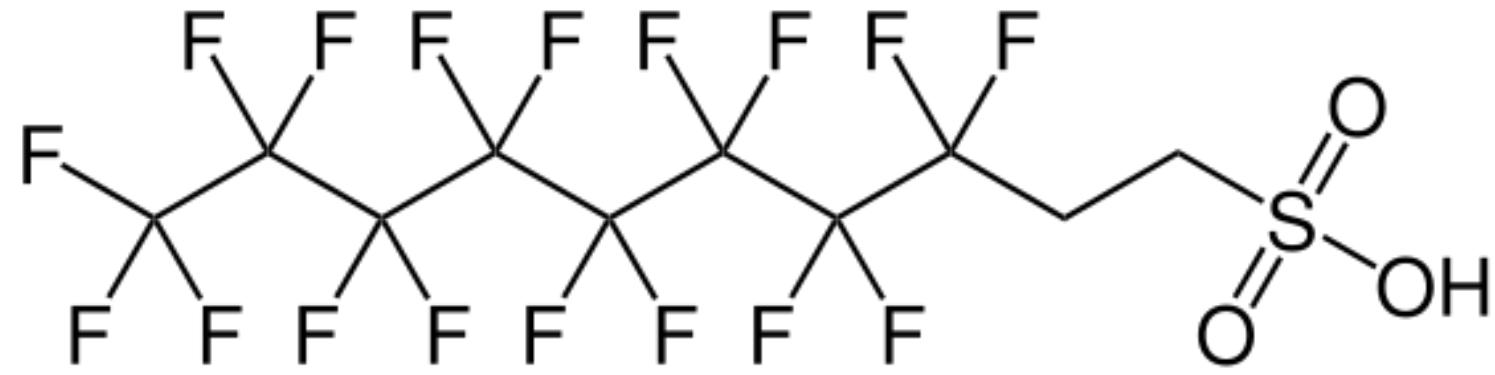
HFPO-DA	$CF_3(CF_2)_2OCF(CF_3)COOH$	62037-80-3	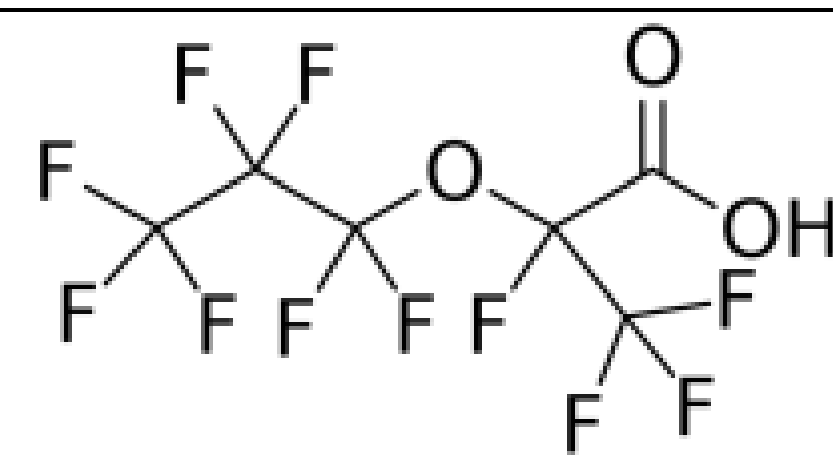

Table S3. Composition of the calibration standard used to calibrate I-CIMS and the ion form used for quantification.

Compound	Calibration Standard Concentration (M)	Source	Ion species used for quantification
Trifluoroacetic acid	1.3×10^{-4}	99%, TCI America	$[M-H]^-$
Perfluoropropionic acid	9.5×10^{-5}	97%, Thermo Scientific	$[M-H]^-$
Perfluorobutanoic acid	7.7×10^{-5}	99%, Thermo Scientific	$[M-H]^-$
Perfluoropentanoic acid	6.5×10^{-5}	97%, Thermo Scientific	$[M-H]^-$
Perfluorohexanoic acid	5.6×10^{-5}	98%, TCI America	$[M-H]^-$
Perfluoroheptanoic acid	4.9×10^{-5}	98%, TCI America	$[M-H]^-$
Perfluorooctanoic acid	4.8×10^{-5}	99%, Strem	$[M-H]^-$
Perfluorononanoic acid	3.9×10^{-5}	99%, Sigma Organic Chemistry	$[M-H]^-$
Perfluorodecanoic acid	3.3×10^{-5}	99%, Sigma Organic Chemistry	$[M-H]^-$
Dodecafluoro-4,8-dioxanonanoic acid	2.7×10^{-5}	Cambridge Isotope Laboratories	$[M-H]^-$
4:2 Fluorotelomer alcohol	3.8×10^{-5}	Accustandard, Inc.	$[M-I]^-$
6:2 Fluorotelomer alcohol	2.7×10^{-5}	Accustandard, Inc.	$[M-I]^-$
8:2 Fluorotelomer alcohol	2.2×10^{-5}	Accustandard, Inc.	$[M-H]^-$
Perfluorobutane sulfonic acid	3.3×10^{-5}	Cambridge Isotope Laboratories	$[M-H]^-$
Perfluorooctane sulfonic acid	2.0×10^{-5}	Accustandard, Inc.	$[M-H]^-$
4:2 fluototelomer sulfonate	3.1×10^{-5}	Accustandard, Inc.	$[M-I]^-$
6:2 fluorotelomer sulfonate	2.3×10^{-5}	Accustandard, Inc.	$[M-H]^-$
8:2 fluorotelomer sulfonate	1.9×10^{-5}	Accustandard, Inc.	$[M-H]^-$

Table S4. FIGAERO program used for calibration of PFAS compounds. PFAS calibration standards were deposited on the filter while it was exposed during the 1-minute XCH mode at the end of the cycle.

Time (min)	State	Heater Setpoint (°C)	Actuator Position	Desorption Gas Flow	Fan
0	Temp Ramp	250	DES	2000	OFF
5	Temp Soak	250	DES	2000	OFF
10	Cooling	25	DES	0	ON
11	Cooling	25	DES	0	ON
24	Cooling	25	XCH	0	ON
25	Cooling	25	XCH	0	ON

Table S5. List of targeted analytes and corresponding internal standards used for quantification.

Analyte abbreviation	Analyte name	Formula	CAS #	Internal dilution standard (EIS)	Internal performance standard (NIS)
**Perfluoroalkyl carboxylic acids**					
PFBA	Perfluoro-n-butanoic acid	$C_4HF_7O_2$	375-22-4	$^{13}C_4$ - PFBA	$^{13}C_3$ - PFBA
PFPeA	Perfluoro-n-pentanoic acid	$C_5HF_9O_2$	2706-90-3	$^{13}C_5$ - PFPeA	$^{13}C_3$ - PFBA
PFHxA	Perfluoro-n-hexanoic acid	$C_6HF_{11}O_2$	307-24-4	$^{13}C_6$ - PFHxA	$^{13}C_6$ - PFOA
PFHpA	Perfluoro-n-heptanoic acid	$C_7HF_{13}O_2$	375-85-9	$^{13}C_7$ - PFHpA	$^{13}C_6$ - PFOA
PFOA	Perfluoro-n-octanoic acid	$C_8HF_{15}O_2$	335-67-1	$^{13}C_8$ - PFOA	$^{13}C_6$ - PFOA
PFNA	Perfluoro-n-nonanoic acid	$C_9HF_{17}O_2$	375-95-1	$^{13}C_9$ - PFNA	$^{13}C_6$ - PFOA
PFDA	Perfluoro-n-decanoic acid	$C_{10}HF_{19}O_2$	335-76-2	$^{13}C_9$ - PFDA	$^{13}C_6$ - PFOA
PFUdA	Perfluoro-n-undecanoic acid	$C_{11}HF_{21}O_2$	2058-94-8	$^{13}C_9$ - PFUdA	$^{13}C_6$ - PFOA
PFDoA	Perfluoro-n-dodecanoic acid	$C_{12}HF_{23}O_2$	307-55-1	$^{13}C_{12}$ - PFDoA	$^{13}C_6$ - PFOA
**Perfluoroalkyl sulfonic acids**					
PFBS	Perfluorobutane sulfonic acid	$C_4HF_9O_3S$	375-73-5	$^{13}C_4$ - PFBS	$^{13}C_5$ - PFOS
PFPeS	Perfluoropentane sulfonic acid	$C_5HF_{11}O_3S$	2706-91-4	$^{13}C_6$ - PFHxS	$^{13}C_5$ - PFOS
PFHxS	Perfluorohexane sulfonic acid	$C_6HF_{13}O_3S$	355-46-4	$^{13}C_6$ - PFHxS	$^{13}C_5$ - PFOS
PFHpS	Perfluoroheptane sulfonic acid	$C_7HF_{15}O_3S$	375-92-8	$^{13}C_8$ - PFOS	$^{13}C_5$ - PFOS
PFOS	Perfluorooctane sulfonic acid	$C_8HF_{17}O_3S$	1763-23-1	$^{13}C_8$ - PFOS	$^{13}C_5$ - PFOS
**Fluorotelomer sulfonic acids**					
4:2 FTS	1H,1H,2H,2H-Perfluorohexane sulfonic acid	$C_6H_5F_9O_3S$	757124-72-4	$^{13}C_2D_4$ - 4:2 FTS	$^{13}C_5$ - PFOS
6:2 FTS	1H,1H,2H,2H-Perfluorooctane sulfonic acid	$C_8H_5F_{13}O_3S$	27619-97-2	$^{13}C_2D_4$ - 6:2 FTS	$^{13}C_5$ - PFOS
8:2 FTS	1H,1H,2H,2H-Perfluorodecane sulfonic acid	$C_{10}H_5F_{17}O_3S$	39108-34-4	$^{13}C_2D_4$ - 8:2 FTS	$^{13}C_5$ - PFOS
**Per- and Polyfluoroether carboxylic acids**					
HFPO-DA	Hexafluoropropylene oxide-dimer acid	$C_6HF_{11}O_3$	13252-13-6	$^{13}C_3$-HFPO-DA	$^{13}C_6$ - PFOA
ADONA	4,8-Dioxa-3H-perfluorononanoic acid	$C_7H_2F_{12}O_4$	919005-14-4	$^{13}C_3$-HFPO-DA	$^{13}C_6$ - PFOA
PFMPA	Perfluoro-3-methoxypropanoic acid	$C_4HF_7O_3$	377-73-1	$^{13}C_5$ - PFPeA	$^{13}C_3$ - PFBA
PFMBA	Perfluoro-4-methoxybutanoic acid	$C_5HF_9O_3$	863090-89-5	$^{13}C_5$ - PFPeA	$^{13}C_3$ - PFBA
NFDHA	Nonafluoro-3,6-dioxaheptanoic acid	$C_5HF_9O_4$	151772-58-6	$^{13}C_6$ - PFHxA	$^{13}C_6$ - PFOA
**Ether sulfonic acids**					
9Cl-PF3ONS	9-Chlorohexadecafluoro-3-oxanonane-1-sulfonic acid	$C_8HClF_{16}O_4S$	756426-58-1	$^{13}C_3$-HFPO-DA	$^{13}C_5$ - PFOS
11Cl-PF3OUdS	11-Chloroeicosafluoro-3-oxaundecane-1-sulfonic acid	$C_{10}HClF_{20}O_4S$	763051-92-9	$^{13}C_3$-HFPO-DA	$^{13}C_5$ - PFOS
PFEESA	Perfluoro (2-ethoxyethane)sulfonic acid	$C_4HF_9O_4S$	113507-82-7	$^{13}C_6$ - PFHxA	$^{13}C_5$ - PFOS

Table S6. PFAS extraction efficiency (recovery, %) of quartz fiber filters were calculated by spiking the filter with 100 µL of 10 ng/mL native standards. The filter was extracted identical to samples to a final volume of 5 mL. Recovery was calculated by dividing the concentration from each filter by the expected concentration (0.2 ng/mL).

**Analyte**	**Recovery (%)**
**Perfluoroalkyl carboxylic acids**	
PFBA	137
PFPeA	142
PFHxA	69
PFHpA	151
PFOA	128
PFNA	134
PFDA	132
PFUdA	134
PFDoA	132
**Perfluoroalkyl sulfonic acids**	
PFBS	106
PFPeS	129
PFHxS	147
PFHpS	112
PFOS	58
**Fluorotelomer sulfonic acids**	
4:2 FTS	73
6:2 FTS	121
8:2 FTS	100
**Per- and Polyfluoroether carboxylic acids**	
HFPO-DA	129
ADONA	163
PFMPA	128
PFMBA	144
NFDHA	133
**Ether sulfonic acids**	
9Cl-PF3ONS	161
11Cl-PF3OUdS	146
PFEESA	117

Table S7. Recovery of extracted internal standard (EIS) compounds were calculated with respect to the NIS internal standards.

EIS compound	Recovery (%)
$^{13}C_4$ - PFBA	18
$^{13}C_5$ - PFPeA	117
$^{13}C_6$ - PFHxA	152
$^{13}C_7$ - PFHpA	154
$^{13}C_8$ - PFOA	191
$^{13}C_9$ - PFNA	160
$^{13}C_9$ - PFDA	160
$^{13}C_9$ - PFUdA	141
$^{13}C_{12}$ - PFDoA	131
$^{13}C_4$ - PFBS	108
$^{13}C_6$ - PFHxS	117
$^{13}C_8$ - PFOS	138
$^{13}C_2D_4$ - 4:2 FTS	83
$^{13}C_2D_4$ - 6:2 FTS	84
$^{13}C_2D_4$ - 8:2 FTS	106
$^{13}C_3$ -HFPO-DA	130

Table S8. Analytical detection limit (ADL) is calculated by following the EPA method by running a low concentration calibration standard (0.5 ng/mL) for seven times in succession. The ADL is the product of standard deviation of the seven runs and the student's t-value appropriate for a single-tailed 99th percentile t statistics (3.143).

Analyte	ADL (pg/mL)	ADL (pg/m^3)
**Perfluoroalkyl carboxylic acids**		
PFBA	195	98
PFPeA	25	13
PFHxA	53	27
PFHpA	43	22
PFOA	2827	1414
PFNA	13	7
PFDA	38	19
PFUdA	35	18
PFDoA	9	5
**Perfluoroalkyl sulfonic acids**		
PFBS	21	11
PFPeS	50	25
PFHxS	27	14
PFHpS	110	55
PFOS	55	28
**Fluorotelomer sulfonic acids**		
4:2 FTS	77	39
6:2 FTS	107	54
8:2 FTS	141	71
**Per- and Polyfluoroether carboxylic acids**		
HFPO-DA	101	51
ADONA	37	19
PFMPA	49	25
PFMBA	36	18
NFDHA	40	20
**Ether sulfonic acids**		
9Cl-PF3ONS	80	40
11Cl-PF3OUdS	48	24
PFEESA	27	14

***** *ADL in air concentrations were determined from analytical method detection limit, multiplying by final extract volume (5 mL) and dividing by sample air volume of 10 m^3 (volume calculated by sampling flow rate of 1 L/min, sample duration of 14 days and half filter sampled per day.)*

Table S9. Reported field blank concentrations subtracted from sample concentrations if significantly higher than 0. Field measurement detection limit (MDL_b) is the sum of average and 3σ of the field blank concentrations. The MDL is the larger of MDL_b or ADL. MDL for analytes expressed as ADL are marked with * and N.D. indicates analytes not detected in the blank sample.

**Analyte**	**Field Blank concentration (pg/mL)**	**Field Blank concentration (pg/m^3)**	**MDL_b (pg/mL)**	**MDL_b (pg/m^3)**	**MDL (pg/m^3)**
**Perfluoroalkyl carboxylic acids**					
PFBA	5246	2623	5600	2800	2800
PFPeA	N.D	N.D	N.D	N.D	13*
PFHxA	351	176	415	208	208
PFHpA	17	9	20	10	22*
PFOA	1574	787	2977	1489	1489
PFNA	N.D	N.D	N.D	N.D	7*
PFDA	8	4	31	16	19*
PFUdA	N.D	N.D	N.D	N.D	18*
PFDoA	6	3	7	4	5*
**Perfluoroalkyl sulfonic acids**					
PFBS	N.D	N.D	N.D	N.D	11*
PFPeS	N.D	N.D	N.D	N.D	25*
PFHxS	N.D	N.D	N.D	N.D	14*
PFHpS	N.D	N.D	N.D	N.D	55*
PFOS	N.D	N.D	N.D	N.D	28*
**Fluorotelomer sulfonic acids**					
4:2 FTS	16	8	25	13	39*
6:2 FTS	N.D	N.D	N.D	N.D	54*
8:2 FTS	N.D	N.D	N.D	N.D	71*
**Per- and Polyfluoroether carboxylic acids**					
HFPO-DA	6	3	22	11	51*
ADONA	N.D	N.D	N.D	N.D	19*
PFMPA	N.D	N.D	N.D	N.D	25*
PFMBA	N.D	N.D	N.D	N.D	18*
NFDHA	N.D	N.D	N.D	N.D	20*
**Ether sulfonic acids**					
9Cl-PF3ONS	N.D	N.D	N.D	N.D	40*
11Cl-PF3OUdS	N.D	N.D	N.D	N.D	24*
PFEESA	N.D	N.D	N.D	N.D	14*

Table S10. CIMS calibration factors and detection limits for PFAS compounds in this study as well as previously reported in literature.

Compound	Calibration Factor (Hz pptv^{-1})	LOD (pptv)	Reference	Instrument
TFA	265 ± 79.5	0.1	Young et al., 2024[6]	Acetate CIMS
	100 ± 10	2.0	Matilla et al., 2024a[11, 17]	I-CIMS
	30 ± 10	0.4	Mattila et al., 2024b[11, 17]	I-CIMS
	**70.3 ± 15.8**	**0.05**	**This Study**	**I-CIMS**
PFPrA	700.1 ± 525.1	0.01	Young et al., 2024[6]	Acetate CIMS
	150 ± 20	5.0	Matilla et al., 2024a[17]	I-CIMS
	60 ± 10	0.7	Mattila et al., 2024b[11]	I-CIMS
	**141.7 ± 29.5**	**0.02**	**This Study**	I-CIMS
PFBA	228.1 ± 171.1	0.01	Young et al., 2024[6]	Acetate CIMS
	130 ± 20	1.0	Matilla et al., 2024a[17]	I-CIMS
	30 ± 10	0.3	Mattila et al., 2024b[11]	I-CIMS
	24.8 ± 3.9	1.4	Riedel et al., 2019	I-CIMS
	20.7 ± 0.3	1.3	Davern et al., 2024	I-CIMS
	**95.4 ± 33.0**	**0.01**	**This Study**	**I-CIMS**
PFPeA	111.7 ± 83.8	0.006	Young et al., 2024[6]	Acetate CIMS
	100 ± 10	0.5	Matilla et al., 2024a[17]	I-CIMS
	30 ± 10	0.2	Mattila et al., 2024b[11]	I-CIMS
	**60.8 ± 15.2**	**0.01**	**This Study**	I-CIMS
PFHxA	87.6 ± 65.7	0.013	Young et al., 2024[6]	Acetate CIMS
	130 ± 20	0.3	Matilla et al., 2024a[17]	I-CIMS
	30 ± 80	-	Mattila et al., 2024b[11]	I-CIMS
	**83.9 ± 17.1**	**0.01**	**This Study**	**I-CIMS**

PFHpA	60 ± 10	0.5	Mattila et al., 2024b	I-CIMS
	**63.1 ± 25.3**	**0.01**	**This Study**	I-CIMS
PFOA	2.9 ± 0.6	5.9	Riedel et al., 2019	I-CIMS
	30 ± 10	0.3	Mattila et al., 2024b[11]	I-CIMS
	12.4 ± 0.2	0.7	Davern et al., 2024	I-CIMS
	**31.9 ± 13.3**	**0.03**	**This Study**	**I-CIMS**
PFNA	**30.9 ± 27.2**	**0.01**	**This Study**	I-CIMS
PFDA	**51.7 ± 5.7**	**0.01**	**This Study**	I-CIMS
DONA	**28.2 ± 4.7**	**0.04**	**This Study**	I-CIMS
4:2 FTOH	3.0 ± 0.7	7.9	Riedel et al., 2019	I-CIMS
	13 ± 1	0.5	Matilla et al., 2024a[17]	I-CIMS
	4.3 ± 0.01	3.1	Davern et al., 2024	I-CIMS
	**24.5 ± 16.7**	**0.05**	**This Study**	I-CIMS
6:2 FTOH	5.1 ± 0.3	2.1	Riedel et al., 2019	I-CIMS
	26 ± 1	0.2	Matilla et al., 2024a[17]	I-CIMS
	6.1 ± 0.03	1.5	Davern et al., 2024	I-CIMS
	**24.2 ± 12.5**	**0.03**	**This Study**	I-CIMS
8:2 FTOH	9.1 ± 0.8	1.8	Riedel et al., 2019	I-CIMS
	6.2 ± 0.06	2.0	Davern et al., 2024	I-CIMS
	**94.5 ± 7.8**	**0.03**	**This Study**	I-CIMS
PFBS	**29.4 ± 8.5**	**0.01**	**This Study**	I-CIMS
PFOS	**66.2 ± 31.3**	**0.01**	**This Study**	I-CIMS
4:2 FTS	**23.2 ± 10.8**	**0.02**	**This Study**	I-CIMS
6:2 FTS	**35.7 ± 17.1**	**0.02**	**This Study**	I-CIMS
8:2 FTS	**75.0 ± 42.4**	**0.01**	**This Study**	I-CIMS

Table S11. Concentration of PFAS analytes detected in Houston, where values have been field blank subtracted. N.D indicates that compounds were not detected in the sample and values with "<" indicates that the PFAS analyte were detected but less than MDL

**Analyte**	**Mean (pg/m^3) (n=4)**	**RSD (%) (n=4)**
PFBA	**1545**	3
PFPeA	**34**	2
PFHxA	**59**	7
PFHpA	**14**	8
PFOA	**128**	3
PFNA	**24**	4
PFDA	<19	
PFUdA	**7**	7
PFDoA	**5**	12
PFBS	**4**	11
PFPeS	N.D	N.D
PFHxS	<14	
PFHpS	<55	
PFOS	**4**	20
4:2FTS	<39	
6:2FTS	**323**	9
8:2FTS	N.D	N.D
HFPO-DA	**18**	17
ADONA	N.D	N.D
PFMPA	N.D	N.D
PFMBA	N.D	N.D
NFDHA	N.D	N.D
9Cl-PF3ONS	N.D	N.D
11Cl-PF3OUdS	N.D	N.D
PFEESA	N.D	N.D

330 **Table S12.** All PFAS compounds detected above LOD with I-CIMS and LC-ESI Orbitrap MS
331 during this study.

Compound	Full name	Formula	Gas-phase I-CIMS	Particle-phase I-CIMS	Particle-phase LC-MS	Reference
TFA	Trifluoroacetic acid	$C_2HF_3O_2$	✓	✓	✓	Mattila et al., 2024[11]; Young et al., 2024[6]; Martin et al., 2003[18]
PFPrA	Perfluoropropionic acid	$C_3HF_5O_2$	✓	✓		Young et al., 2024[6]
PFBA	Perfluorobutanoic acid	$C_4HF_7O_2$	✓	✓	✓	Ahrens et al., 2012[19]; Yu et al., 2020[20]; Young et al., 2024[6]; Guerra de Navarro et al., 2025[21]
PFBS	Perfluorobutanesulfonic acid	$C_4HF_9O_3S$			✓	Casas et al., 2020[22]
PFPeA	Perfluoropentanoic acid	$C_5HF_9O_2$	✓	✓	✓	Young et al., 2024[6]
4:2 FTS	4:2 fluorotelomer sulfonate	$C_6H_5F_9O_3S$			✓	Kourtchev et al., 2022[23]
4:2 FTOH	4:2 fluorotelomer alcohol	$C_6H_5F_9O$	✓			Mattila et al., 2024[11]
PFHxA	Perfluorohexanoic acid	$C_6HF_{11}O_2$	✓	✓	✓	Barber et al., 2007[24]; Ahrens et al., 2012[19]; Yu et al., 2018[20]; Young et al., 2024[6]
HFPO-DA (GenX)	Hexafluoropropylene oxide dimer acid	$C_6HF_{11}O_3$			✓	Lin et al., 2020[25]
PFHpA	Perfluoroheptanoic acid	$C_7HF_{13}O_2$	✓	✓	✓	Ahrens et al., 2012[19]; Yu et al., 2020[20]
DONA	4,8-dioxa-3H-perfluorononanoic acid	$C_7H_2F_{12}O_4$		✓		Asakawa et al., 2025[26]
6:2 FTOH	6:2 fluorotelomer alcohol	$C_8H_5F_{13}O$	✓	✓		Ahrens et al., 2012[19]; Guo et al., 2018[27]; Lin et al., 2020[25]; Yu et al., 2020[20]

PFOA	Perfluorooctaonic acid	$C_8HF_{15}O_2$	✓	✓	✓	Ahrens et al., 2012[19]; Yu et al., 2020[20]; Zhou et al., 2021[28]
6:2 FTS	6:2 fluorotelomer sulfonate	$C_8H_5F_{13}O_3S$	✓	✓	✓	Barber et al., 2007[24]; Lin et al., 2020[25]; Yu et al., 2020[20]; Kourtchev et al., 2022[23]
PFNA	Perfluorononanoic acid	$C_9HF_{17}O_2$	✓	✓	✓	Ahrens et al., 2012[19]; Lin et al., 2020[25]
8:2 FTOH	8:2 fluorotelomer alcohol	$C_{10}H_5F_{17}O$	✓	✓		Ahrens et al., 2012[19]; Jahnke et al., 2009[29]
PFOS	Perfluorooctane sulfonic acid	$C_8HF_{17}O_3S$	✓			Ahrens et al., 2012[19]; Lin et al, 2020[25]; Yu et al., 2020[20]
PFDA	Perfluorodecanoic acid	$C_{10}HF_{19}O_2$	✓	✓	✓	Ahrens et al., 2012[19]; Guo et al., 2018[27]; Lin et al., 2020[25]
8:2 FTS	8:2 fluorotelomer sulfonate	$C_{10}H_5F_{17}O_3S$	✓			Lin et al., 2020[25]; Yu et al., 2018[20]; Kourtchev et al., 2022[23]
PFUdA	Perfluoroundecanoic acid	$C_{10}HF_{21}O_2$			✓	Barber et al., 2007[24]
PFDoA	Perfluorododecanoic acid	$C_{12}HF_{23}O_2$			✓	Barber et al., 2007[24]

Table S13. Median concentrations, range of concentrations, and detection frequencies of quantified gas-phase PFAS compounds, meteorological factors and gas-phase atmospheric pollutants during the study period. For PFCAs, *n* denotes the total amount of measurement cycles where a high-resolution peak for that compound could be fit to the mass spectrum, and detection frequency indicates the percentage measurements that were above LOD.

	Median	Range	n	Detection Frequency
TFA	0.34 pptv	<LOD – 1.9 pptv	12784	93.5%
	1.57 ng m^{-3}	<LOD – 8.6 ng m^{-3}		
PFPrA	0.44 pptv	0.01 – 2.4 pptv	12677	100%
	3.0 ng m^{-3}	0.05 – 16.4 ng m^{-3}		
PFBA	0.054 pptv	<LOD – 0.27 pptv	13031	99.7%
	0.48 ng m^{-3}	<LOD – 2.4 ng m^{-3}		
PFPeA	0.087 pptv	<LOD – 1.0 pptv	12421	78.1%
	0.94 ng m^{-3}	<LOD – 10.7 ng m^{-3}		
PFHxA	0.028 pptv	<LOD – 0.16 pptv	12578	94.5%
	0.37 ng m^{-3}	<LOD – 2.1 ng m^{-3}		
PFHpA	0.062 pptv	<LOD – 0.51 pptv	13390	83.2%
	0.92 ng m^{-3}	<LOD – 7.6 ng m^{-3}		
PFOA	0.028 pptv	<LOD – 0.12 pptv	12835	81.7%
	0.47 ng m^{-3}	<LOD – 2.1 ng m^{-3}		
PFNA	0.016 pptv	<LOD – 0.10 pptv	13067	62.0%
	0.31 ng m^{-3}	<LOD – 1.9 ng m^{-3}		
PFDA	0.01 pptv	<LOD – 0.051 pptv	13484	58.5%
	0.19 ng m^{-3}	<LOD – 1.1 ng m^{-3}		
4:2 FTOH	0.31 pptv	<LOD – 3.3 pptv	13817	90.9%
	3.3 ng m^{-3}	<LOD – 35.4 ng m^{-3}		
6:2 FTOH	0.21 pptv	<LOD – 1.0 pptv	13975	88.4%
	3.1 ng m^{-3}	<LOD – 15.4 ng m^{-3}		

8:2 FTOH	0.012 pptv	<LOD – 0.07 pptv	13298	71.5%
	0.2 ng m^{-3}	<LOD – 1.3 ng m^{-3}		
PFOS	0.01 pptv	<LOD – 0.04 pptv	13153	53.1%
	0.1 ng m^{-3}	<LOD – 0.8 ng m^{-3}		
6:2 FTS	0.03 pptv	<LOD – 0.11 pptv	13297	81.1%
	0.48 ng m^{-3}	<LOD – 2.1 ng m^{-3}		
8:2 FTS	0.01 pptv	<LOD – 0.03 pptv	13914	65.6%
	0.15 ng m^{-3}	<LOD – 0.6 ng m^{-3}		
Temperature	24 °C	11.3 – 33.9 °C	10628	
Relative Humidity	57.3 %	15.4 – 89.9 %	10628	
O_3	31.8 ppbv	<LOD – 78 ppbv	10628	
CO	156.8 ppbv	81.2 – 395.2 ppbv	10628	
SO_2	0.30 ppbv	<LOD – 2.9 ppbv	14781	
NO_x	12.7 ppbv	<LOD – 45.9 ppbv	9183	
NH_3	4.2 ppbv	0.56 – 12.2 ppbv	1382	

Table S14. Median concentrations, range of concentrations, and detection frequencies of quantified particle-phase PFAS compounds during the study period. *n* denotes the total amount of measurement cycles where a high-resolution peak for that compound could be fit to the mass spectrum, and detection frequency indicates the percentage measurements that were above LOD.

	Median	Range	n	Detection Frequency
TFA	0.04 pptv	<LOD – 0.25 pptv	336	81%
	0.15 ng m^{-3}	<LOD – 1.2 ng m^{-3}		
PFPrA	0.34 pptv	0.02 – 0.24 pptv	349	100%
	0.23 ng m^{-3}	0.1– 1.6 ng m^{-3}		
PFBA	0.01 pptv	<LOD – 0.03 pptv	375	91%
	0.08 ng m^{-3}	<LOD – 0.25 ng m^{-3}		
PFPeA	0.02 pptv	<LOD – 0.22 pptv	344	98%
	0.06 ng m^{-3}	<LOD – 1.4 ng m^{-3}		
PFHxA	0.01 pptv	<LOD – 0.03 pptv	378	92%
	0.07 ng m^{-3}	<LOD – 0.3 ng m^{-3}		
PFHpA	0.02 pptv	<LOD – 0.11 pptv	362	100%
	0.30 ng m^{-3}	<LOD – 1.6 ng m^{-3}		
PFOA	0.011 pptv	<LOD – 0.05 pptv	356	100%
	0.19 ng m^{-3}	<LOD – 0.79 ng m^{-3}		
PFNA	0.01 pptv	<LOD – 0.03 pptv	356	100%
	0.14 ng m^{-3}	<LOD – 0.43 ng m^{-3}		
PFDA	0.01 pptv	<LOD – 0.03 pptv	349	100%
	0.11 ng m^{-3}	<LOD – 0.48 ng m^{-3}		
DONA	0.03 pptv	<LOD – 0.15 pptv	348	100%
	0.42 ng m^{-3}	<LOD – 2.37 ng m^{-3}		
6:2 FTOH	0.06 pptv	<LOD – 0.18 pptv	388	100%
	0.82ng m^{-3}	<LOD – 2.6 ng m^{-3}		

8:2 FTOH	0.01 pptv	<LOD – 0.04 pptv	356	100%
	0.14 ng m^{-3}	<LOD – 0.62 ng m^{-3}		
6:2 FTS	0.02 pptv	<LOD – 0.06 pptv	365	99%
	0.29 ng m^{-3}	<LOD – 1.0 ng m^{-3}		

Table S15. Concentration of PFAS analytes detected in Huntsville for the four weeks studied, where values have been field blank subtracted. N.D indicates that compounds were not detected in the sample and values with "<" indicates that the PFAS analyte was detected but less than MDL

Analyte	06/30-07/14/2025 (pg/m^3) (n=4)	RSD (%) (n=4)	07/14-07/28/2025 (pg/m^3) (n=4)	RSD (%) (n=4)
PFBA	N.D	N.D	N.D	N.D
PFPeA	<13		<13	
PFHxA	**9**	14	**11**	3
PFHpA	**3**	22	**2**	13
PFOA	**41**	12	**32**	5
PFNA	**10**	4	**9**	6
PFDA	**16**	7	**17**	5
PFUdA	**18**	7	**24**	4
PFDoA	**17**	8	**35**	3
PFBS	N.D	N.D	N.D	N.D
PFPeS	N.D	N.D	N.D	N.D
PFHxS	N.D	N.D	N.D	N.D
PFHpS	N.D	N.D	N.D	N.D
PFOS	<28		<28	
4:2FTS	N.D	N.D	N.D	N.D
6:2FTS	**2**	11	**1**	18
8:2FTS	N.D	N.D	N.D	N.D
HFPO-DA	N.D	N.D	N.D	N.D
ADONA	N.D	N.D	N.D	N.D
PFMPA	N.D	N.D	N.D	N.D
PFMBA	N.D	N.D	N.D	N.D
NFDHA	N.D	N.D	N.D	N.D
9Cl-PF3ONS	N.D	N.D	N.D	N.D
11Cl-PF3OUdS	N.D	N.D	N.D	N.D
PFEESA	N.D	N.D	N.D	N.D

Table S16. 12 min gradient program for UPLC-ESI-MS/MS

Time (min)	A (%)	B (%)	Flow rate (mL/min)	Curve
0	98	2	0.35	5
0.2	98	2	0.35	2
4	80	20	0.4	7
7	45	55	0.4	8
9	25	75	0.4	8
10	5	95	0.4	6
10.4	98	2	0.4	9
11.8	98	2	0.4	7
12	98	2	0.35	1

Table S17. UPLC and Orbitrap MS parameters

**LC parameters**	
Analytical column	Thermoscientific Accucore C18+ reverse phase, 2.1 x 50 mm, 1.5 µm particle size
Delay column	Thermoscientific Hypersil GOLD™ C18 reverse phase, 3 x 50 mm, 1.9 µm particle size
Column temperature	40 ^{o}C
Mobile Phase A	2 mM Ammonium acetate in 95:5 water: acetonitrile
Mobile Phase B	Acetonitrile
Injection volume	2 µL
**MS parameters**	
Negative ion voltage	1000 V
Sheath gas	35 arb
Aux gas	5 arb
Sweep gas	1 arb
Ion transfer tube temperature	220 ^{o}C
Vaporizer temperature	450 ^{o}C
Orbitrap resolution	15,000
RF Lens	60 %
Max injection time	Auto
AGC target	Auto
HESI position	X = 0, Y= 1, Z = between L and M

Table S18. Ion quantification parameters for LC-ESI-MS/MS.

Analyte	Retention time (min)	Parent ion (m/z)	Quan ion (m/z)	Confirmation ion (m/z)	HCD energy (V)
PFBA	1.49	212.9792	168.9894		10
PFPeA	4.6	262.976	218.9855	68.6585	9
PFHxA	5.78	312.9728	268.983	118.9926	10
PFHpA	6.28	362.9696	318.9799	168.9894	11
PFOA	6.96	412.9664	368.9766	168.9892	13
PFNA	7.79	462.9632	418.9737	218.9862	12
PFDA	8.34	512.96	468.9708	218.9862	15
PFUdA	8.6	562.9568	518.9669	268.9843	11
PFDoA	8.83	612.9537	568.9644	318.9799	13
PFBS	5.73	298.943	79.9574	98.9558	32
PFPeS	6.37	348.9398	79.9574	98.9558	35
PFHxS	7.16	398.9366	79.9574	98.9558	35
PFHpS	8.03	448.9334	79.9574	98.9558	40
PFOS	8.46	498.9303	79.9574	98.9558	45
4:2 FTS	5.64	326.9744	306.986	80.9653	25
6:2 FTS	6.68	426.9679	406.9621	80.9653	26
8:2 FTS	8.16	526.9615	506.9556	80.9653	31
HFPO-DA	5.94	284.9779	168.9899	184.9842	6
ADONA	6.51	376.9689	250.9761		12
PFMPA	2.63	228.9741	84.9908		10
PFMBA	5.2	278.9709	84.9908		10
NFDHA	5.71	294.9658	200.9789		7
9Cl-PF3ONS	8.65	530.8956	350.9453		32
11Cl-PF3OUdS	9.23	630.8892	450.9388		36
PFEESA	6.01	314.9379	134.9875		25

REFERENCES

1. Erupe, M. E.; Benson, D. R.; Li, J.; Young, L.-H.; Verheggen, B.; Al-Refai, M.; Tahboub, O.; Cunningham, V.; Frimpong, F.; Viggiano, A. A.; Lee, S.-H., Correlation of aerosol nucleation rate with sulfuric acid and ammonia in Kent, Ohio: An atmospheric observation. *Journal of Geophysical Research: Atmospheres* **2010,** *115* (D23).
2. Tiszenkel, L.; Flynn, J. H.; Dodero, A.; Lee, S.-H., Sulfuric acid, base, and low-volatility organics contribute to aerosol nucleation in urban Houston. *Communications Earth & Environment* **2025,** *6* (1), 364.
3. Yu, H.; Lee, S. H., A chemical ionization mass spectrometer for the detection of atmospheric amines. *Environ. Chem.* **2012** *9*,190-201.
4. Dörich, R.; Eger, P.; Lelieveld, J.; Crowley, J. N., Iodide CIMS and m/z 62: the detection of HNO3 as NO3− in the presence of PAN, peroxyacetic acid and ozone. *Atmospheric Measurement Techniques* **2021,** *14* (8), 5319-5332.
5. Zhang, W.; Zhang, H., Secondary Ion Chemistry Mediated by Ozone and Acidic Organic Molecules in Iodide-Adduct Chemical Ionization Mass Spectrometry. *Analytical Chemistry* **2021,** *93* (24), 8595-8602.
6. Young, C. J.; Joudan, S.; Tao, Y.; Wentzell, J. J. B.; Liggio, J., High Time Resolution Ambient Observations of Gas-Phase Perfluoroalkyl Carboxylic Acids: Implications for Atmospheric Sources. *Environmental Science & Technology Letters* **2024,** *11* (12), 1348-1354.
7. Brophy, P.; Farmer, D. K., A switchable reagent ion high resolution time-of-flight chemical ionization mass spectrometer for real-time measurement of gas phase oxidized species: characterization from the 2013 southern oxidant and aerosol study. *Atmos. Meas. Tech.* **2015,** *8* (7), 2945-2959.
8. Ye, C.; Yuan, B.; Lin, Y.; Wang, Z.; Hu, W.; Li, T.; Chen, W.; Wu, C.; Wang, C.; Huang, S.; Qi, J.; Wang, B.; Wang, C.; Song, W.; Wang, X.; Zheng, E.; Krechmer, J. E.; Ye, P.; Zhang, Z.; Wang, X.; Worsnop, D. R.; Shao, M., Chemical characterization of oxygenated organic compounds in the gas phase and particle phase using iodide CIMS with FIGAERO in urban air. *Atmospheric Chemistry and Physics* **2021,** *21* (11), 8455-8478.
9. Lee, B. H.; Lopez-Hilfiker, F. D.; Mohr, C.; Kurtén, T.; Worsnop, D. R.; Thornton, J. A., An Iodide-Adduct High-Resolution Time-of-Flight Chemical-Ionization Mass Spectrometer: Application to Atmospheric Inorganic and Organic Compounds. *Environmental Science & Technology* **2014,** *48* (11), 6309-6317.
10. Davern, M. J.; West, G. V.; Eichler, C. M. A.; Turpin, B. J.; Zhang, Y.; Surratt, J. D., External liquid calibration method for iodide chemical ionization mass spectrometry enables quantification of gas-phase per- and polyfluoroalkyl substances (PFAS) dynamics in indoor air. *Analyst* **2024,** *149* (12), 3405-3415.
11. Mattila, J. M.; Offenberg, J. H., Measuring short-chain per- and polyfluoroalkyl substances in Central New Jersey air using chemical ionization mass spectrometry. *Journal of the Air & Waste Management Association* **2024,** *74* (8), 531-539.
12. Riedel, T. P.; Lang, J. R.; Strynar, M. J.; Lindstrom, A. B.; Offenberg, J. H., Gas-Phase Detection of Fluorotelomer Alcohols and Other Oxygenated Per- and Polyfluoroalkyl Substances by Chemical Ionization Mass Spectrometry. *Environmental Science & Technology Letters* **2019,** *6* (5), 289-293.

13. Rauniyar, N., Parallel Reaction Monitoring: A Targeted Experiment Performed Using High Resolution and High Mass Accuracy Mass Spectrometry. *International Journal of Molecular Sciences* **2015,** *16* (12), 28566-28581.
14. Benson, D. R.; Markovich, A.; Al-Refai, M.; Lee, S. H., A Chemical Ionization Mass Spectrometer for ambient measurements of Ammonia. *Atmospheric Measurement Techniques* **2010,** *3* (4), 1075-1087.
15. Tiszenkel, L.; Flynn, J. H.; Lee, S. H., Measurement report: Urban ammonia and amines in Houston, Texas. *Atmospheric Chemistry and Physics* **2024,** *24* (19), 11351-11363.
16. You, Y.; Kanawade, V. P.; de Gouw, J. A.; Guenther, A. B.; Madronich, S.; Sierra-Hernández, M. R.; Lawler, M.; Smith, J. N.; Takahama, S.; Ruggeri, G.; Koss, A.; Olson, K.; Baumann, K.; Weber, R. J.; Nenes, A.; Guo, H.; Edgerton, E. S.; Porcelli, L.; Brune, W. H.; Goldstein, A. H.; Lee, S. H., Atmospheric amines and ammonia measured with a Chemical Ionization Mass Spectrometer (CIMS). *Atmospheric Chemistry and Physics* **2014,** *14*, 12181-12194.
17. Mattila, J. M.; Krug, J. D.; Roberson, W. R.; Burnette, R. P.; McDonald, S.; Virtaranta, L.; Offenberg, J. H.; Linak, W. P., Characterizing Volatile Emissions and Combustion Byproducts from Aqueous Film-Forming Foams Using Online Chemical Ionization Mass Spectrometry. *Environmental Science & Technology* **2024,** *58* (8), 3942-3952.
18. Martin, J. W.; Mabury, S. A.; Wong, C. S.; Noventa, F.; Solomon, K. R.; Alaee, M.; Muir, D. C. G., Airborne Haloacetic Acids. *Environmental Science & Technology* **2003,** *37* (13), 2889-2897.
19. Ahrens, L.; Harner, T.; Shoeib, M.; Lane, D. A.; Murphy, J. G., Improved Characterization of Gas–Particle Partitioning for Per- and Polyfluoroalkyl Substances in the Atmosphere Using Annular Diffusion Denuder Samplers. *Environmental Science & Technology* **2012,** *46* (13), 7199-7206.
20. Yu, N.; Wen, H.; Wang, X.; Yamazaki, E.; Taniyasu, S.; Yamashita, N.; Yu, H.; Wei, S., Nontarget Discovery of Per- and Polyfluoroalkyl Substances in Atmospheric Particulate Matter and Gaseous Phase Using Cryogenic Air Sampler. *Environmental Science & Technology* **2020,** *54* (6), 3103-3113.
21. Guerra de Navarro, M.; Botero, M.; Brill, C.; Zurbaran, R.; Quinete, N., Assessment of particle-bound PFAS in ambient air from a coastal urban environment in South Florida. *Journal of Hazardous Materials* **2025,** *497*, 139797.
22. Casas, G.; Martínez-Varela, A.; Roscales, J. L.; Vila-Costa, M.; Dachs, J.; Jiménez, B., Enrichment of perfluoroalkyl substances in the sea-surface microlayer and sea-spray aerosols in the Southern Ocean. *Environmental Pollution* **2020,** *267*, 115512.
23. Kourtchev, I.; Hellebust, S.; Heffernan, E.; Wenger, J.; Towers, S.; Diapouli, E.; Eleftheriadis, K., A new on-line SPE LC-HRMS method for the analysis of Perfluoroalkyl and Polyfluoroalkyl Substances (PFAS) in PM2.5 and its application for screening atmospheric particulates from Dublin and Enniscorthy, Ireland. *Science of The Total Environment* **2022,** *835*, 155496.
24. Barber, J. L.; Berger, U.; Chaemfa, C.; Huber, S.; Jahnke, A.; Temme, C.; Jones, K. C., Analysis of per- and polyfluorinated alkyl substances in air samples from Northwest Europe. *Journal of Environmental Monitoring* **2007,** *9* (6), 530-541.
25. Lin, H.; Taniyasu, S.; Yamazaki, E.; Wei, S.; Wang, X.; Gai, N.; Kim, J. H.; Eun, H.; Lam, P. K. S.; Yamashita, N., Per- and Polyfluoroalkyl Substances in the Air Particles of Asia:

Levels, Seasonality, and Size-Dependent Distribution. *Environmental Science & Technology* **2020,** *54* (22), 14182-14191.

26. Asakawa, D.; Lin, H.; Ruan, Y.; Taniyasu, S.; Yeung, L. W. Y.; Tojo, T.; Ichihara, M.; Yamazaki, E.; Hanari, N.; Lam, P. K. S.; Yamashita, N., Characterizing Seasonal Patterns, Gas-Particle Partitioning, and Potential Sources of Airborne Per- and Polyfluoroalkyl Substances in Japan. *Environmental Science & Technology* **2025,** *59* (10), 5145-5154.

27. Guo, M.; Lyu, Y.; Xu, T.; Yao, B.; Song, W.; Li, M.; Yang, X.; Cheng, T.; Li, X., Particle size distribution and respiratory deposition estimates of airborne perfluoroalkyl acids during the haze period in the megacity of Shanghai. *Environmental Pollution* **2018,** *234*, 9-19.

28. Zhou, J.; Baumann, K.; Mead, R. N.; Skrabal, S. A.; Kieber, R. J.; Avery, G. B.; Shimizu, M.; DeWitt, J. C.; Sun, M.; Vance, S. A.; Bodnar, W.; Zhang, Z.; Collins, L. B.; Surratt, J. D.; Turpin, B. J., PFOS dominates PFAS composition in ambient fine particulate matter ($PM_{2.5}$) collected across North Carolina nearly 20 years after the end of its US production. *Environmental Science: Processes & Impacts* **2021,** *23* (4), 580-587.

29. Jahnke, A.; Barber, J. L.; Jones, K. C.; Temme, C., Quantitative trace analysis of polyfluorinated alkyl substances (PFAS) in ambient air samples from Mace Head (Ireland): A method intercomparison. *Atmospheric Environment* **2009,** *43* (4), 844-850.
